# Bicyclic Isoxazoline Derivatives: Synthesis and Evaluation of Biological Activity

**DOI:** 10.3390/molecules27113546

**Published:** 2022-05-31

**Authors:** Kseniya N. Sedenkova, Kristian S. Andriasov, Marina G. Eremenko, Yuri K. Grishin, Vera A. Alferova, Anna A. Baranova, Nikolay A. Zefirov, Olga N. Zefirova, Vladimir V. Zarubaev, Yulia A. Gracheva, Elena R. Milaeva, Elena B. Averina

**Affiliations:** 1 Department of Chemistry, Lomonosov Moscow State University, Leninskie Gory 1-3, 119991 Moscow, Russia; sedenkova@med.chem.msu.ru (K.N.S.); akristian@mail.ru (K.S.A.); eremenkomg@gmail.com (M.G.E.); grishin@nmr.chem.msu.ru (Y.K.G.); kolaz92@gmail.com (N.A.Z.); olgaz_13@mail.ru (O.N.Z.); jullina74@mail.ru (Y.A.G.); milaeva@med.chem.msu.ru (E.R.M.); 2 Gause Institute of New Antibiotics, B. Pirogovskaya 11, 119021 Moscow, Russia; alferovava@gmail.com (V.A.A.); anjabaranowa@list.ru (A.A.B.); 3 Shemyakin-Ovchinnikov Institute of Bioorganic Chemistry, Miklukho-Maklaya 16/10, 117997 Moscow, Russia; 4 Saint Petersburg Pasteur Research Institute of Epidemiology and Microbiology, 14 Mira St., 197101 Saint Petersburg, Russia; zarubaev@gmail.com

**Keywords:** isoxazolines, cyclooctanes, spiro compounds, bicyclic compounds, heterocyclization, influenza A (H1N1)

## Abstract

The application of non-planar scaffolds in drug design allows for the enlargement of the chemical space, and for the construction of molecules that have more effective target–ligand interactions or are less prone to the development of resistance. Among the works of the last decade, a literature search revealed spirothiazamenthane, which has served as a lead in the development of derivatives active against resistant viral strains. In this work, we studied the novel molecular scaffold, which resembles spirothiazamenthane, but combines isoxazoline as a heterocycle and cyclooctane ring as a hydrophobic part of the structure. The synthesis of new 3-nitro- and 3-aminoisoxazolines containing spiro-fused or 1,2-annelated cyclooctane fragments was achieved by employing 1,3-dipolar cycloaddition of 3-nitro-4,5-dihydroisoxazol-4-ol 2-oxide or tetranitromethane-derived alkyl nitronates with non-activated alkenes. A series of spiro-sulfonamides was obtained by the reaction of 3-aminoisoxazoline containing a spiro-fused cyclooctane residue with sulfonyl chlorides. Preliminary screening of the compounds for antiviral, antibacterial, antifungal and antiproliferative properties in vitro revealed 1-oxa-2-azaspiro[4.7]dodec-2-en-3-amine and 3a,4,5,6,7,8,9,9a-octahydrocycloocta[d]isoxazol-3-amine with activity against the influenza A/Puerto Rico/8/34 (H1N1) virus in the submicromolar range, and high values of selectivity index. Further study of the mechanism of the antiviral action of these compounds, and the synthesis of their analogues, is likely to identify new agents against resistant viral strains.

## 1. Introduction

A notable trend in modern medicinal and organic chemistry is the expansion of the structural diversity of non-planar scaffolds used for drug design purposes [1,2,3,4,5,6,7]. This allows for the enlargement of the chemical space, and for the construction of molecules with more effective target–ligand interactions. New non-standard 3D scaffolds may be less prone to the development of resistance, and therefore ideal for creating the next generation of antiviral, antibacterial and anticancer therapeutics. Among the works of the last decade, a literature search revealed spiro-heterocyclic compounds identified by systematic screening, which have served as attractive leads in the development of agents against resistant bacterial or viral strains [8,9,10].

**Figure 1:**
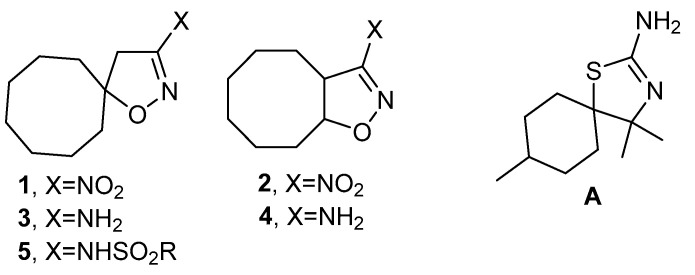
Spiro-heterocycle **A**, which served as lead for elaboration of agents that act on viral infections and compounds **1–5** synthesized and screened in this work.

In this work, we studied the novel molecular scaffold (see Figure 1, compounds **1,3,5**), which resembles the spirothiazamenthane **A [10]**, but combines isoxazoline as a heterocycle and cyclooctane ring as a hydrophobic part of the structure. The latter fragment is rarely used in drug design, although there are certain examples of its successful application for lead optimization (see [11] and references Therein).

To enlarge the library of organic compounds based on scaffold **A**, we proposed to vary the functionalities at C^3^ position and to modify the type of attachment of isoxazoline ring (see Figure 1, structures **2,4**). The nitro-group in position *3* was chosen, based on the preparative availability of 3-nitroisoxazoles and opportunities for its further modification to amines and sulfonamides [12,13]. Amines were of particular interest for antiviral activity, while sulfonamide derivatives were obtained with regard to the well-known interest in these derivatives in the search for novel antibiotics [14].

## 2. Results and Discussion

### 2.1. Synthesis of 3-Substituted Isoxazolines

**Scheme 1:**
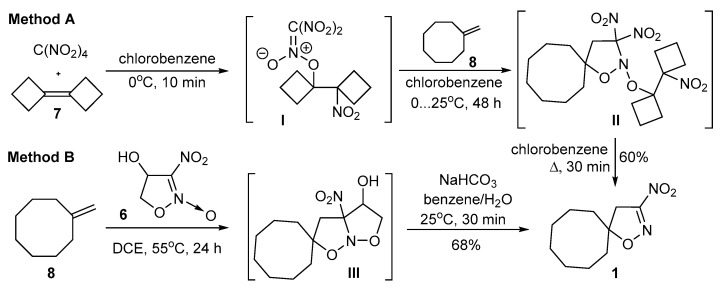
Synthesis of 3-nitroisoxazoline **1** employing 1,3-dipolar cycloaddition.

The most general method for the synthesis of isoxazolines is the 1,3-dipolar cycloaddition of nitrile oxides to alkenes [15]. However, this method is not applicable to 3-nitroisoxazolines since nitronitrile oxide is not available. Previously, in our laboratory, a preparative method of synthesis of 3,3-dinitroisoxazolidines and 3-nitroisoxazolines, employing heterocyclization of non-activated alkenes upon the treatment with tetranitromethane and bicyclobutylidene, was elaborated [16]. *N*-Oxide **6** (Scheme 1) was also described as a powerful and non-destructive agent for heterocyclization [17,18].

Methylidenecyclooctane (**8**) was involved in the reaction of 1,3-dipolar cycloaddition with alkylnitronate **I**, generated from tetranitromethane and bicyclobutylidene (Scheme 1). Subsequent decomposition of adduct **II** afforded spirocyclic 3-nitroisoxazoline **1** in good yield. Cycloaddition of *N*-oxide **6** to methylidenecyclooctane followed by work-up with NaHCO_3_ also afforded 3-nitroisoxazoline **1**, the yield being slightly higher.

**Scheme 2:**
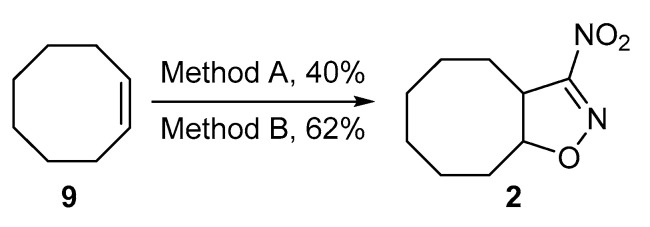
Synthesis of 3-nitroisoxazoline **2** employing 1,3-dipolar cycloaddition.

To obtain 3-nitroisoxazoline **2,** two methods of heterocyclization of cyclooctene were used. The target compound **2** was formed in both conditions, the use of *N*-oxide **6** being preferable (Scheme 2).

**Scheme 3:**
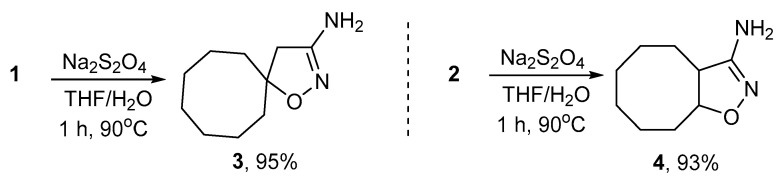
Synthesis of 3-aminoisoxazolines **3,4** via reduction of nitro-compounds.

3-Nitroisoxazolines **1** and **2** were reduced into corresponding 3-aminoisoxazolines **3,4** (Scheme 3). Taking into account the lability of the isoxazoline cycle in reductive conditions, as well as in presence of strong acids and bases, we chose sodium dithionite as a mild reductive agent, which has been previously used to obtain aminoisoxazoles [19]. Reduction of heterocycles **1** and **2,** upon treatment with sodium dithionite in THF-water mixture, allowed 3-aminoisoxazolines **3,4** in high yields.

Next, sulfonylation of 3-aminoisoxazoline **3** upon treatment with sulfonyl chlorides was investigated, with the aim of obtaining a series of cyclooctane-containing sulfonamides (Table 1). A brief optimization of the reaction conditions was made on the example of methanesulfonyl chloride. Variation of base (pyridine, diisopropyl(ethyl)amine (DIPEA)), reagents ratio and reaction time showed that the best yield of **5a** was achieved while using DIPEA and reagents ratio **3**/DIPEA/sulfonyl chloride 1:2:0.9; reaction was completed at 3 h. An excess of amine **3** was necessary to prevent the formation of the products of two-fold sulfonylation.

In optimal conditions, 3-aminoisoxazoline **3** was studied in reactions with sulfonyl chlorides bearing aryl and hetaryl substituents (Table 1). Generally, sulfonamides **5a-l** were obtained in moderate-to-good yields. In the case of benzylsulfonyl chloride, the product of two-fold sulfonylation was also formed in a significant amount, reducing the yield of **5b**. Low yields of compounds **5f,g** were connected to the difficulties during isolation via the column chromatography. The structure of the obtained compounds was proved *via* NMR spectroscopy employing 2D techniques when necessary (see Supplementary Materials).

### 2.2. Bioscreening Results and Molecular Modeling

To identify pharmacologically interesting agents from a library of new isoxazoline derivatives, we performed their preliminary screening for antiviral, antibacterial, antifungal and antiproliferative activity in vitro.

The antiviral activity of 3-aminoisoxazolines **3**,**4** against influenza A/Puerto Rico/8/34 (H1N1) virus was evaluated according to the procedure described in [20]. As can be seen from Table 2, the activity of both compounds **3,4** was in low to mid-micromolar concentrations, which is lower than that of the reference drug Rimantadine and lead compound **A**. Importantly, both 3-aminoisoxazolines **3**,**4** demonstrated low cytotoxicity to MDCK cells, resulting in high values of selectivity index.

The structural resemblance of basic moieties in spirothiazamenthane **A** and compound **3** allowed us to propose that antiviral activity of the latter is associated with action on the same target. The molecular target of compound **A** and reference drug Rimantadine is the influenza A M2 proton channel of viruses susceptible to Rimantadine, bearing serine at position 31. The virus used in the study, A/Puerto Rico/8/34 (H1N1), is resistant to Rimantadine due to the presence of N31 in the Rimantadine binding site.

**Figure 2:**
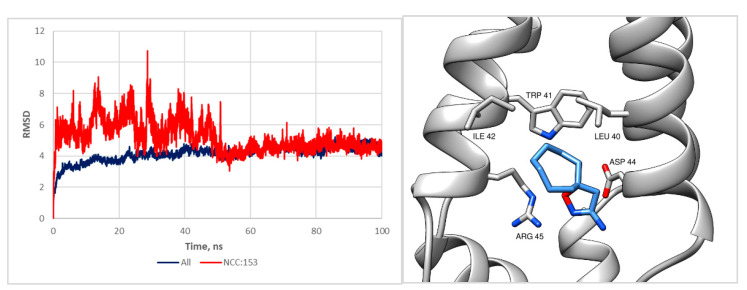
(**Left**): Mass-weighted root mean square deviations of the heavy atoms during the molecular dynamics simulation of the compound **3**/protein/membrane/water/ions system. (**Right**): Structure of the M2 channel (S31N mutant influenza virus, PDB: 2KIH) complex with compound **3** (in blue) obtained after the molecular dynamics simulation (two subunits of the channel, the lipid molecules and hydrogen atoms are omitted for clarity).

Molecular dynamics simulations, conducted using the procedure described in [21], indicate (Figure 2), that compound **3** is located in the transmembrane domain of the M2 proton channel of the S31N mutant influenza virus, in the region of amino acids 45–49 of different chains of the M2 tetramer, and does not have direct interactions with the pharmacologically relevant binding site of Rimantadine in the region of amino acid 31 [22]. This may explain high activity of compound **3** against the Rimantadine-resistant strain of the virus; however additional studies are needed, to establish the mechanism of the antiviral action of **3**.

It is worth mentioning that compound **A** itself is inactive against the Rimantadine-resistant M2 proton channel, but that such activity was evaluated for some of its derivatives [10]. This suggests promise in not only extended research of new compounds **3** and **4**, but also their modifications and the tests of their derivatives towards amantadine-resistant mutants.

Screening of antibacterial and antifungal activity, and of cyctotoxicity, was performed for the obtained cyclooctane-containing isoxazolines **1,2,3,4,5a-l**.

Evaluation of the antibacterial and antifungal properties has shown that only 3-nitroisoxazolines **1**,**2** had moderate antibiotic activity against the studied fungal cultures, as well as against the gram-positive bacteria (*Bacillus subtilis* и *Staphylococcus aureus*) (Table 3). Spirocyclic 3-nitroisoxazoline **1** was more active against fungal cultures, while its bicyclic analogue **2** displayed similar activity against fungi and bacteria.

The screening of anticancer activity showed that cyclooctane-containing isoxazoline derivatives generally possess low cyctotoxicity (Table 4).

Among the studied heterocycles, isoxazolines **1**, **2**, **5g,j** showed the best activity (IC_50_ 17.7–58.8 µM) against cancer cell lines, and the most vulnerable was colon cancer cells HCT-116. It should be noted that 3-aminoisoxazoles **3**,**4** were found to be not toxic against either cancer or normal cell lines.

## 3. Materials and Methods

### 3.1. Chemistry

#### 3.1.1. General Remarks

^1^H and ^13^C NMR spectra were recorded on a 400 MHz spectrometer Agilent 400-MR (400.0, 100.6 and 376.3 MHz for ^1^H, ^13^C and ^19^F, respectively) at r.t. in CDCl_3_, if not stated otherwise; chemical shifts δ were measured with reference to the solvent (CDCl_3_, δ_H_ = 7.26 ppm, δ_C_ = 77.16 ppm) or to CFCl_3_. When necessary, assignments of signals in NMR spectra were made using 2D techniques. Accurate mass measurements (HRMS) were obtained on a Jeol GCMate II mass spectrometer with electrospray ionization (ESI). Analytical thin layer chromatography was carried out with silica gel plates (supported on aluminum); the detection was done by UV lamp (254 and 365 nm). Column chromatography was performed on silica gel (Merck, 230–400 mesh). Alkene **9** [23], tetranitromethane [24], and *N*-oxide **6** [17] were obtained via the described methods. All other starting materials were commercially available. All reagents except commercial products of satisfactory quality were purified according to the literature procedures prior to use.

#### 3.1.2. One-Pot Synthesis of 3-Nitroisoxazolines 1,2

##### General Method A

To the solution of bicyclobutylidene (0.54 g, 5 mmol) in chlorobenzene (2 mL) a solution of tetranitromethane (0.46 mL, 0.98 g, 5 mmol) in chlorobenzene (8 mL) was added dropwise under stirring at 0 °C. Then, a solution of corresponding alkene (5 mmol) in chlorobenzene (1 mL) was added and the resulted mixture was stirred for 20 min at 0–5 °C and for 48 h at r.t. The reaction mixture was refluxed for 30 min. The solvent was evaporated under reduced pressure; the product was isolated via preparative column chromatography (SiO_2_).

##### General Method B

A mixture of *N*-oxide **5** (0.25 g, 1.67 mmol) and corresponding alkene (5 mmol) in DCM (5 mL) was stirred at 55 °C for 24 h. The solvent was evaporated under reduced pressure. Distilled water (5 mL), benzene (5 mL) and NaHCO_3_ (50 mg, 0.6 mmol) were added to the residue; the resulting mixture was stirred for 30 min at r.t. Then organic layer was separated, and water layer was extracted with benzene (3 × 3 mL). The combined organic layers were dried over MgSO_4_. The solvent was evaporated under reduced pressure; the product was isolated via preparative column chromatography (SiO_2_).

**3-Nitro-1-oxa-2-azaspiro[4.7]dodec-2-ene (1)**

Yield 0.63 g (2.97 mmol, 60%) via method A; 0.72 g (68%) via method B. Yellowish crystals, m.p. 55–57 °C. R*_f_* = 0.45 (light petrol:DCM = 1:1).

^1^H NMR (δ, ppm): 1.42–1.85 (m, 10H, 5CH_2_, cy-Oct), 1.85 (ddd, *J* 14.5, *J* 8.7, *J* 1.6, 2H, 2CH_2_, cy-Oct), 2.17 (ddd, *J* 14.5, *J* 10.0, *J* 1.6, 2H, 2CH_2_, cy-Oct), 3.18 (s, 2H, CH_2_, Isox); ^13^C NMR (δ, ppm): 21.7 (2CH_2_, cy-Oct), 24.2 (CH_2_, cy-Oct), 27.8 (2CH_2_, cy-Oct), 35.2 (2CH_2_, cy-Oct), 41.1 (CH_2_, Isox), 100.5 (C_spiro_), 162.3 (C-NO_2_).

HRMS (ESI^+^, *m*/*z*): calculated for C_10_H_16_N_2_O_3_ [M + Na]^+^: 235.1053, found: 235.1051.

**3-Nitro-3a,4,5,6,7,8,9,9a-octahydrocycloocta[d]isoxazole (2)**

Yield 0.39 г (1.97 mmol, 40%) via method A; 0.61 g (62%) via method B. Yellow crystals, m.p. 52–53 °C. R*_f_* = 0.38 (light petrol:DCM = 1:1).

^1^H NMR (δ, ppm): 1.15–1.29 (m, 1H, CH_2_, cy-Oct), 1.30–1.43 (m, 2H, 2CH_2_, cy-Oct), 1.44–1.57 (m, 1H, CH_2_, cy-Oct), 1.58–1.76 (m, 3H, 2CH_2_, cy-Oct), 1.77–1.87 (m, 3H, 2CH_2_, cy-Oct), 2.07–2.12 (m, 2H, CH_2_, cy-Oct), 3.47–3.68 (m, 1H, CH), 4.88–4.99 (m, 1H, CH-O); ^13^C NMR (δ, ppm): 23.9 (CH_2_, cy-Oct), 25.1 (CH_2_, cy-Oct), 25.31 (CH_2_, cy-Oct), 25.34 (CH_2_, cy-Oct), 25.7 (CH_2_, cy-Oct), 29.4 (CH_2_, cy-Oct), 46.3 (CH), 92.7 (CH-O), 167.6 (C-NO_2_).

HRMS (ESI^+^, *m*/*z*): calculated for C_9_H_14_N_2_O_3_ [M + Na]^+^: 221.0897, found: 221.0892.

#### 3.1.3. Synthesis of 3-Aminoisoxazolines 7,8 (General Method)

Na_2_S_2_O_4_ (1.04 g, 6 mmol) was added to the solution of corresponding 3-nitroisoxazoline (1 mmol) in THF-water mixture (1:1, 16 mL). The reaction mixture was stirred for 1 h at 90 °C and cooled down to r.t. Water (6 mL) and concentrated HCl (3 mL) were added and the resulting mixture was stirred for 15 мин at 60 °C. The mixture was cooled down to r.t., and solid NaHCO_3_ was added until the CO_2_ stopped evolving. Then, the reaction mixture was extracted with EtOAc (3 × 10 mL). The combined organic layers were dried over MgSO_4_. The solvent was evaporated under reduced pressure; the product was isolated via preparative column chromatography (SiO_2_).

**1-Oxa-2-azaspiro[4.7]dodec-2-en-3-amine (3)**

Yield 173 mg (0.95 mmol, 95%). White crystals, m.p. 192–193 °C (with decomposing). R*_f_* = 0.17 (light petrol:EtOAc = 1:4).

^1^H NMR (δ, ppm): 1.37–1.79 (m, 12H, 7CH_2_, cy-Oct), 1.98–2.11 (m, 2H, 2CH_2_, cy-Oct), 2.68 (s, 2H, CH_2_, Isox), 3.84 (br.s, 2H, NH_2_); ^13^C NMR (δ, ppm): 22.5 (2CH_2_, cy-Oct), 24.4 (CH_2_, cy-Oct), 28.1 (2CH_2_, cy-Oct), 34.6 (2CH_2_, cy-Oct), 46.2 (CH_2_, Isox), 88.8 (C_spiro_), 157.9 (C-NH_2_).

HRMS (ESI^+^, *m*/*z*): calculated for C_10_H_18_N_2_O [M + H]^+^: 183.1492, found: 183.1497.

**3a,4,5,6,7,8,9,9a-Octahydrocycloocta[d]isoxazol** **-3-amine (4)**

Yield 156 mg (0.92 mmol, 93%). White crystals, m.p. 186–187 °C (with decomposing). R*_f_* = 0.19 (light petrol:EtOAc = 1:4).

^1^H NMR (δ, ppm): 1.14–2.01 (m, 12H, 6CH_2_, cy-Oct), 2.82–2.90 (m, 1H, CH), 3.96 (br.s, 2H, NH_2_), 4.34–4.42 (m, 1H, CH-O); ^13^C NMR (δ, ppm): 24.6 (CH_2_, cy-Oct), 25.2 (CH_2_, cy-Oct), 25.4 (CH_2_, cy-Oct), 25.8 (CH_2_, cy-Oct), 25.9 (CH_2_, cy-Oct), 30.3 (CH_2_, cy-Oct), 51.6 (CH), 84.2 (CH-O), 162.6 (C-NH_2_).

HRMS (ESI^+^, *m*/*z*): calculated for C_9_H_16_N_2_O [M + H]^+^: 169.1335, found: 169.1333.

#### 3.1.4. Synthesis of Sulfonamides 5a-l (General Method)

The mixture of 3-aminoisoxazoline **3** (55 mg, 0.3 mmol) and DIPEA (104 μL, 0.6 mmol) in dry DCM (2 mL) was cooled down to 0 °C under argon, and corresponding sulfonyl chloride (0.27 mmol) was added. The reaction mixture was allowed to warm up to r.t. and stirred for 3 h, then it was worked up with water (5 mL), the organic layer was separated, and the water layer was extracted with DCM (3 × 5 mL). The combined organic layers were washed subsequently with saturated aqueous NaHCO_3_ (5 mL) and brine (5 mL) and dried over MgSO_4_. The solvent was evaporated under reduced pressure; the product was isolated via preparative column chromatography (SiO_2_).

***N*-(1-Oxa-2-azaspiro[4.7]dodec-2-en-3-yl)methanesulfonamide (5a)**

Yield 39 mg (0.15 mmol, 56%). White crystals, m.p. 110–111 °C. R*_f_* = 0.63 (light petrol:EtOAc = 5:1).

^1^H NMR (δ, ppm): 1.33–1.77 (m, 12H, 7CH_2_, cy-Oct), 2.04–2.16 (m, 2H, 2CH_2_, cy-Oct), 2.69 (s, 2H, CH_2_, Isox), 3.08 (s, 3H, CH_3_), 6.98 (br.s, 1H, NH); ^13^C NMR (δ, ppm): 22.0 (2CH_2_, cy-Oct), 24.9 (CH_2_, cy-Oct), 26.7 (CH_2_, Isox), 27.9 (2CH_2_, cy-Oct), 31.6 (2CH_2_, cy-Oct), 37.3 (CH_3_), 86.3 (C_spiro_), 117.4 (C-NH).

HRMS (ESI^+^, *m*/*z*): calculated for C_11_H_20_N_2_O_3_S [M + K]^+^: 299.0826, found: 299.0826.

***N*-(1-Oxa-2-azaspiro[4.7]dodec-2-en-3-yl)-1-phenylmethanesulfonamide (5b)**

Yield 30 mg (0.09 mmol, 33%); reaction time 3 days. Yellow crystals, m.p. 68–70 °C. R*_f_* = 0.25 (light petrol:EtOAc = 4:1).

^1^H NMR (δ, ppm): 1.29–1.80 (m, 12H, 7CH_2_, cy-Oct), 1.80–1.94 (m, 2H, 2CH_2_, cy-Oct), 2.43 (s, 2H, CH_2_, Isox), 4.47 (s, 2H, CH_2_-Ph), 7.33–7.47 (m, 5H, 5CH), 7.85 (br.s, 1H, NH); ^13^C NMR (δ, ppm): 22.2 (2CH_2_, cy-Oct), 24.4 (CH_2_, cy-Oct), 28.0 (2CH_2_, cy-Oct), 34.7 (2CH_2_, cy-Oct), 44.4 (CH_2_, Isox), 60.5 (CH_2_-Ph), 90.8 (C_spiro_), 117.2 (C-NH), 128.3 (C), 129.2 (2CH), 129.4 (CH), 131.4 (2CH).

HRMS (ESI^+^, *m*/*z*): calculated for C_17_H_24_N_2_O_3_S [M + Na]^+^: 359.1400, found: 359.1389.

***N*-(1-Oxa-2-azaspiro[4.7]dodec-2-en-3-yl)benzenesulfonamide (5c)**

Yield 77 mg (0.24 mmol, 88%). White crystals, m.p. 156–157 °C. R*_f_* = 0.10 (light petrol:EtOAc = 8:1).

^1^H NMR (δ, ppm): 1.28–1.76 (m, 12H, 7CH_2_, cy-Oct), 2.03–2.11 (m, 2H, 2CH_2_, cy-Oct), 2.71 (s, 2H, CH_2_, Isox), 6.79 (br.s, 1H, NH), 7.51–7.60 (m, 2H, 2CH), 7.62–7.69 (m, 1H, CH), 7.91–7.98 (m, 2H, 2CH); ^13^C NMR (δ, ppm): 22.1 (2CH_2_, cy-Oct), 24.9 (CH_2_, cy-Oct), 26.7 (CH_2_, Isox), 27.9 (2CH_2_, cy-Oct), 31.6 (2CH_2_, cy-Oct), 86.2 (C_spiro_), 117.5 (C-NH), 128.8 (2CH), 129.2 (2CH), 134.0 (CH), 136.5 (C-S).

HRMS (ESI^+^, *m*/*z*): calculated for C_16_H_22_N_2_O_3_S [M + Na]^+^: 345.1243, found: 345.1242.

**4-Methyl-*N*-(1-oxa-2-azaspiro[4.7]dodec-2-en-3-yl)benzenesulfonamide (5d)**

Yield 45 mg (0.13 mmol, 50%). White crystals, m.p. 122–124 °C. R*_f_* = 0.34 (light petrol:EtOAc = 5:1).

^1^H NMR (δ, ppm): 1.32–1.73 (m, 12H, 7CH_2_, cy-Oct), 2.01–2.12 (m, 2H, 2CH_2_, cy-Oct), 2.44 (s, 3H, CH_3_), 2.70 (s, 2H, CH_2_, Isox), 6.72 (br.s, 1H, NH), 7.31–7.37 (m, 2H, 2CH), 7.79–7.84 (m, 2H, 2CH); ^13^C NMR (δ, ppm): 21.8 (CH_3_), 22.1 (2CH_2_, cy-Oct), 24.9 (CH_2_, cy-Oct), 26.7 (CH_2_, Isox), 28.0 (2CH_2_, cy-Oct), 31.6 (2CH_2_, cy-Oct), 86.1 (C_spiro_), 117.5 (C-NH), 128.9 (2CH), 129.9 (2CH), 133.5 (C-S), 145.1 (C-CH_3_).

HRMS (ESI^+^, *m*/*z*): calculated for C_17_H_24_N_2_O_3_S [M + H]^+^: 337.1580, found: 337.1578.

**2,4,6-Trimethyl-*N*-(1-oxa-2-azaspiro[4.7]dodec-2-en-3-yl)benzenesulfonamide (5e)**

Yield 46 mg (0.13 mmol, 47%); reaction time 30 min. White crystals, m.p. 135–136 °C. R*_f_* = 0.16 (light petrol:EtOAc = 8:1).

^1^H NMR (δ, ppm): 1.30–1.74 (m, 12H, 7CH_2_, cy-Oct), 1.90–2.02 (m, 2H, 2CH_2_, cy-Oct), 2.33 (s, 3H, CH_3_), 2.58 (s, 2H, CH_2_, Isox), 2.66 (s, 6H, 2CH_3_), 6.70 (br.s, 1H, NH), 7.00 (br.s, 2H, 2CH); ^13^C NMR (δ, ppm): 21.2 (CH_3_), 22.0 (2CH_2_, cy-Oct), 23.3 (2CH_3_), 24.9 (CH_2_, cy-Oct), 26.6 (CH_2_, Isox), 28.0 (2CH_2_, cy-Oct), 31.8 (2CH_2_, cy-Oct), 85.7 (C_spiro_), 117.2 (C-NH), 130.8 (C-S), 132.4 (2CH), 140.7 (2C-CH_3_), 143.9 (C-CH_3_).

HRMS (ESI^+^, *m*/*z*): calculated for C_19_H_28_N_2_O_3_S [M + H]^+^: 365.1893, found: 365.1888.

***N*-(1-Oxa-2-azaspiro[4.7]dodec-2-en-3-yl)-2,3-dihydrobenzo[b][1,4]dioxine-6-sulfonamide (5f)**

Yield 38 mg (0.10 mmol, 37%). White crystals, m.p. 192–194 °C. R*_f_* = 0.63 (light petrol:EtOAc = 1:1).

^1^H NMR (δ, ppm): 1.30–1.75 (m, 12H, 7CH_2_, cy-Oct), 2.00–2.14 (m, 2H, 2CH_2_, cy-Oct), 2.70 (s, 2H, CH_2_, Isox), 4.27–4.37 (m, 4H, 2CH_2_-O), 6.62 (br.s, 1H, NH), 6.98 (d, *J* 8.4, 1H, CH), 7.39–7.47 (m, 2H, 2CH); ^13^C NMR (δ, ppm): 22.1 (2CH_2_, cy-Oct), 25.0 (CH_2_, cy-Oct), 26.7 (CH_2_, Isox), 28.0 (2CH_2_, cy-Oct), 31.6 (2CH_2_, cy-Oct), 64.2 (CH_2_-O), 64.8 (CH_2_-O), 86.2 (C_spiro_), 117.5 (C-NH), 117.9 (CH), 118.4 (CH), 122.7 (CH), 128.7 (C-S), 143.7 (C-O), 148.7 (C-O).

HRMS (ESI^+^, *m*/*z*): calculated for C_18_H_24_N_2_O_5_S [M + H]^+^: 381.1479, found: 381.1476.

***N*-(4-(*N*-1-Oxa-2-azaspiro[4.7]dodec-2-en-3-ylsulfamoyl)phenyl)acetamide (5g)**

Yield 32 mg (0.08 mmol, 31%); reaction time 3 days. White crystals, m.p. 171–173 °C (with decomposing). R*_f_* = 0.54 (light petrol:EtOAc = 1:2).

^1^H NMR (CD_3_OD, δ, ppm): 1.38–1.74 (m, 12H, 7CH_2_, cy-Oct), 1.97–2.09 (m, 2H, 2CH_2_, cy-Oct), 2.17 (s, 3H, CH_3_), 2.81 (s, 2H, CH_2_, Isox), 7.73–7.79 (m, 2H, 2CH), 7.83–7.89 (m, 2H, 2CH); ^13^C NMR (CD_3_OD, δ, ppm): 23.0 (2CH_2_, cy-Oct), 24.1 (CH_3_), 25.9 (CH_2_, cy-Oct), 27.2 (CH_2_, Isox), 29.0 (2CH_2_, cy-Oct), 32.6 (2CH_2_, cy-Oct), 86.2 (C_spiro_), 119.1 (C-NH), 120.1 (2CH), 130.9 (2CH), 132.8 (C-S), 144.9 (C-N(CO)), 172.0 (C=O).

HRMS (ESI^+^, *m*/*z*): calculated for C_18_H_25_N_3_O_4_S [M + Na]^+^: 402.1458, found: 402.1448.

**2,4-Difluoro*-N*-(1-oxa-2-azaspiro[4.7]dodec-2-en-3-yl)benzenesulfonamide (5h)**

Yield 74 mg (0.21 mmol, 77%). White crystals, m.p. 118–119 °C. R*_f_* = 0.38 (light petrol:EtOAc = 4:1).

^1^H NMR (δ, ppm): 1.29–1.74 (m, 12H, 7CH_2_, cy-Oct), 1.96–2.09 (m, 2H, 2CH_2_, cy-Oct), 2.70 (s, 2H, CH_2_, Isox), 6.95–7.04 (m, 1H, CH + 1H, NH), 7.05–7.12 (m, 1H, CH), 7.87–8.02 (m, 1H, CH); ^13^C NMR (δ, ppm; *J*, Hz): 22.0 (2CH_2_, cy-Oct), 24.9 (CH_2_, cy-Oct), 26.8 (CH_2_, Isox), 27.9 (2CH_2_, cy-Oct), 31.5 (2CH_2_, cy-Oct), 86.6 (C_spiro_), 105.9 (dd, ^2^*J*_CF_ = ^2^*J*_CF_ = 26, CH), 112.6 (dd, ^2^*J*_CF_ = 22, ^4^*J*_CF_ = 3, CH), 117.1 (C-NH), 120.8 (dd, ^2^*J*_CF_ = 14, ^4^*J*_CF_ = 4, C-S), 134.4 (d, ^2^*J*_CF_ = 22, CH), 160.1 (dd, ^1^*J*_CF_ = 259, ^3^*J*_CF_ = 12, C-F), 166.9 (dd, ^1^*J*_CF_ = 260, ^3^*J*_CF_ = 13, C-F).

^19^F NMR (δ, ppm): -104.65-(-104.54) (m, 1F, CF), -97.92-(-97.82) (m, 1F, CF).

HRMS (ESI^+^, *m*/*z*): calculated for C_16_H_20_F_2_N_2_O_3_S [M + Na]^+^: 381.1055, found: 381.1046.

**2-Nitro*-N*-(1-oxa-2-azaspiro[4.7]dodec-2-en-3-yl)benzenesulfonamide (5i)**

Yield 69 mg (0.19 mmol, 70%). White crystals, m.p. 164–165 °C. R*_f_* = 0.15 (light petrol:DCM = 1:5).

^1^H NMR (δ, ppm): 1.33–1.77 (m, 12H, 7CH_2_, cy-Oct), 1.99–2.15 (m, 2H, 2CH_2_, cy-Oct), 2.75 (s, 2H, CH_2_, Isox), 7.70 (br.s, 1H, NH), 7.78–7.87 (m, 2H, 2CH, Ar), 7.87–7.94 (m, 1H, CH, Ar), 8.25–8.33 (m, 1H, CH, Ar); ^13^C NMR (δ, ppm): 22.0 (2CH_2_, cy-Oct), 24.9 (CH_2_, cy-Oct), 26.9 (CH_2_, Isox), 27.9 (2CH_2_, cy-Oct), 31.5 (2CH_2_, cy-Oct), 86.8 (C_spiro_), 117.3 (C, Isox), 125.6 (CH, Ar), 130.1 (C(SO_2_)), 133.1 (CH, Ar), 134.3 (CH, Ar), 135.1 (CH, Ar), 148.6 (C-NO_2_).

HRMS (ESI^+^, *m*/*z*): calculated for C_16_H_2__1_N_3_O_5_S [M + Na]^+^: 390.1094, found: 390.1086.

***N*-(1-Oxa-2-azaspiro[4.7]dodec-2-en-3-yl)naphthalene-2-sulfonamide (5j)**

Yield 81 mg (0.22 mmol, 81%). White crystals, m.p. 143–145 °C. R*_f_* = 0.23 (light petrol:EtOAc = 4:1).

^1^H NMR (δ, ppm): 1.28–1.76 (m, 12H, 7CH_2_, cy-Oct), 1.99–2.12 (m, 2H, 2CH_2_, cy-Oct), 2.72 (s, 2H, CH_2_, Isox), 7.02–7.10 (m, 1H, NH), 7.53–7.68 (m, 2H, 2CH), 7.85 (d, 1H, *J* 8.0, CH), 7.87 (dd, 1H, *J* 8.7, *J* 1.7, CH), 7.91 (d, 1H, *J* 8.7, CH), 7.95 (d, 1H, *J* 8.0, CH), 8.50 (br.s, 1H, CH); ^13^C NMR (δ, ppm): 22.1 (2CH_2_, cy-Oct), 24.9 (CH_2_, cy-Oct), 26.7 (CH_2_, Isox), 27.9 (2CH_2_, cy-Oct), 31.6 (2CH_2_, cy-Oct), 86.2 (C_spiro_), 117.6 (C-NH), 123.4 (CH), 127.7 (CH), 128.0 (CH), 129.3 (CH), 129.4 (CH), 129.6 (CH), 130.8 (CH), 132.1 (C), 133.3 (C-S), 135.5 (C).

HRMS (ESI^+^, *m*/*z*): calculated for C_20_H_24_N_2_O_3_S [M + Na]^+^: 395.1400, found: 395.1389.

***N*** **-(1-Oxa-2-azaspiro[4.7]dodec-2-en-3-yl** **)thiophene** **-2-sulfonamide (5k)**

Yield 47 mg (0.14 mmol, 53%); reaction time 2 days. White crystals, m.p. 149–151 °C. R*_f_* = 0.20 (light petrol:EtOAc = 4:1).

^1^H NMR (δ, ppm): 1.32–1.78 (m, 12H, 7CH_2_, cy-Oct), 2.06–2.15 (m, 2H, 2CH_2_, cy-Oct), 2.72 (s, 2H, CH_2_, Isox), 6.75 (br.s, 1H, NH), 7.17 (dd, *J* 5.0, *J* 3.8, 1H, CH), 7.74 (dd, *J* 5.0, *J* 1.4, 1H, CH), 7.17 (dd, *J* 3.8, *J* 1.4, 1H, CH); ^13^C NMR (δ, ppm): 22.1 (2CH_2_, cy-Oct), 25.0 (CH_2_, cy-Oct), 26.8 (CH_2_, Isox), 28.0 (2CH_2_, cy-Oct), 31.7 (2CH_2_, cy-Oct), 86.5 (C_spiro_), 117.3 (C-NH), 127.8 (CH), 134.5 (CH), 135.0 (CH), 136.3 (C-S).

HRMS (ESI^+^, *m*/*z*): calculated for C_14_H_20_N_2_O_3_S_2_ [M + H]^+^: 329.0988, found: 329.0986.

***N*** **-(1-Oxa-2-azaspiro[4.7]dodec-2-en-3-yl)pyridine** **-3-sulfonamide (5** **l)**

Yield 58 mg (0.18 mmol, 66%). White crystals, m.p. 145–146 °C. R*_f_* = 0.39 (light petrol:EtOAc = 1:2).

^1^H NMR (δ, ppm): 1.28–1.79 (m, 12H, 7CH_2_, cy-Oct), 2.01–2.14 (m, 2H, 2CH_2_, cy-Oct), 2.70 (s, 2H, CH_2_, Isox), 7.51 (ddd, *J* 8.1, *J* 4.9, *J* 0.7, 1H, CH), 8.02 (br.s, 1H, NH), 8.25 (ddd, *J* 8.1, *J* 2.2, *J* 1.7, 1H, CH), 8.82 (dd, *J* 4.9, *J* 1.7, 1H, CH), 8.97 (dd, *J* 2.2, *J* 0.7, 1H, CH); ^13^C NMR (δ, ppm): 22.1 (2CH_2_, cy-Oct), 25.0 (CH_2_, cy-Oct), 26.6 (CH_2_, Isox), 27.9 (2CH_2_, cy-Oct), 31.6 (2CH_2_, cy-Oct), 86.5 (C_spiro_), 117.4 (C-NH), 124.0 (CH), 133.6 (C-S), 137.0 (CH), 149.1 (CH), 154.1 (CH).

HRMS (ESI^+^, *m*/*z*): calculated for C_15_H_21_N_3_O_3_S [M + Na]^+^: 346.1196, found: 346.1196.

### 3.2. Pharmacological Assays

#### 3.2.1. Evaluation of Antiviral Activity against Influenza Virus

Antiviral activity was evaluated against influenza A/Puerto Rico/8/34 (H1N1) wild type (WT) virus according to the procedure described in [20].

#### 3.2.2. Screening of Antimicrobial Activity

Initial screening of antimicrobial activity was performed by using agar-diffusion assay against a number of microorganisms: fungi (*Aspergillus niger* INA 00760), yeast (*Candida albicans* CBS 8836), gram-positive bacteria (*Bacillus subtilis* ATCC 6633, *Staphylococcus aureus* ATCC 25923) and gram-negative bacteria (*Escherichia coli* ATCC 25922). For the bacterial strains, the agar-diffusion method on the Mueller–Hinton medium was performed using direct colony suspension, equivalent to a 0.5 McFarland standard as inoculate. For the fungal test-strains, the Sabouraud medium was used with 0.2% glucose, inoculated with direct colony suspension (10^6^ CFU/100 mL). The tested compounds were applied on Petri dishes on sterile paper disks (6 mm); 100 µg/disk inhibition zones were measured after 24 h incubation at 37 °C. As a positive control, standard antibiotic disks containing 30 µg vancomycin (for *B. subtilis*), 10 µg gentamicin (for *E. coli*), and 40 µg fluconazole (for *A. niger* and *C. albicans*) were used.

Minimum inhibitory concentrations (MIC) were determined for the selected active strains according to the Performance Standards for Antimicrobial Susceptibility Testing (CLSI). The *Staphylococcus aureus* ATCC 29213 strain was used for MIC measurements. Amphotericin B, clotrimazole, vancomycin and ampicillin were used as positive control.

#### 3.2.3. MTT-Assay

The cytotoxic activity resulting structures was determined in vitro by the MTT assay [25].

HCT-116 (colon carcinoma), MCF-7 (breast adenocarcinoma), A-549 (lung adenocarcinoma) human cancer cell lines, WI38 (cell line composed of fibroblasts—non-tumorigenic), and Hek 293t (derivative of human embryonic kidney 293 cells, which contains the SV40 T-antigen—non-tumorigenic) were cultured in DMEM (PanEco, Moscow, Russia) with Glutamine (PanEco, Moscow, Russia) and antibiotics (PanEco, Moscow, Russia) in CO_2_ (5%) at 37 °C.

The compounds were dissolved (20 mM) in DMSO and then added to the cell-culture medium at the required concentration with a maximum DMSO content of 0.5 *v/v*%. At these concentrations, DMSO has no effect on cell viability, as shown in control experiments. Cells were cultured in 96-well plates (7000 cells/well) and treated with various concentrations of the test compounds at 37 °C for 72 h. Then, cell viability was determined by using the MTT assay, which quantified the dehydrogenase activity. The cells were incubated at 37 °C for 50 min with a solution of MTT [3-(4,5-dimethyldiazol-2-yl)-2,5-diphenyl tetrazolium bromide] (10 mL, 5 mg × mL^−1^) (Sigma–Aldrich, St. Louis, MO, USA). The supernatant was discarded, and the cells were dissolved in DMSO. The optical density of the solution was measured at 570 nm with the use of a multiwall-plate reader (Anthos Zenyth 2000rt, Biochrom, Great Britain), and the percentage of surviving cells was calculated from the absorbance of untreated cells. Each experiment was repeated at least three times, and each concentration was tested in at least three replicates. Data were presented as a graph of the percentage of surviving cells versus the concentration of the test substances. The meanings of 50% inhibitory concentration (IC_50_) with standard deviation were calculated using GraphPad Prism Version 5.03 for Windows.

### 3.3. Molecular Modeling

For molecular dynamics simulations, the apo-M2 conductance domain structure (residues 23–60) (the lowest-energy conformer) was obtained from the Protein Data Bank for the S31N mutant influenza virus (PDB: 2KIH). The starting structure of the M2CD complex with compound **3** was obtained by means of molecular docking to the region of amino acids 25–50 (the ligand structure and M2 channel model were prepared as described in [21]) using the AutoDock Vina 1.1.2 software [26] (grid box 11.25 Å × 11.25 Å × 11.25 Å, grid center size x = −23.987 Å, y = 4.634 Å, z = −6.149 Å, exhaustiveness = 20), complexes with the best value of scoring function was selected).

Molecular dynamics simulations were performed as described in [21] using the CHARMM-GUI Web service [27,28,29,30]. For the analysis and visualization of the results, the cpptraj software [31] in the AmberTools 18 package [32] and UCSF Chimera software [26] were used.

## 4. Conclusions

The synthesis of new 3-nitro- and 3-aminoisoxazolines containing spiro-fused or 1,2-annelated cyclooctane fragments was achieved. The reaction of 3-aminoisoxazoline containing a spiro-fused cyclooctane fragment with sulfonyl chlorides yielded a representative series of previously unknown spiro-building sulfonamides. Preliminary screening of the compounds for antiviral, antibacterial, antifungal and antiproliferative activity in vitro revealed very high—in the low nanomolar range of concentrations—activity of 1-oxa-2-azaspiro[4.7]dodec-2-en-3-amine (**3**) and 3a,4,5,6,7,8,9,9a-octahydrocycloocta[d]isoxazol-3-amine (**4**) against influenza A/Puerto Rico/8/34 (H1N1) wild type virus. Further studies of the mechanism of the antiviral action of these substances, as well as their effect on resistant strains, are currently being carried out, and will be published in due course.

## Figures and Tables

**Table 1:** Synthesis of sulfonamides **5a-l**.

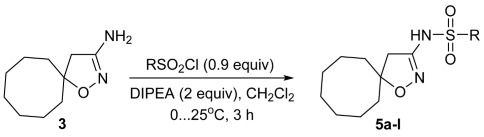					
Compound	R	Yield, % *	Compound	R	Yield, % *
**5a**	Me	56	**5g**	4-(MeC(O)NH)C_6_H_4_	31
**5b**	Bn	33	**5h**	2,4-F_2_C_6_H_3_	77
**5c**	Ph	88	**5i**	2-(NO_2_)C_6_H_4_	70
**5d**	4-MeC_6_H_4_	50	**5j**	3-naphthyl	81
**5e**	2,4,6-Me_3_C_6_H_2_	47	**5k**	2-thienyl	53
**5f**	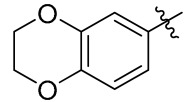	37	**5l**	3-pyridyl	66
	

* Isolated yield.

**Table 2:** Activity of 3-aminoisoxazolines **3,4** against WT A/Puerto Rico/8/34 (H1N1) virus.

Compound	IC_50_, μM *	CC_50_, μM **	SI ***
3	6.6	>550	83
4	22.0	>1790	81
Rimantadine	67 ± 8	406 ± 26	6

* IC_50_—half-maximal inhibitory concentration causing 50% inhibition of viral replication. ** CC_50_—half-maximal cytotoxic concentration causing the death of 50% cells. *** SI—selectivity index CC_50_/IC_50_.

**Table 3:** Antibacterial and antifungal activity of 3-nitroisoxazolines **1**,**2**.

Compound	MIK, μg/mL (Zone of Inhibition Ø, mm)				
	*A. niger*	*C. albicans*	*B. subtilis*	*St. aureus*	*E. coli*
**1**	8 (15)	8 (24)	32 (12)	32 (15)	˃256 (—)
**2**	16 (15)	32 (19)	32 (16)	8–16 (25)	˃256 (—)
Amphotericin B	0.04–1.5	2	—	—	—
Clotrimazole	—	4	—	—	—
Vancomycin	—	—	0.25–1	0.5–2	—
Ampicillin	—	—	—	—	2–8

**Table 4:** Cytotoxicity of isoxazoline derivatives.

Compound	IC_50_, µM *				
	MCF-7	HCT-116	A-549	WI38	Hek293t
**1**	58.8 ± 5	20.8 ± 5	57.1 ± 7	42.2 ± 13	n/a
**2**	35.4 ± 4.5	17.7 ± 5	40.4 ± 2.5	27.4 ± 4	n/a
**3**	>150	>500	>150	>150	n/a
**4**	>150	>500	>150	>150	n/a
**5b**	138.3 ± 25	>200	n/a	>500	n/a
**5c**	115.6 ± 30	63 ± 18	163	113.1	n/a
**5d**	94 ± 30	n/a	143 ± 50	54.6 ± 7	n/a
**5e**	79 ± 36	70.1 ± 11.2	61 ± 21	95.5 ± 14.3	n/a
**5f**	64.1 ± 8	23.6 ± 4.5	119	42 ± 8.6	n/a
**5g**	54.5 ± 15	41.3 ± 17	54 ± 14	n/a	30.36 ± 14.5
**5h**	94.7 ± 15.5	97.1 ± 34	77.1 ± 10.2	n/a	60 ± 17
**5j**	26.8 ± 3.5	19 ± 3	27.5 ± 4.2	n/a	23.1 ± 6.5
**5k**	>150	131 ± 80	111 ± 20	n/a	65.5 ± 13.5
**5l**	>150	133 ± 68	>150	n/a	>500

* Cancer cell lines: MCF-7 (breast cancer), HCT-116 (colon cancer), A-549 (lung cancer); normal cell lines: WI38 (fibroblasts), Hek293t (embryonic kidney).

## Data Availability

Not applicable.

## Supplementary Materials

The following supporting information can be downloaded at: https://www.mdpi.com/article/10.3390/molecules27113546/s1, copies of NMR spectra of the target compounds.

## Sample Availability

Samples of the compounds **1-4, 5a-l** are available from the authors.
