# Supplementary material for: Bicyclic Isoxazoline Derivatives: Synthesis and Evaluation of Biological Activity
Source: Molecules. 2022 May 31;27(11):3546. doi: 10.3390/molecules27113546 (PMC9182378; doi:10.3390/molecules27113546)

Bicyclic isoxazoline derivatives: synthesis and evaluation of biological activity

Kseniya N. Sedenkova [1], Kristian S. Andriasov [1], Marina G. Eremenko [1], Yuri K. Grishin [1], Vera A. Alferova[2,3], Anna A. Baranova[2,3], Nikolay A. Zefirov [1], Olga N. Zefirova [1], Vladimir V. Zarubaev [4], Yulia A. Gracheva [1], Elena R. Milaeva [1] and Elena B. Averina [1,*]

[1] Department of Chemistry, Lomonosov Moscow State University, Leninskie Gory 1-3, Moscow, Russia; elaver@med.chem.msu.ru
[2] Gause Institute of New Antibiotics, B. Pirogovskaya 11, 119021 Moscow, Russia; alferovava@gmail.com (V.A.A.); anjabaranowa@list.ru (A.A.B.)
[3] Shemyakin-Ovchinnikov Institute of Bioorganic Chemistry, Miklukho-Maklaya 16/10, 117997 Moscow, Russia
[4] Saint Petersburg Pasteur Research Institute of Epidemiology and Microbiology, 14 Mira St., Saint Petersburg 197101, Russia; zarubaev@gmail.com
* Correspondence: elaver@med.chem.msu.ru

Supporting information

Copies of NMR spectra……………………………………………………………….……..2

^{1}H NMR ($CDCl_3$) spectrum of compound **1**

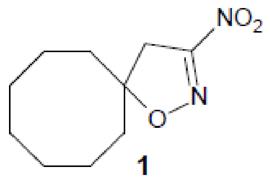

1

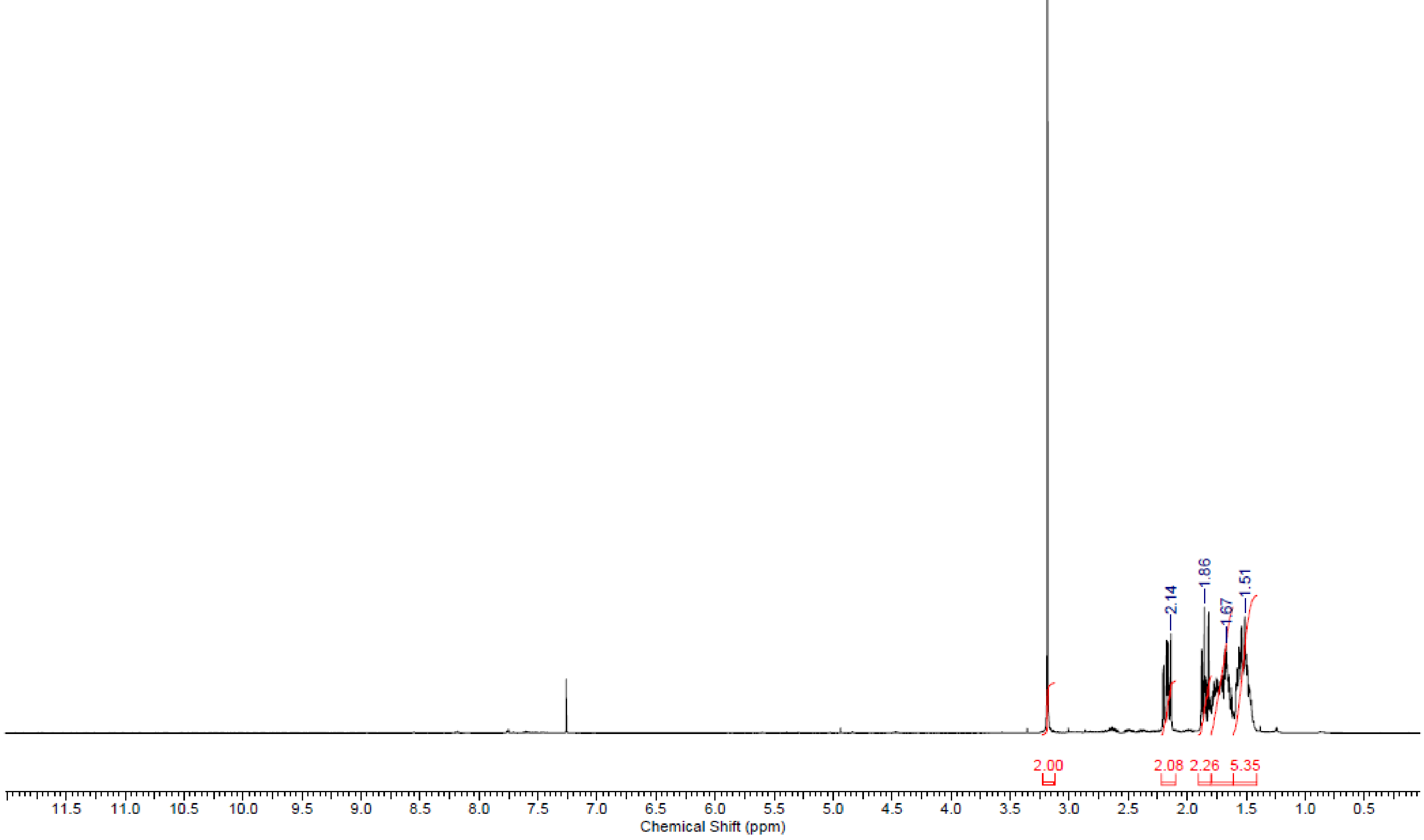

^{13}C NMR ($CDCl_3$) spectrum of compound **1**

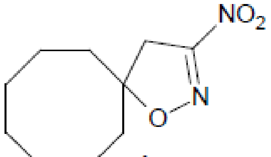

1

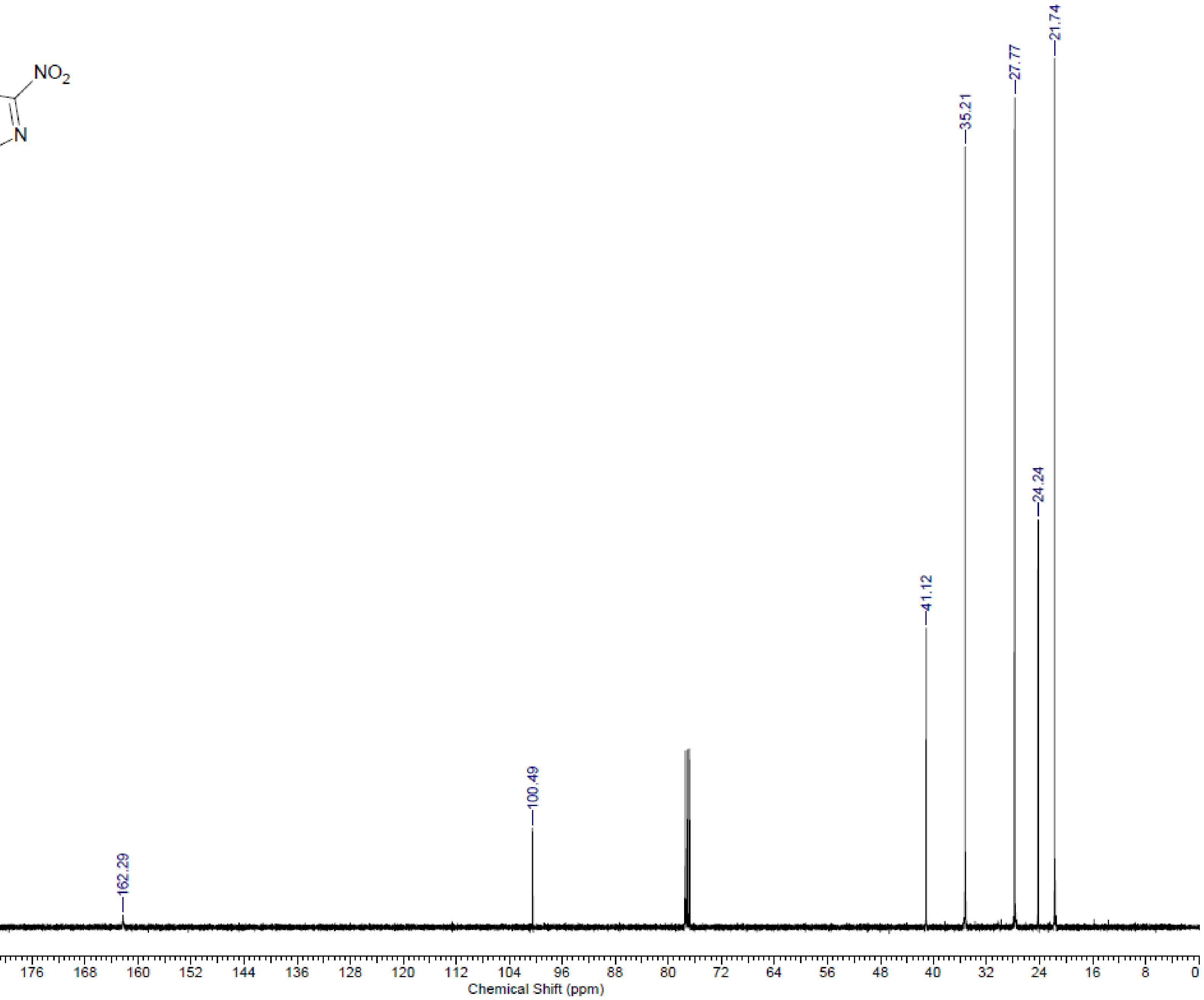

HSQC NMR ($CDCl_3$) spectrum of compound **1**

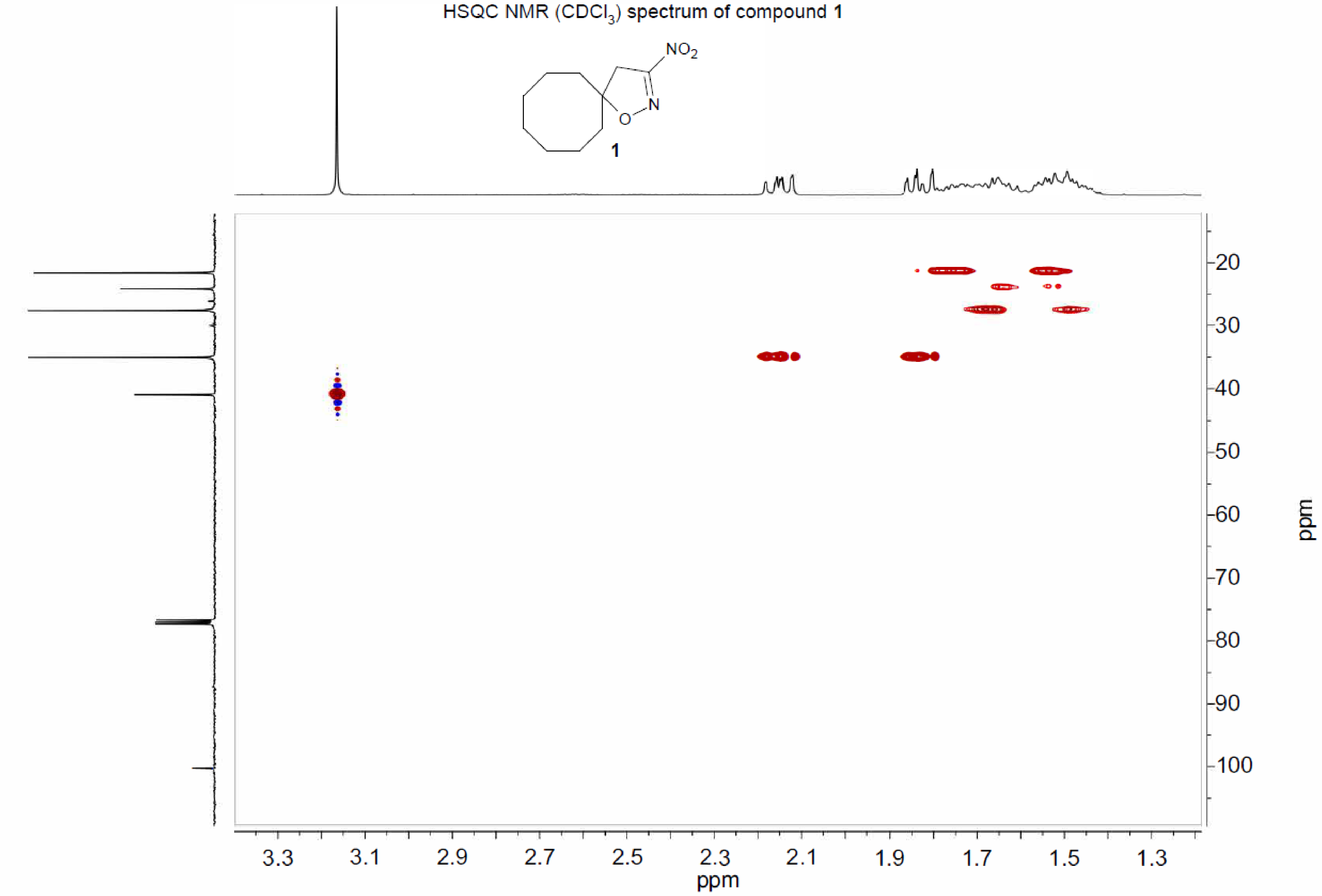

HMBC NMR ($CDCl_3$) spectrum of compound **1**

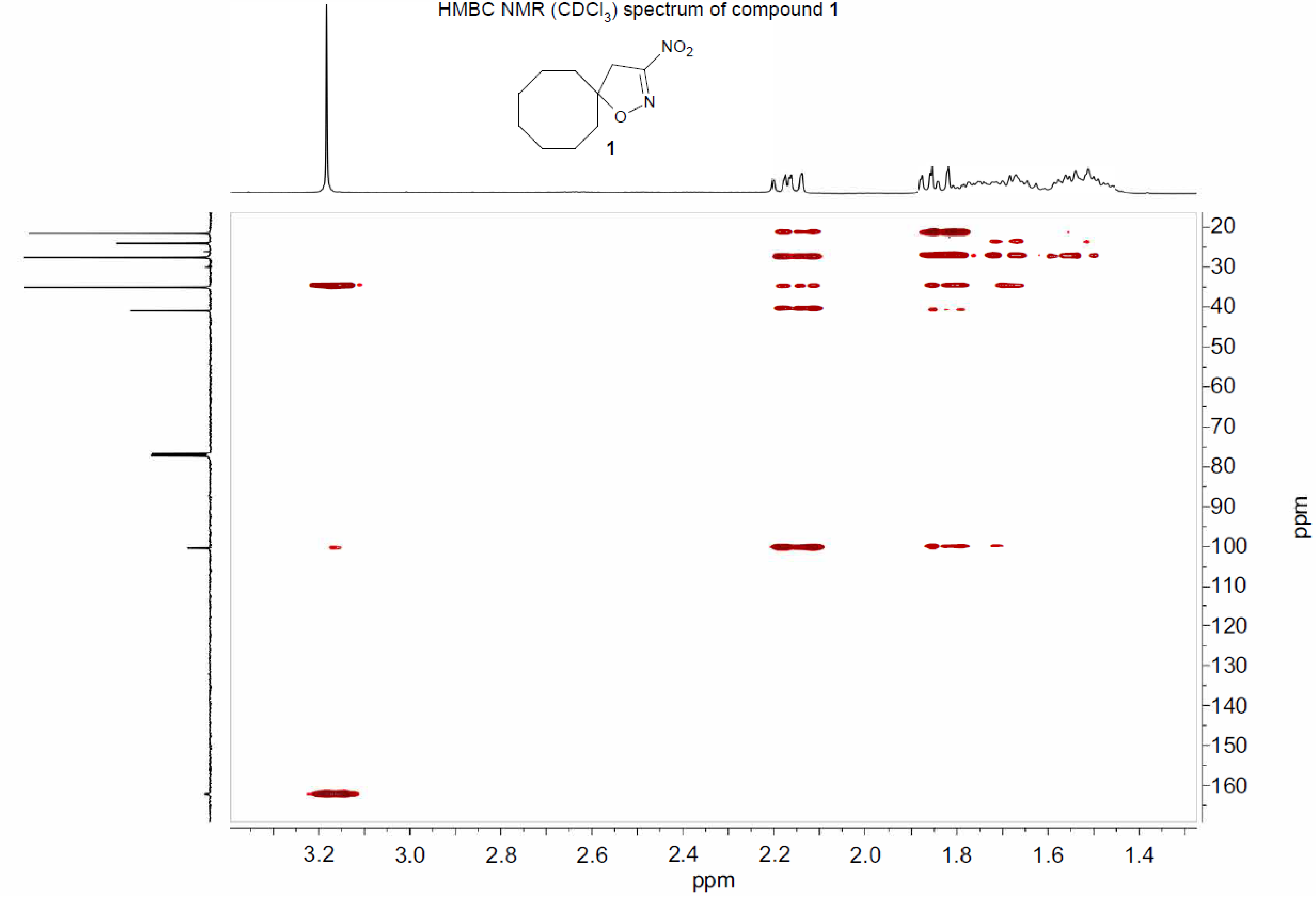

^{1}H NMR ($CDCl_3$) spectrum of compound **2**

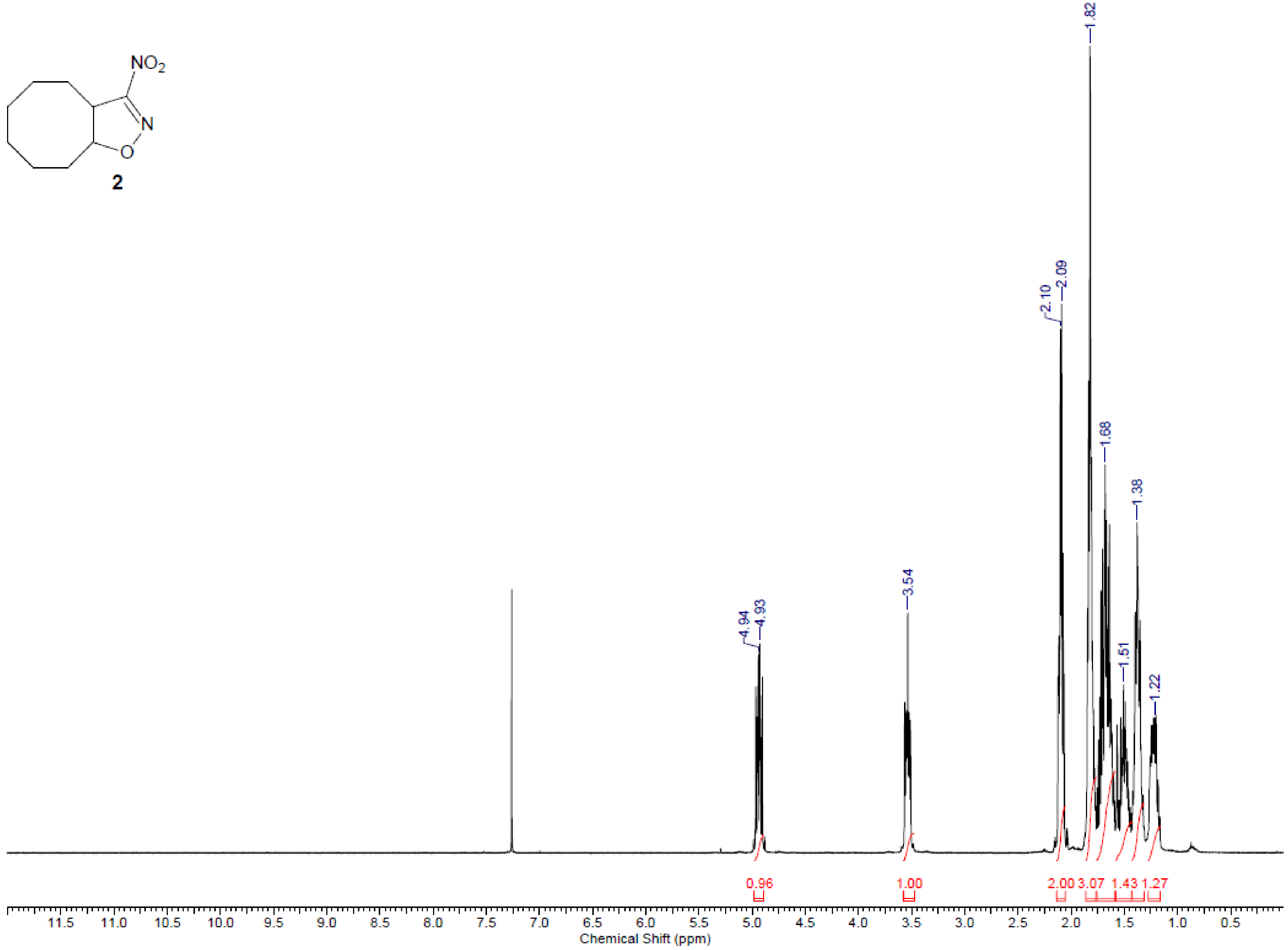

^{13}C NMR ($CDCl_3$) spectrum of compound **2**

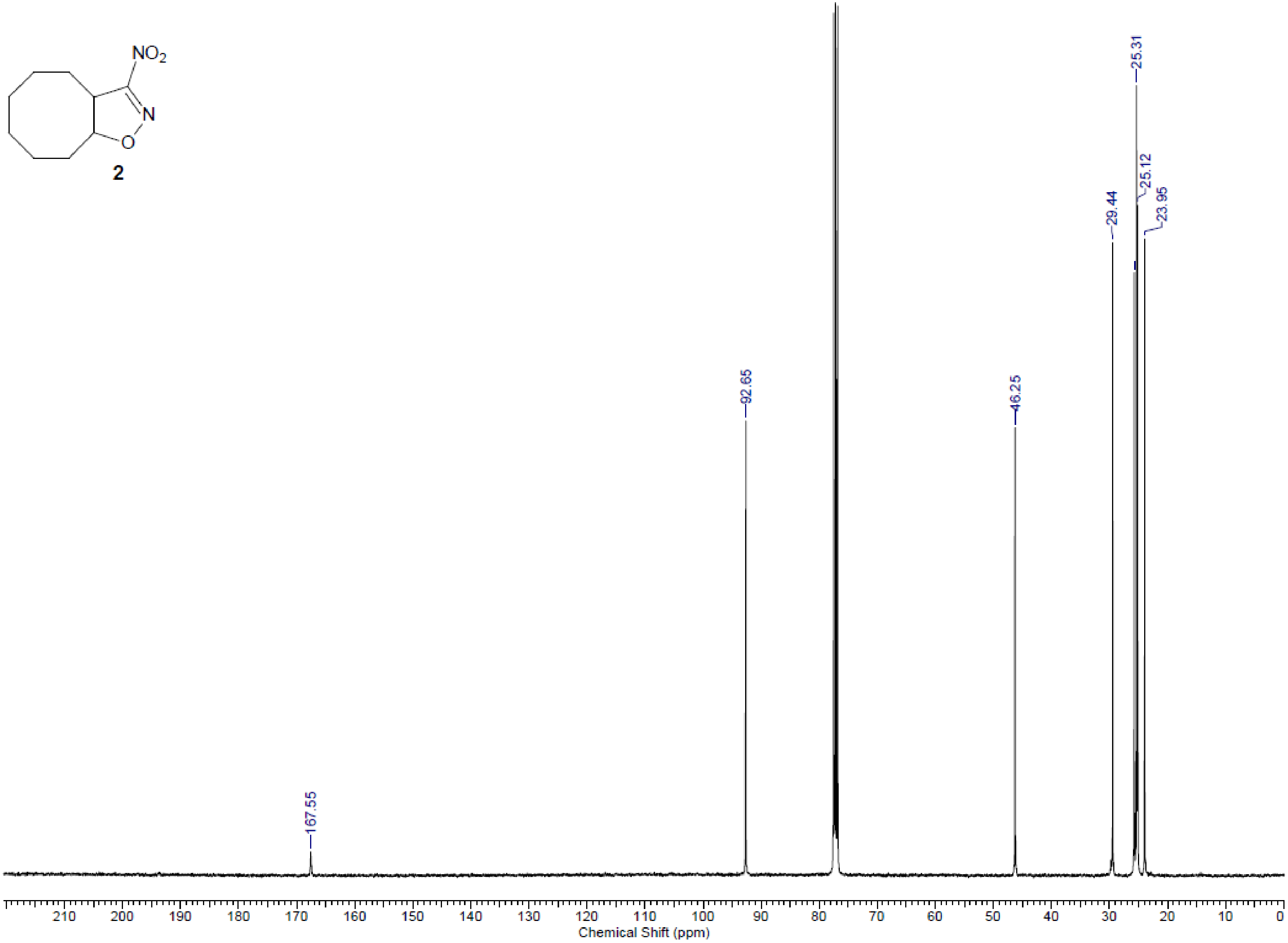

HSQC NMR ($CDCl_3$) spectrum of compound **2**

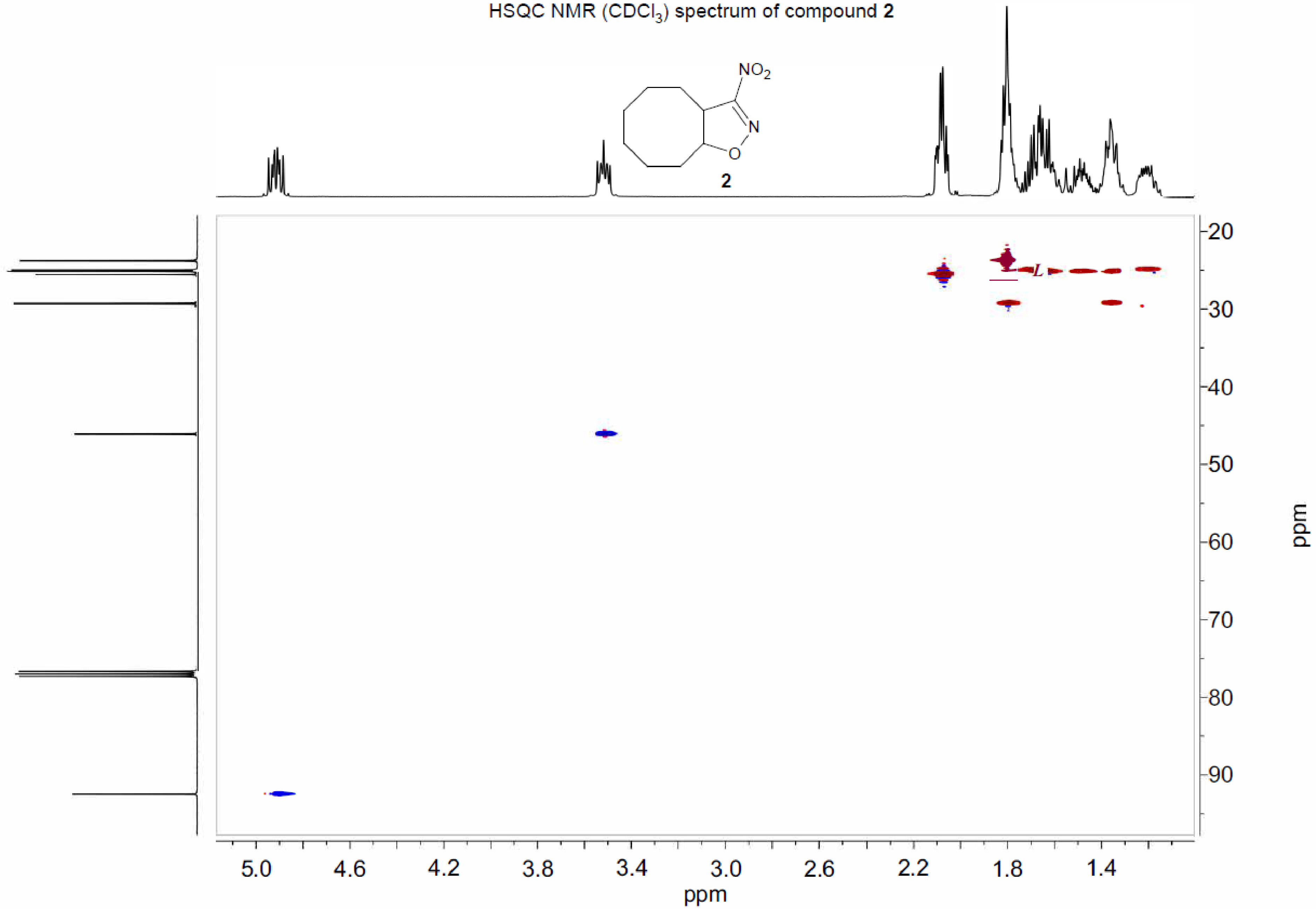

HMBC NMR ($CDCl_3$) spectrum of compound **2**

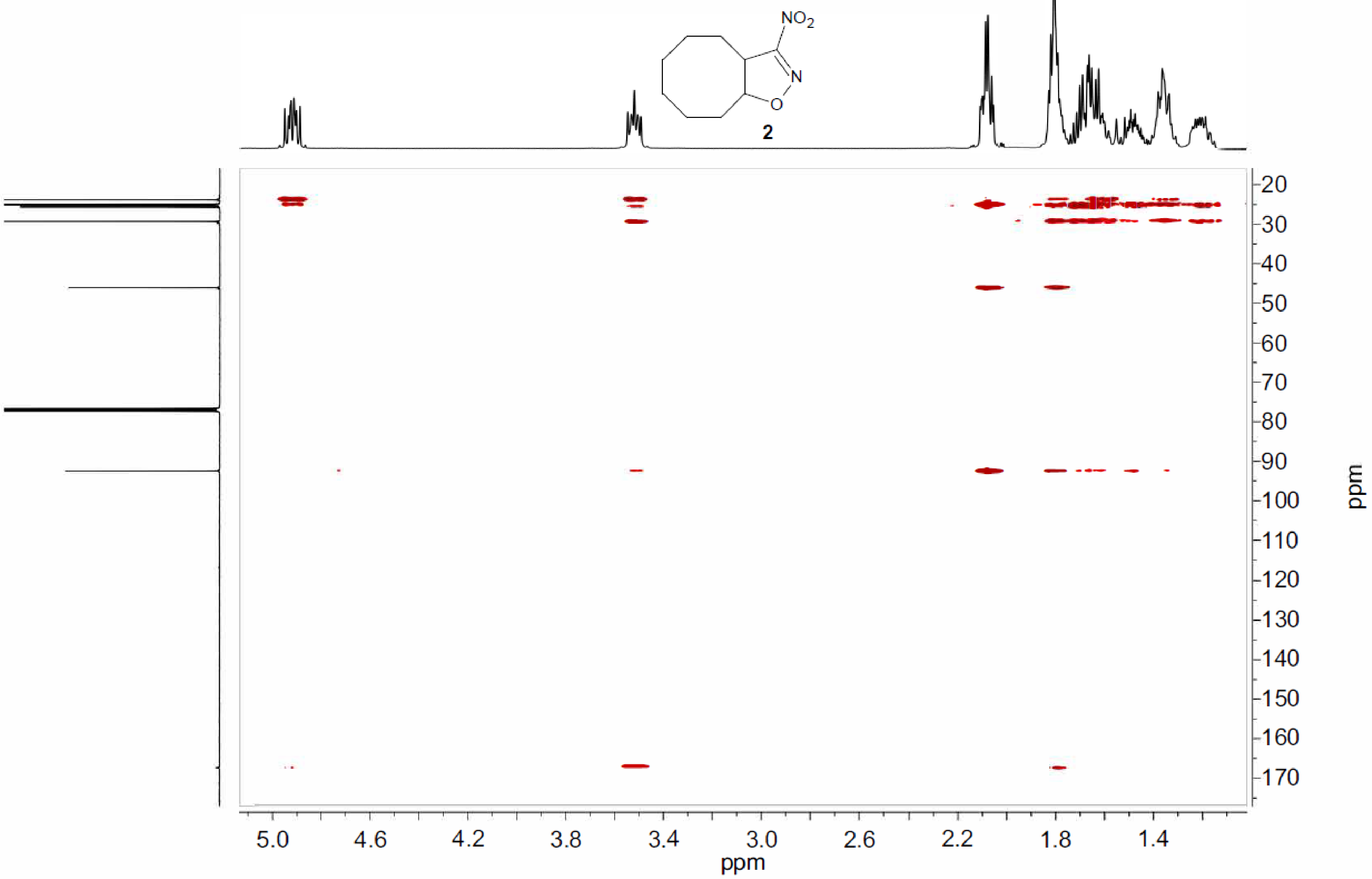

^{1}H NMR ($CDCl_3$) spectrum of compound **3**

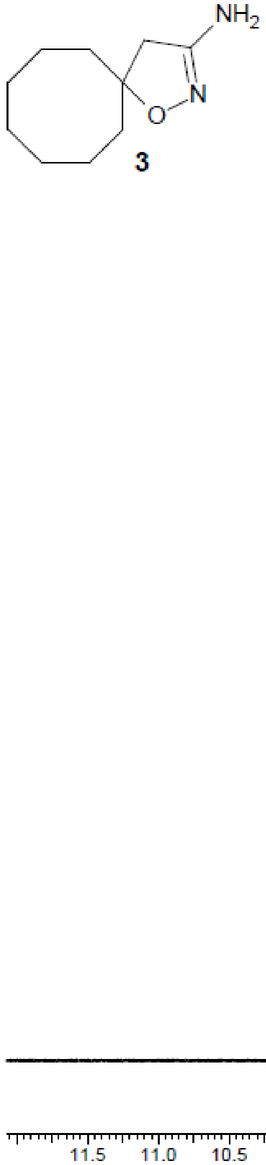

3

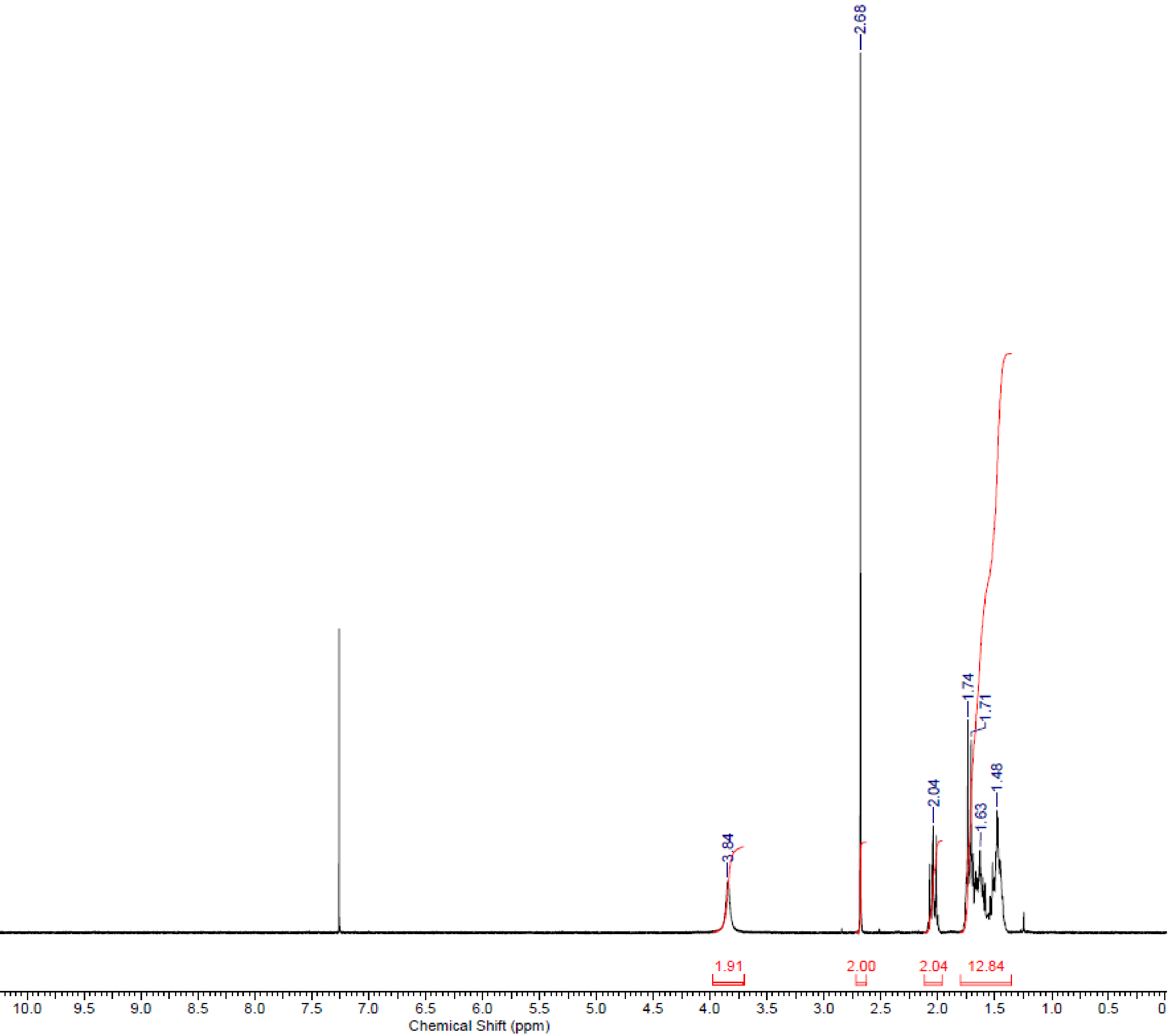

^{13}C NMR ($CDCl_3$) spectrum of compound **3**

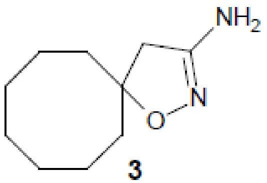

3

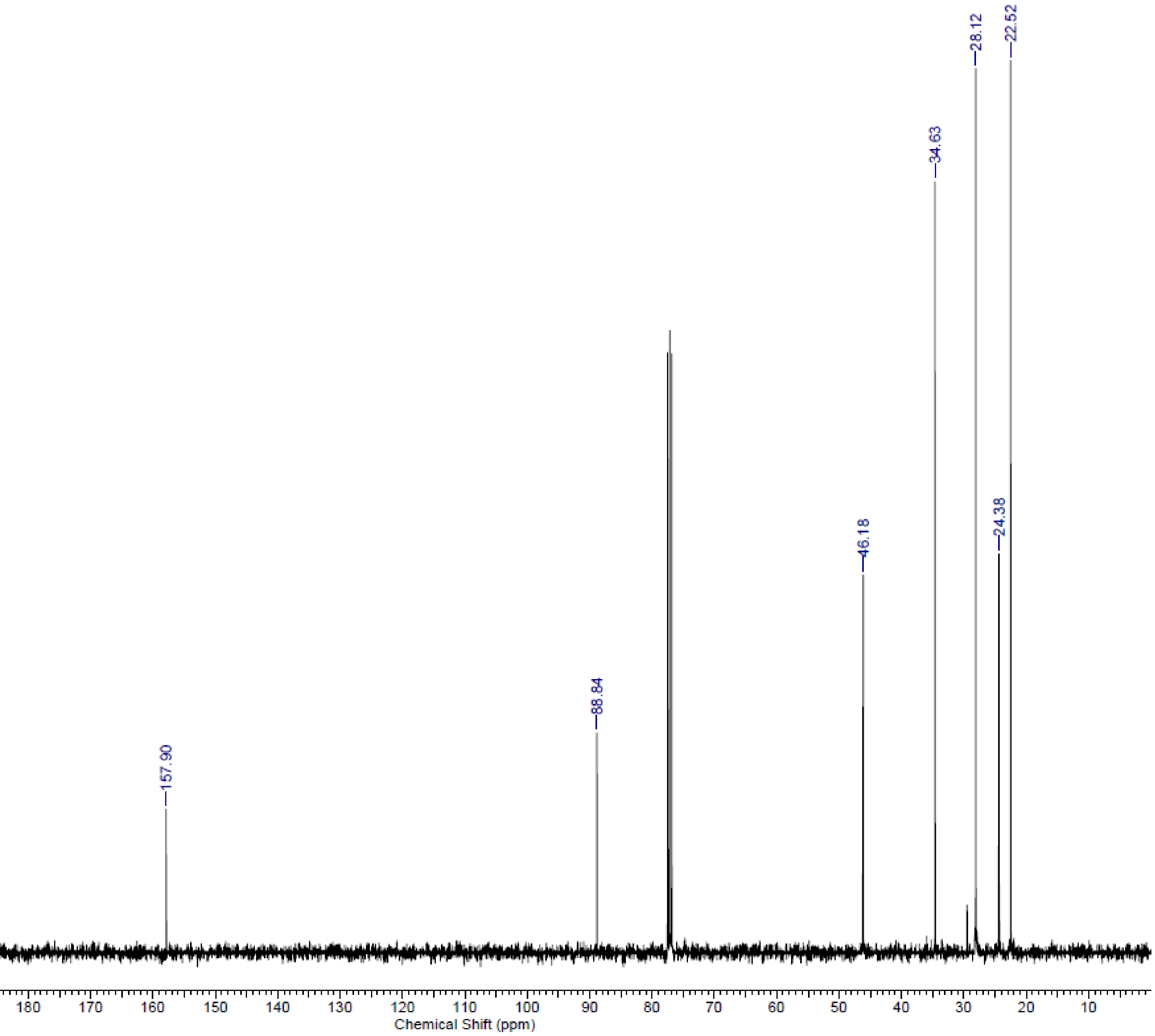

^{1}H NMR ($CDCl_3$) spectrum of compound **4**

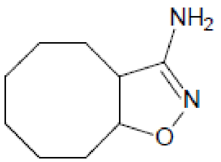

4

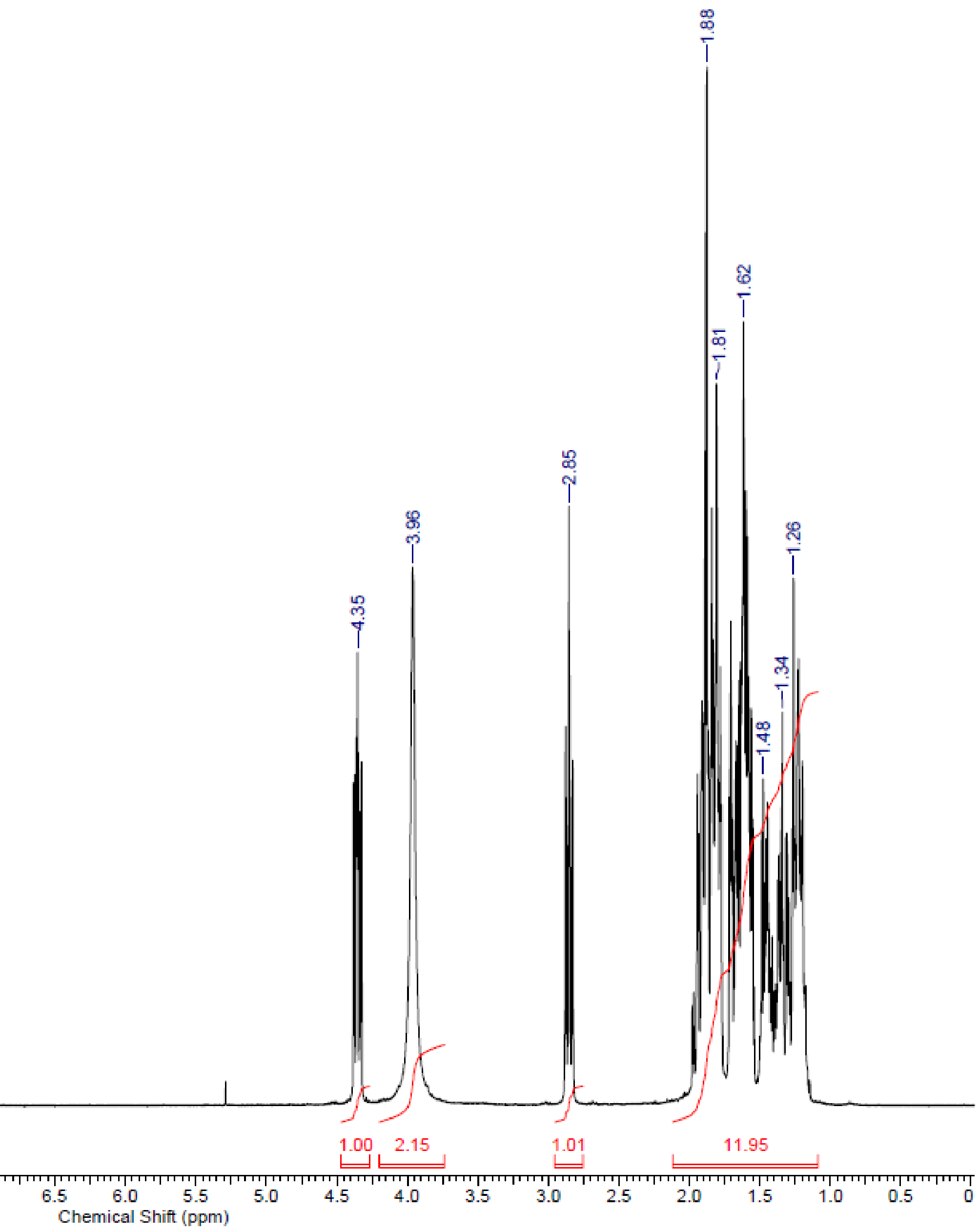

^{13}C NMR ($CDCl_3$) spectrum of compound **4**

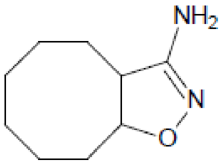

4

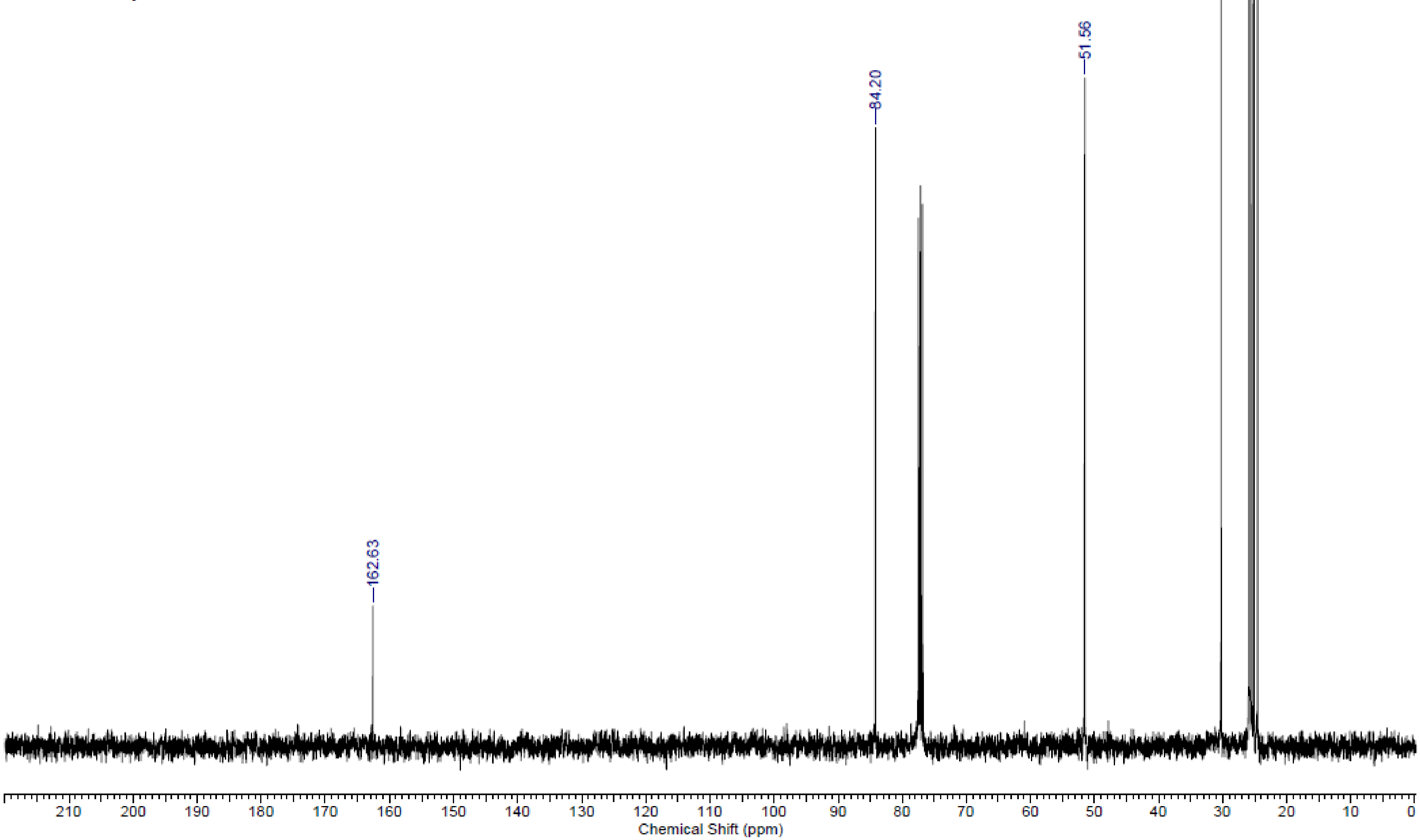

HSQC ($CDCl_3$) spectrum of compound **4**

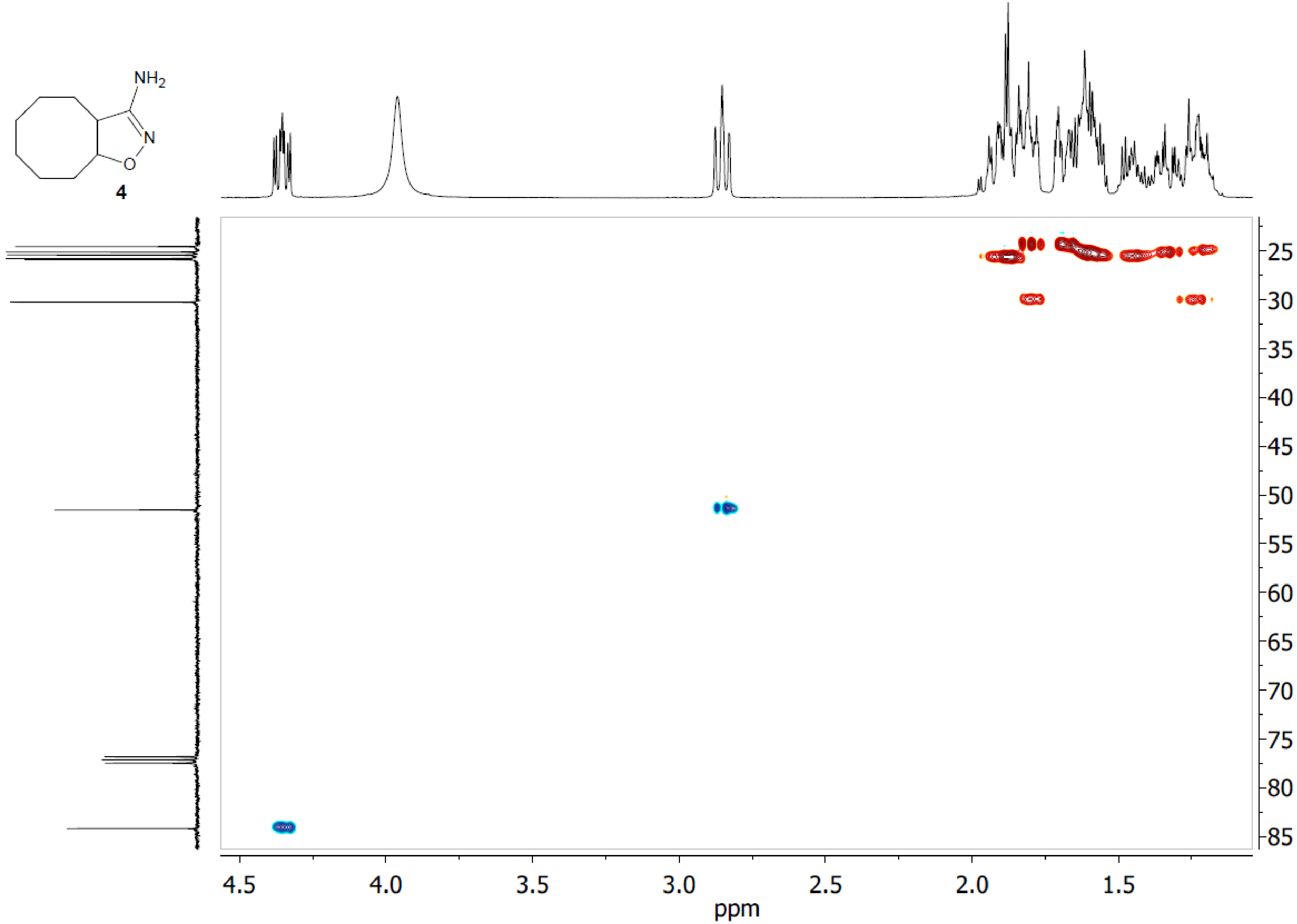

^{1}H NMR ($CDCl_3$) spectrum of compound **5a**

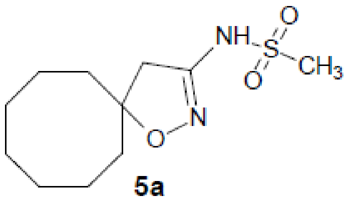

5a

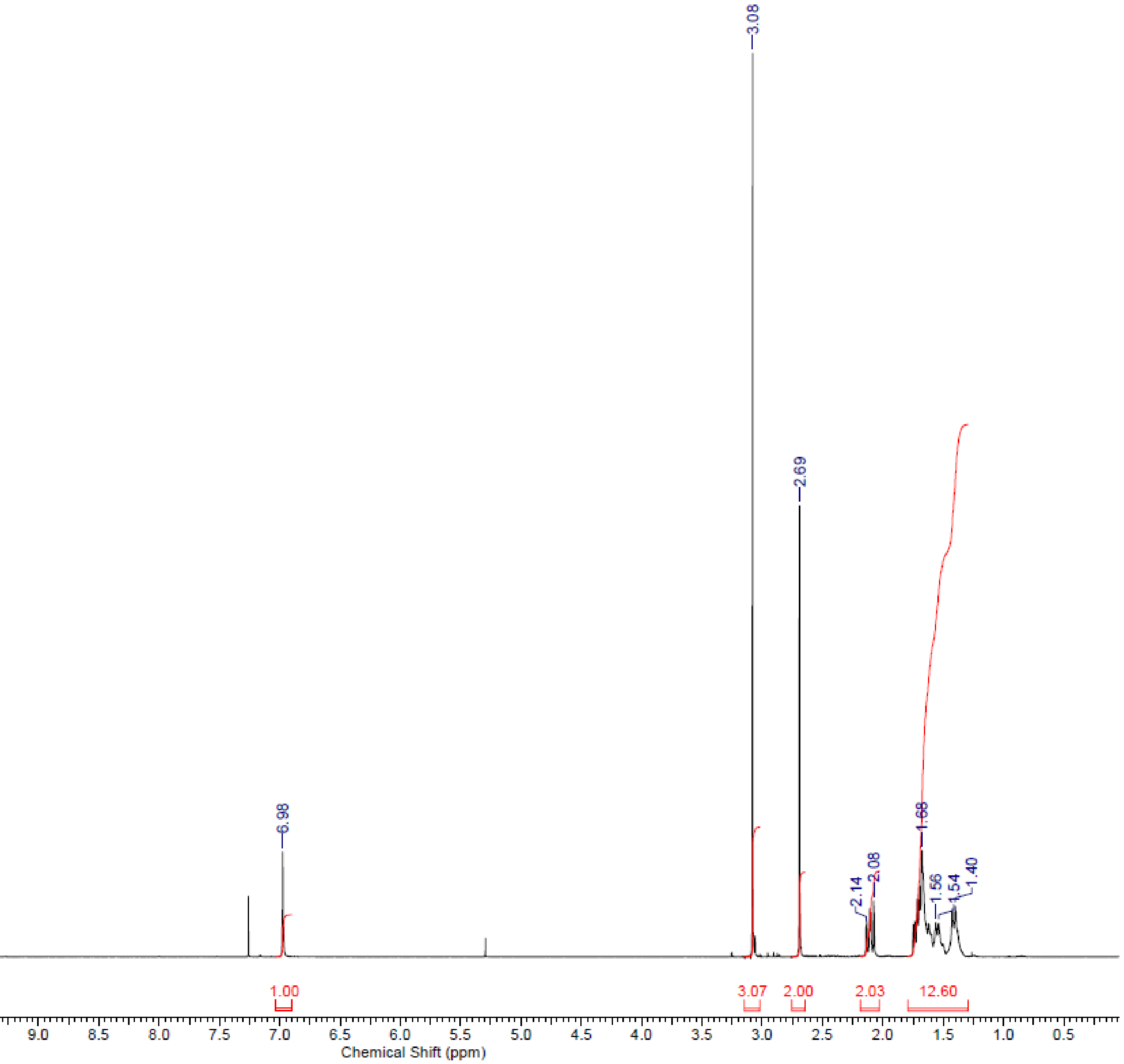

^{13}C NMR ($CDCl_3$) spectrum of compound **5a**

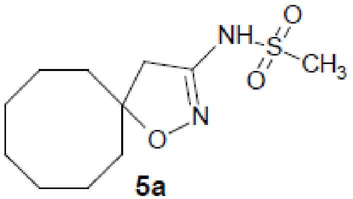

5a

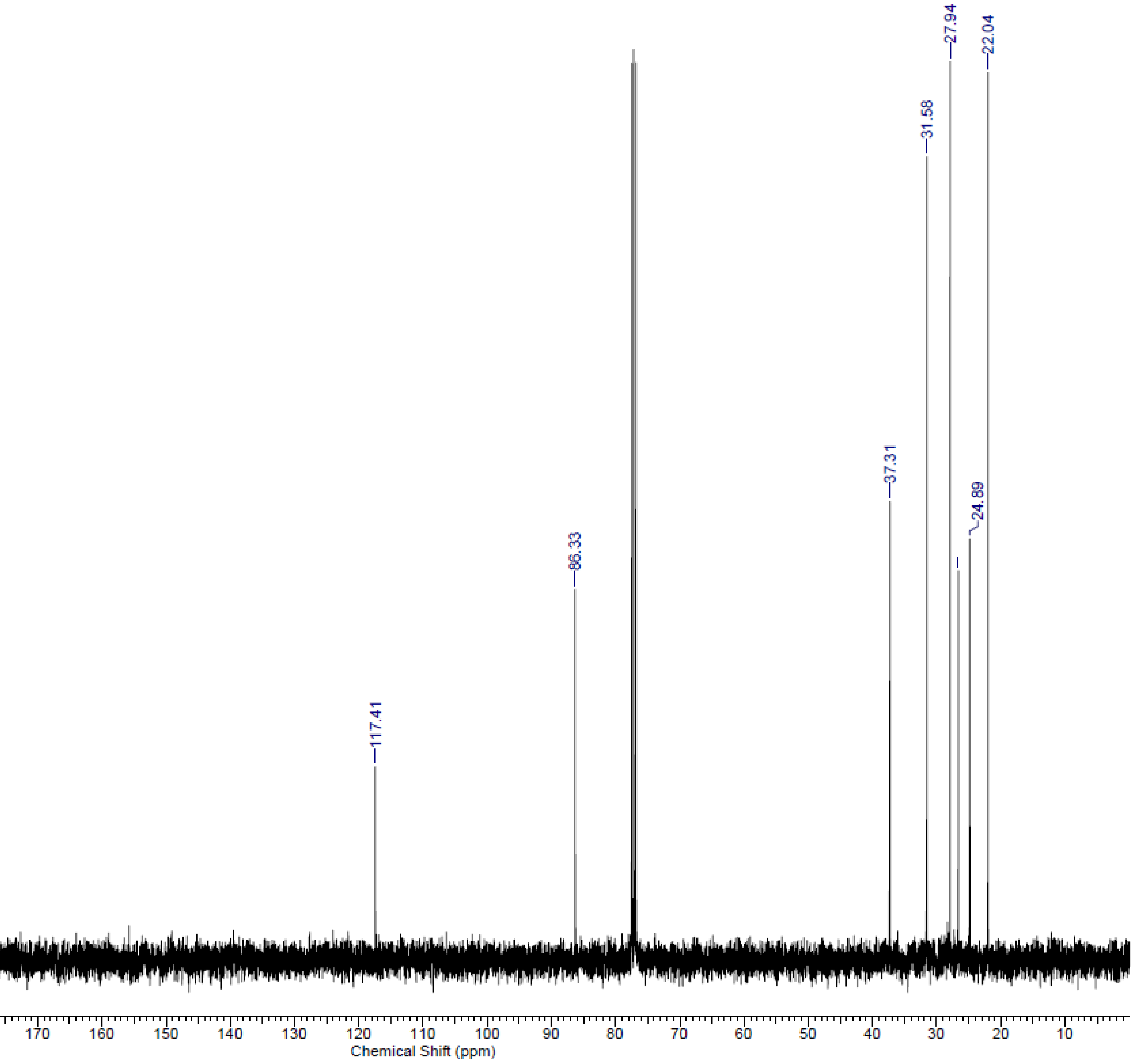

^{1}H NMR ($CDCl_3$) spectrum of compound **5b**

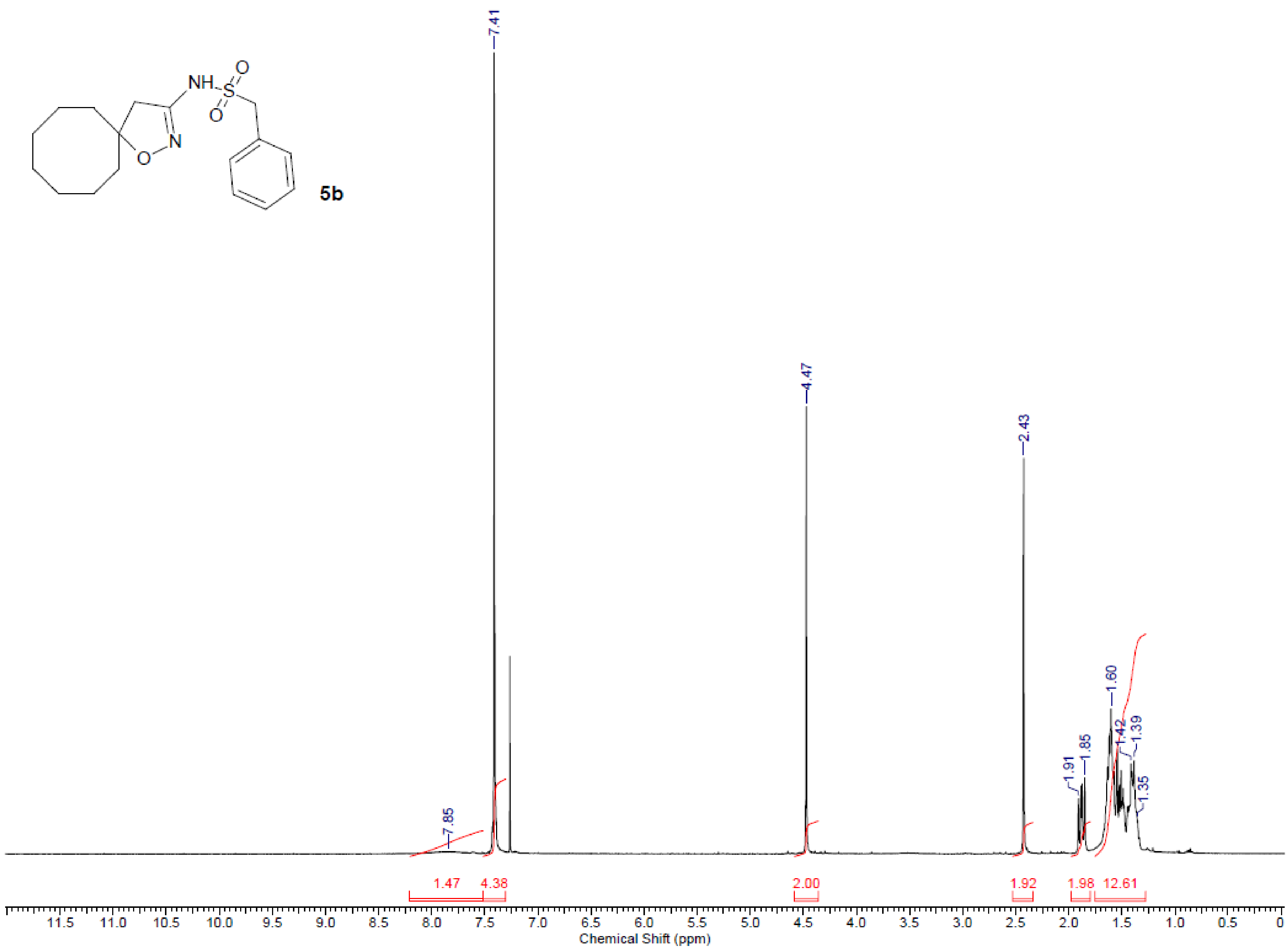

^{13}C NMR ($CDCl_3$) spectrum of compound **5b**

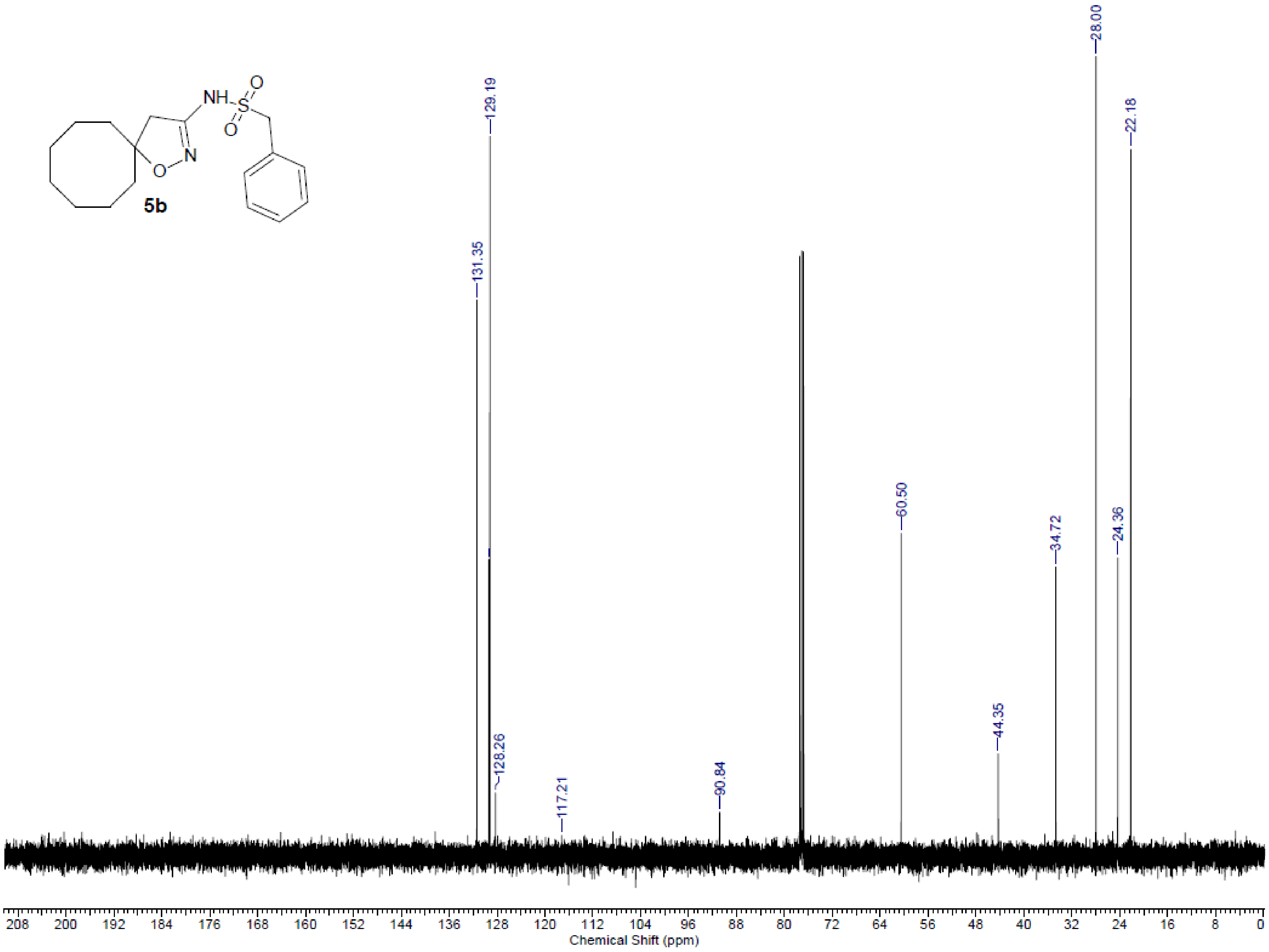

HSQC ($CDCl_3$) spectrum of compound **5b**

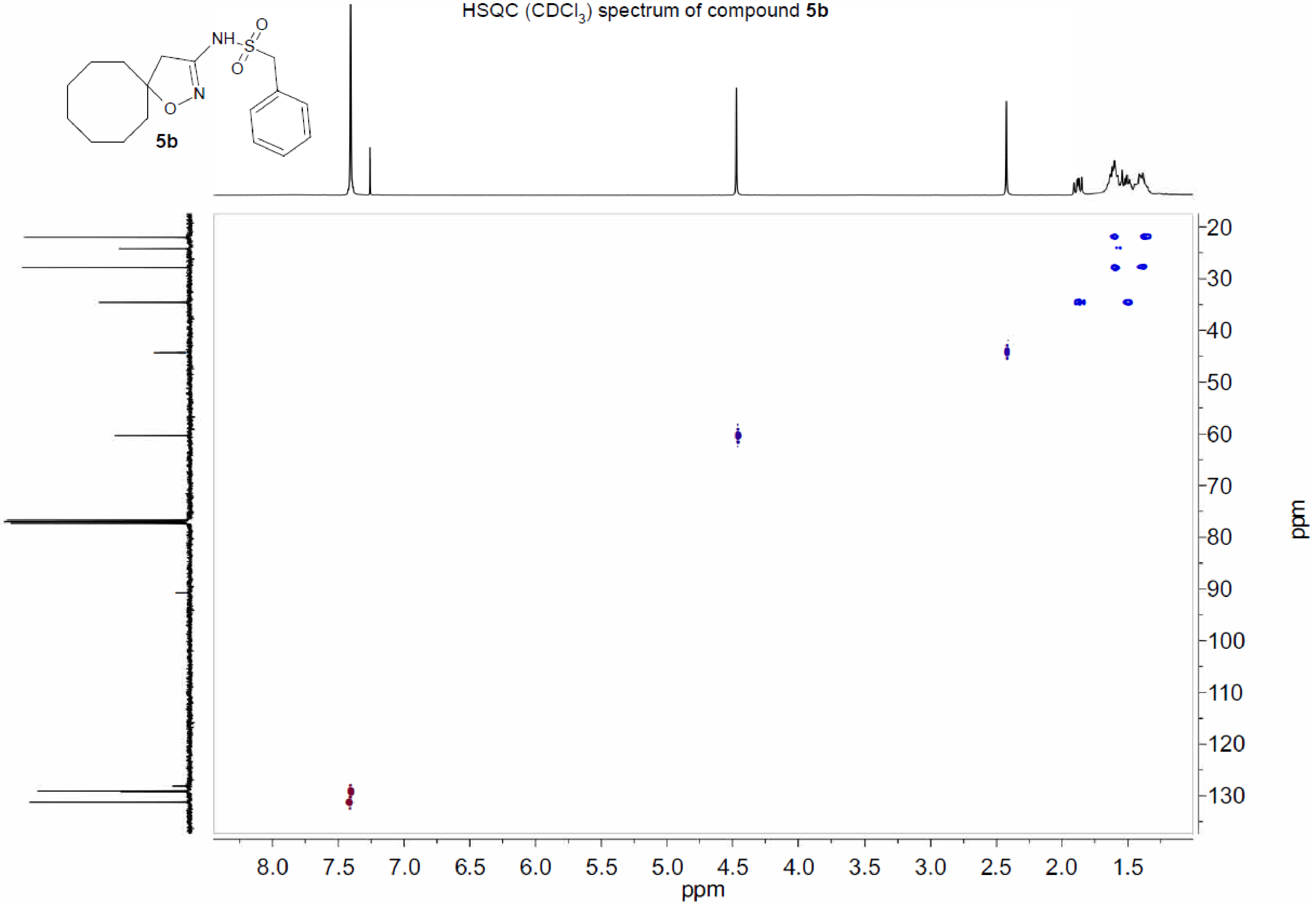

HMBC ($CDCl_3$) spectrum of compound **5b**

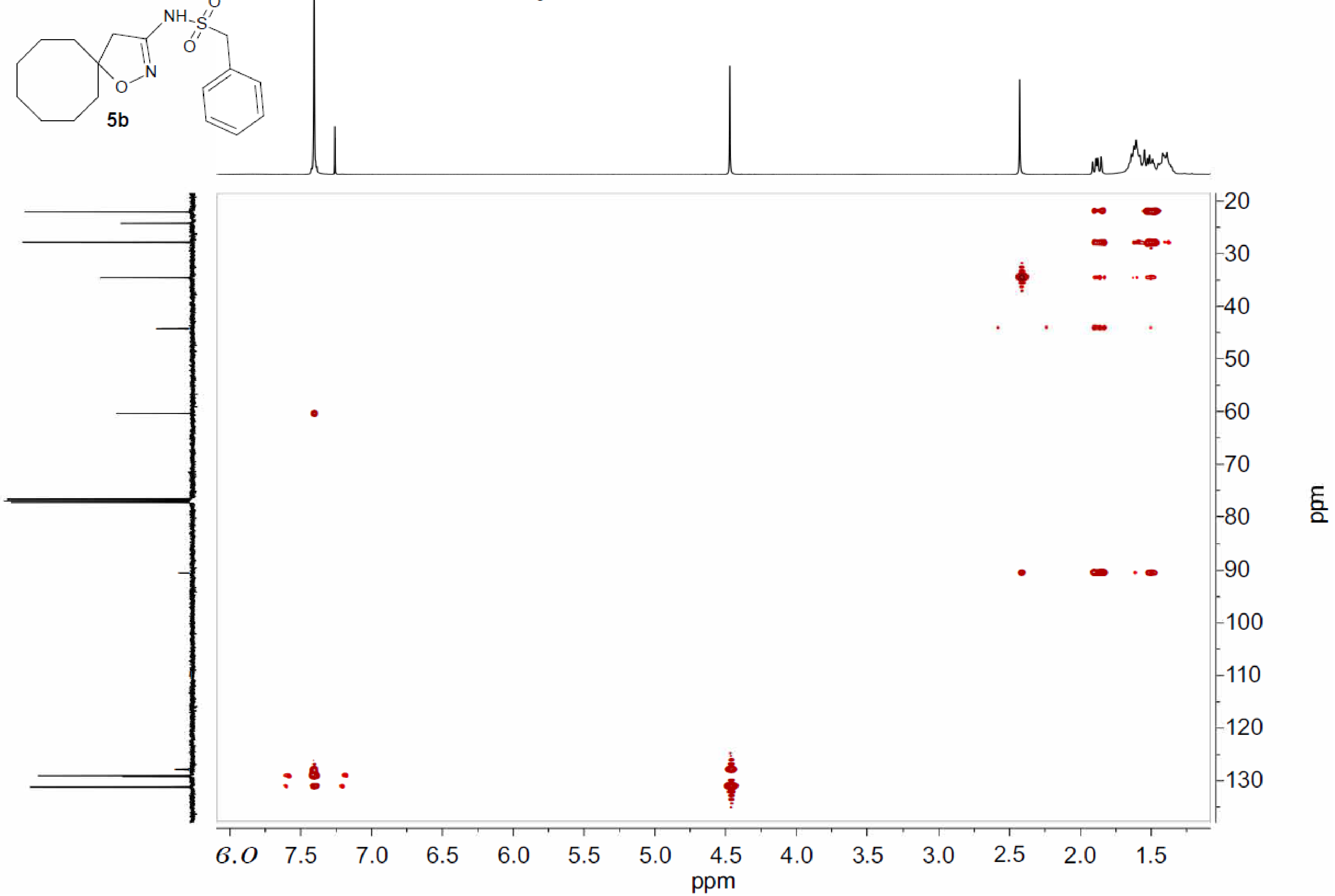

^{1}H NMR ($CDCl_3$) spectrum of compound **5c**

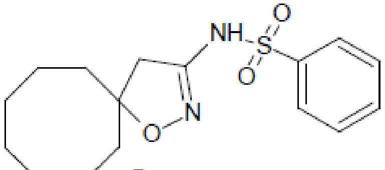

5c

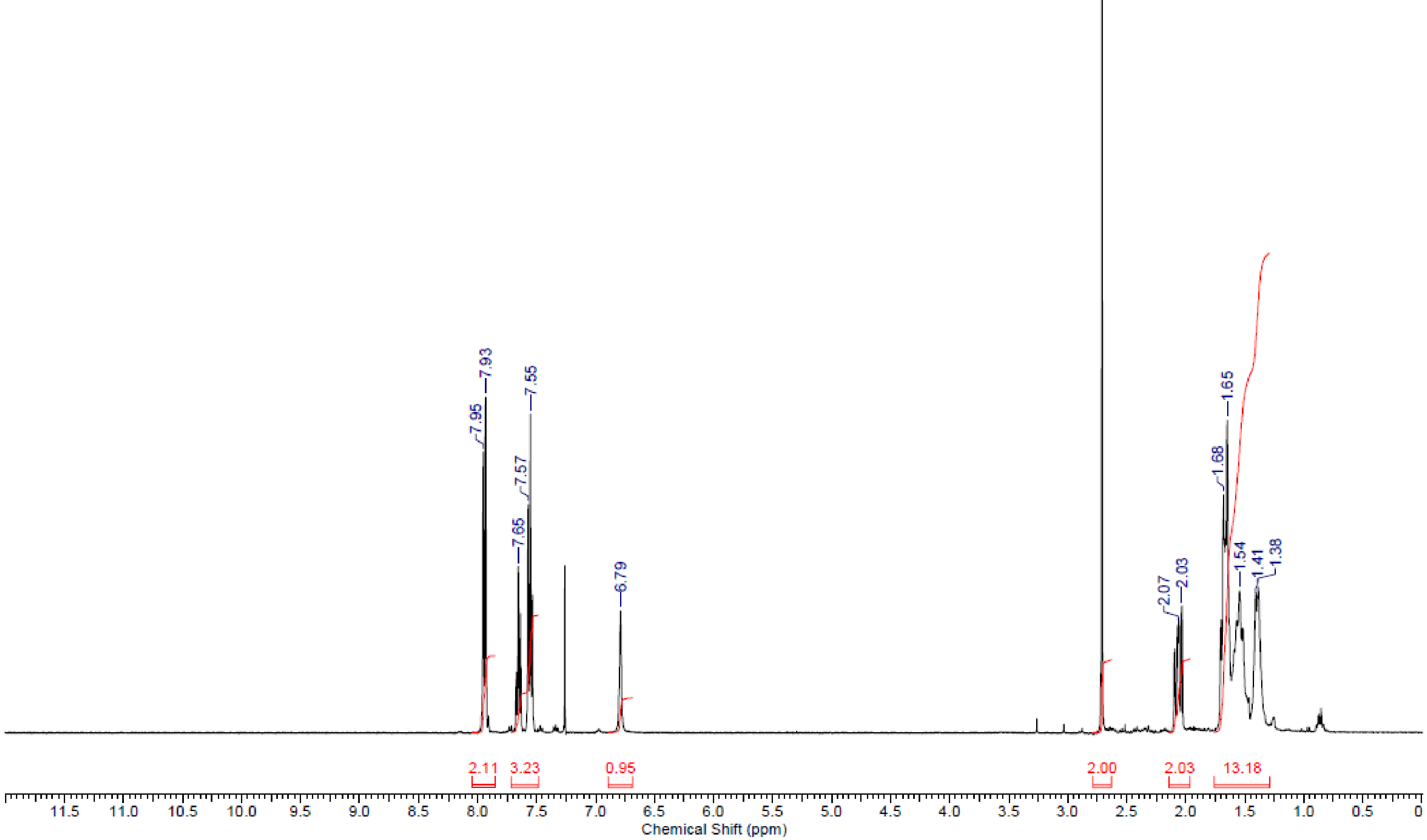

^{13}C NMR ($CDCl_3$) spectrum of compound **5c**

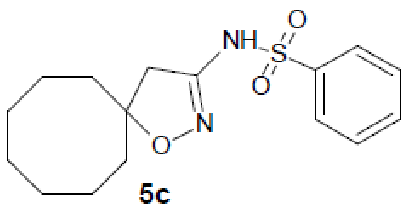

5c

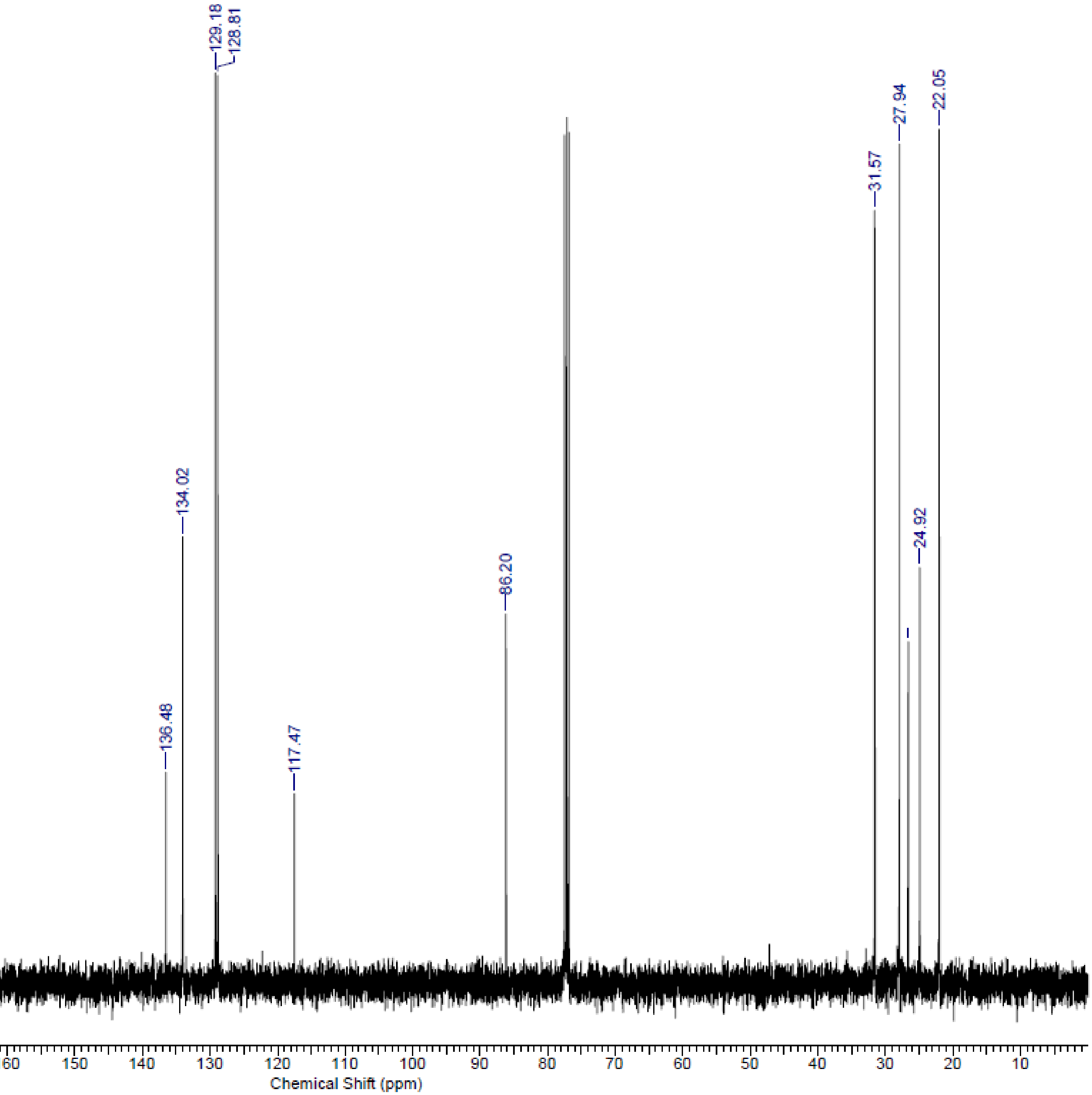

^{1}H NMR ($CDCl_3$) spectrum of compound **5d**

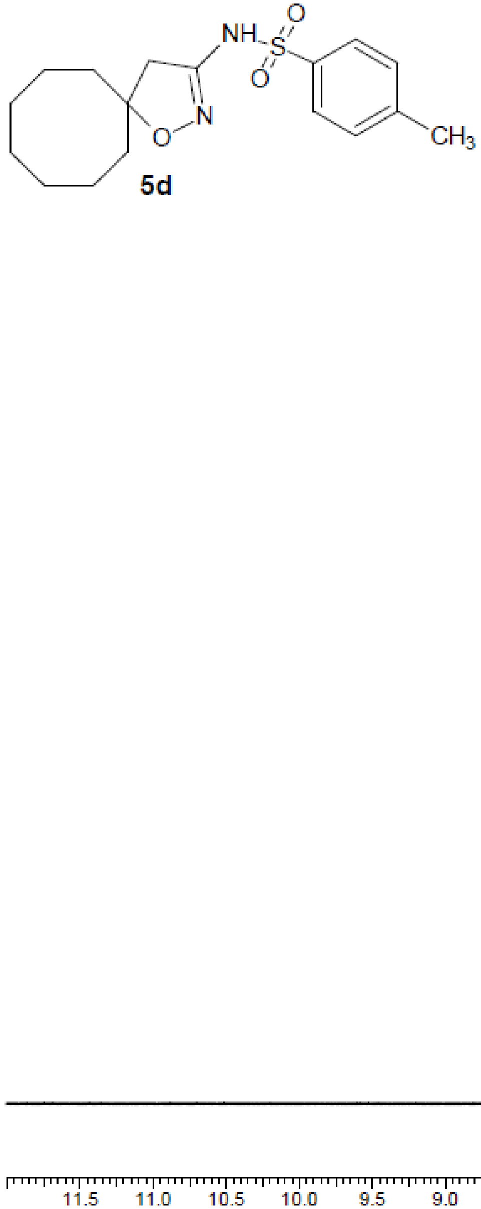

5d

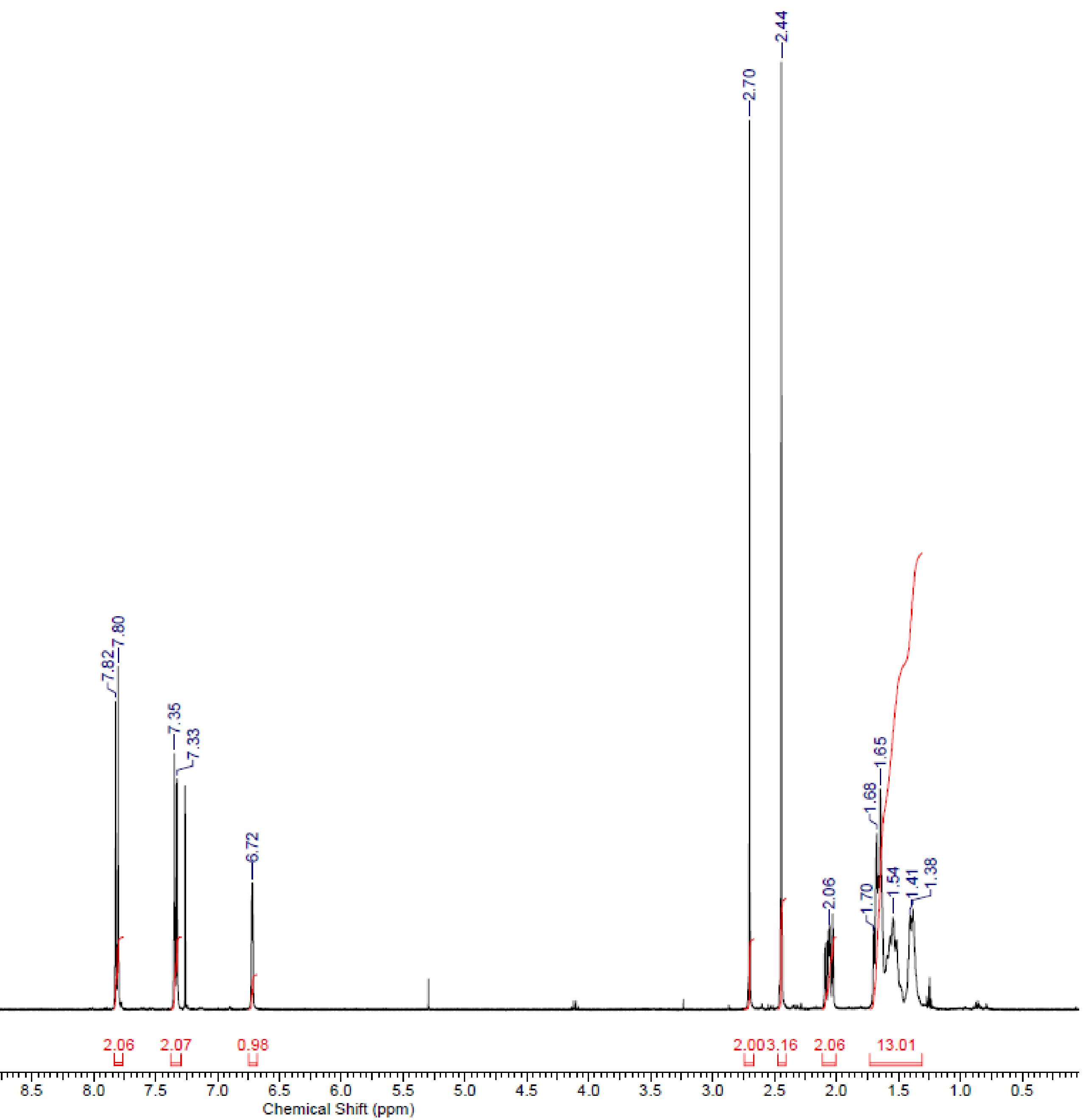

^{13}C NMR ($CDCl_3$) spectrum of compound **5d**

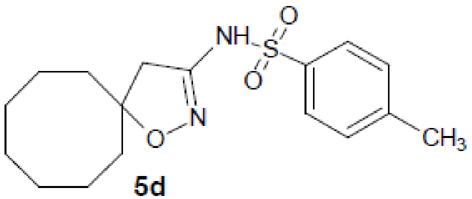

5d

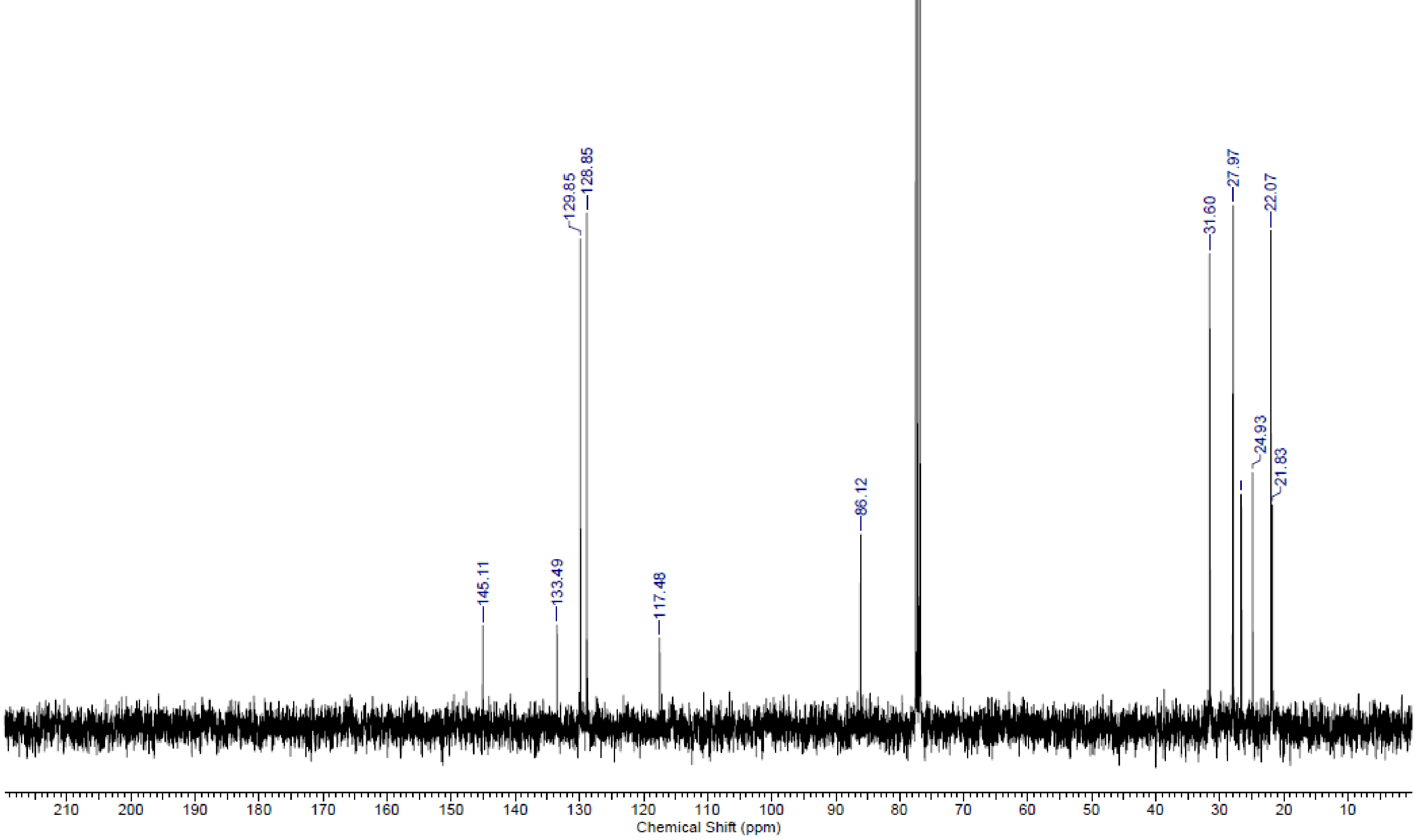

^{1}H NMR ($CDCl_3$) spectrum of compound **5e**

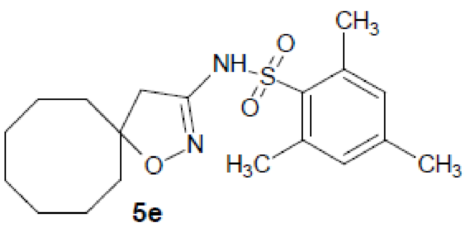

5e

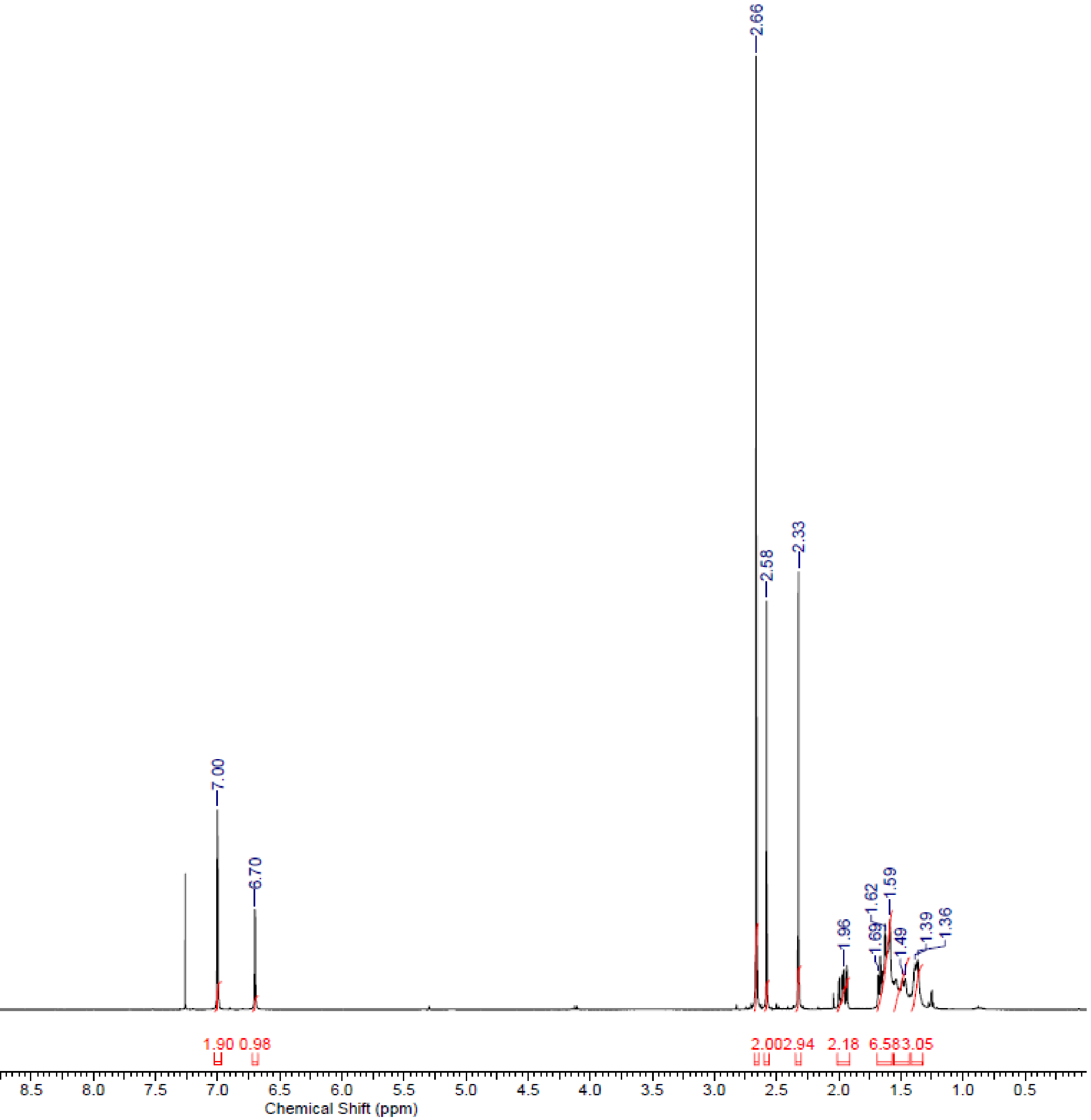

^{13}C NMR ($CDCl_3$) spectrum of compound **5e**

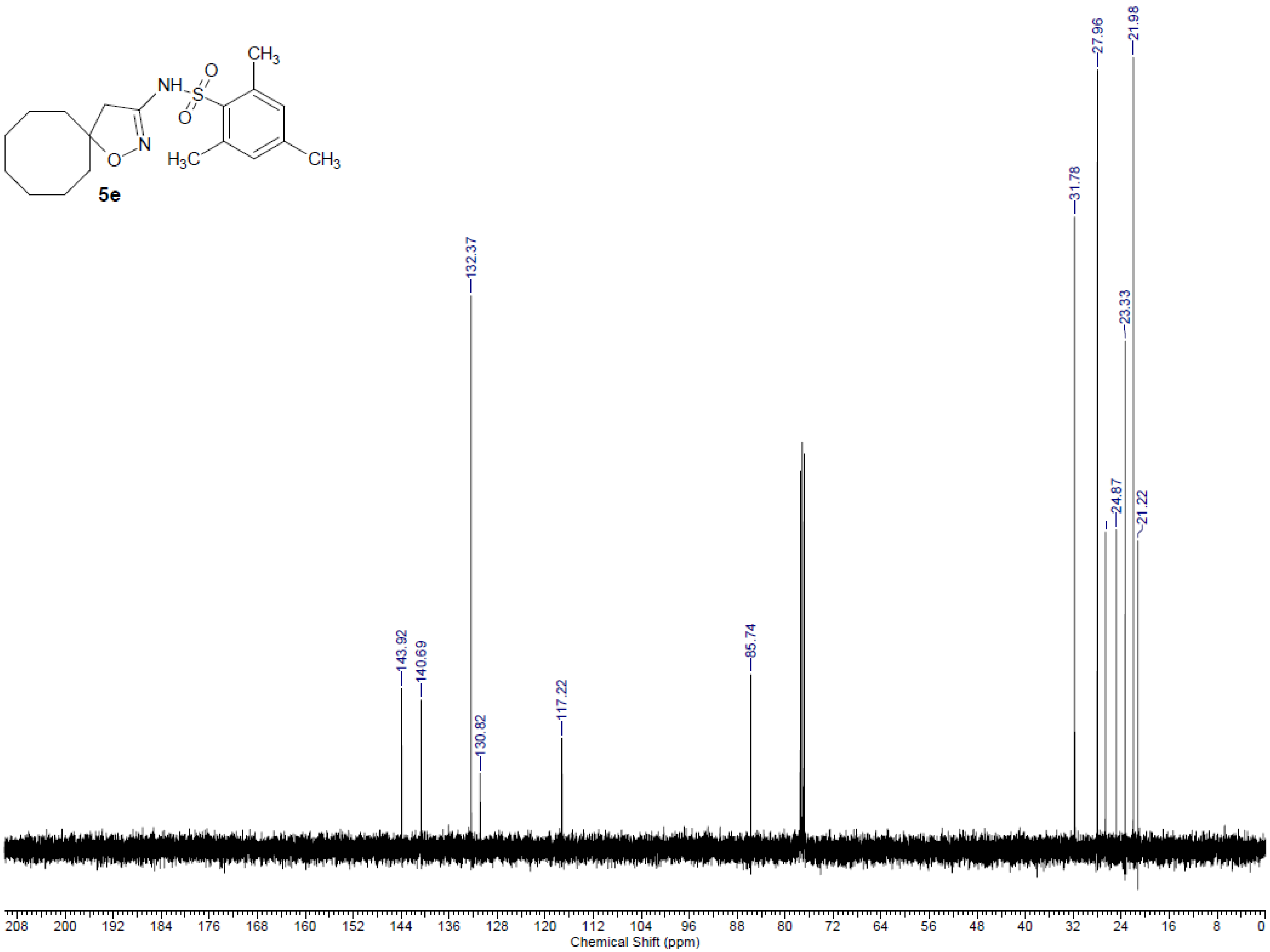

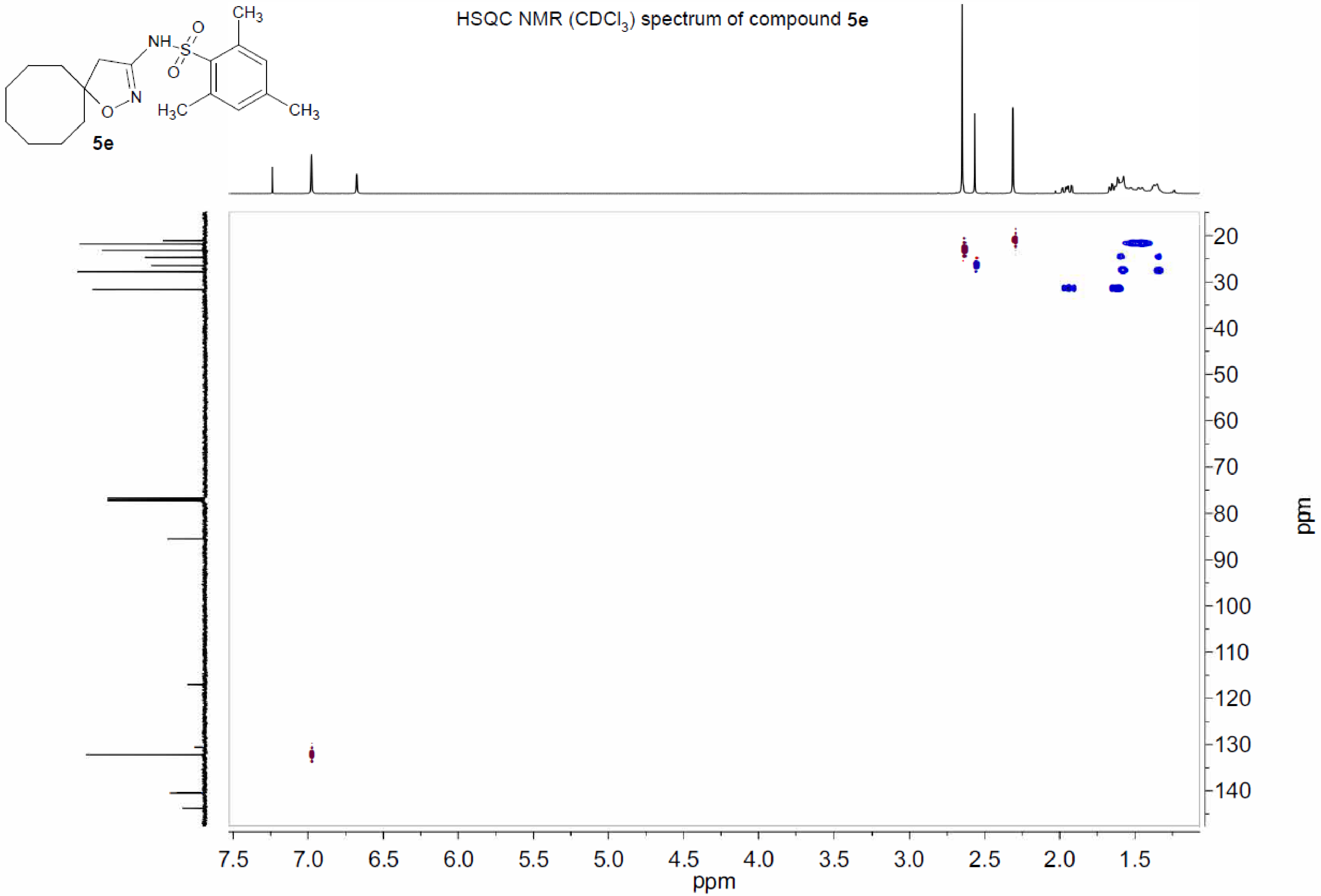
HSQC NMR (CDCl3) spectrum of compound 5e
CH3
NH
S
O
O
N
O
H3C
CH3
5e
ppm
ppm

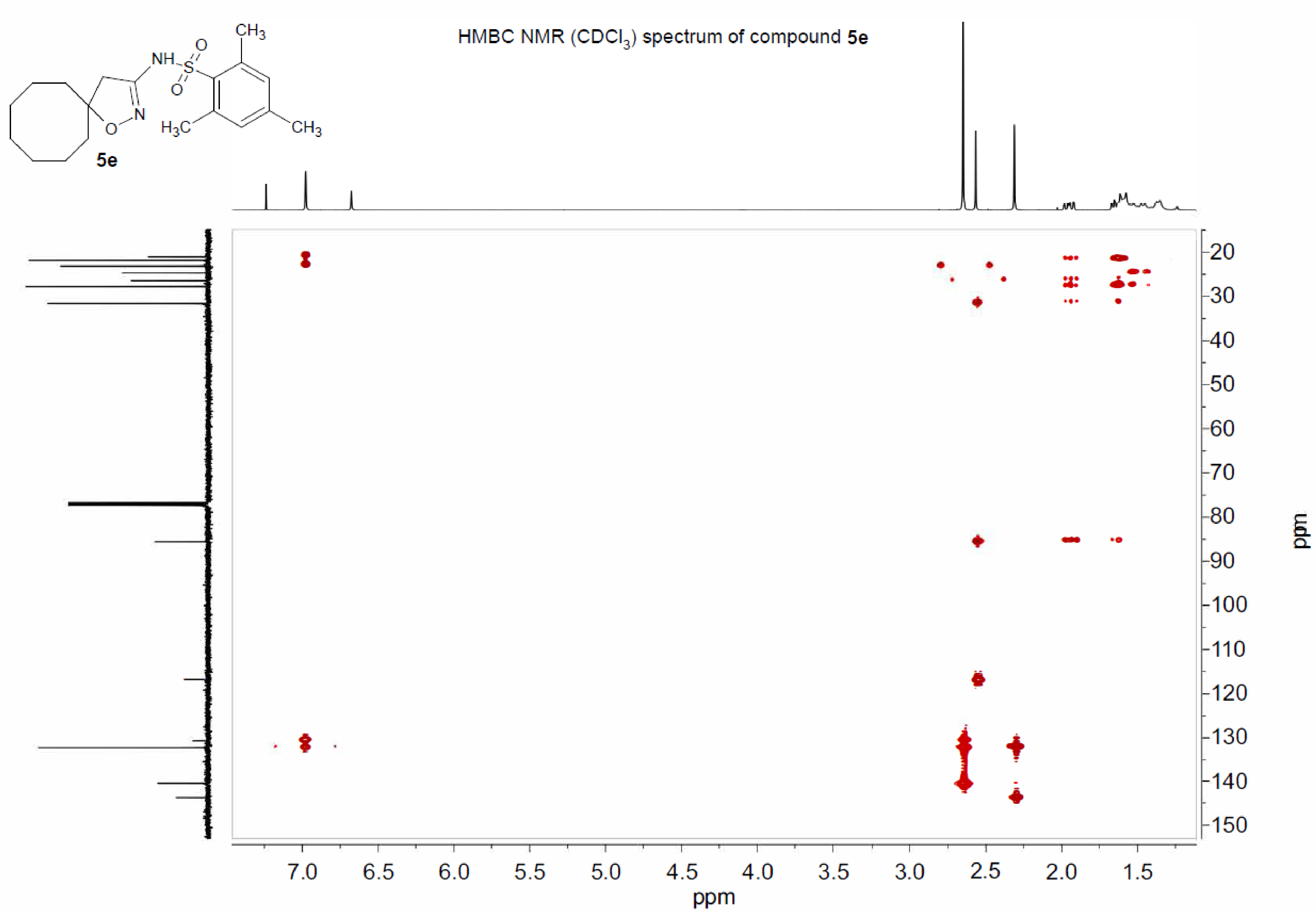
HMBC NMR (CDCl3) spectrum of compound 5e
5e
ppm
ppm

^{1}H NMR ($CDCl_3$) spectrum of compound **5f**

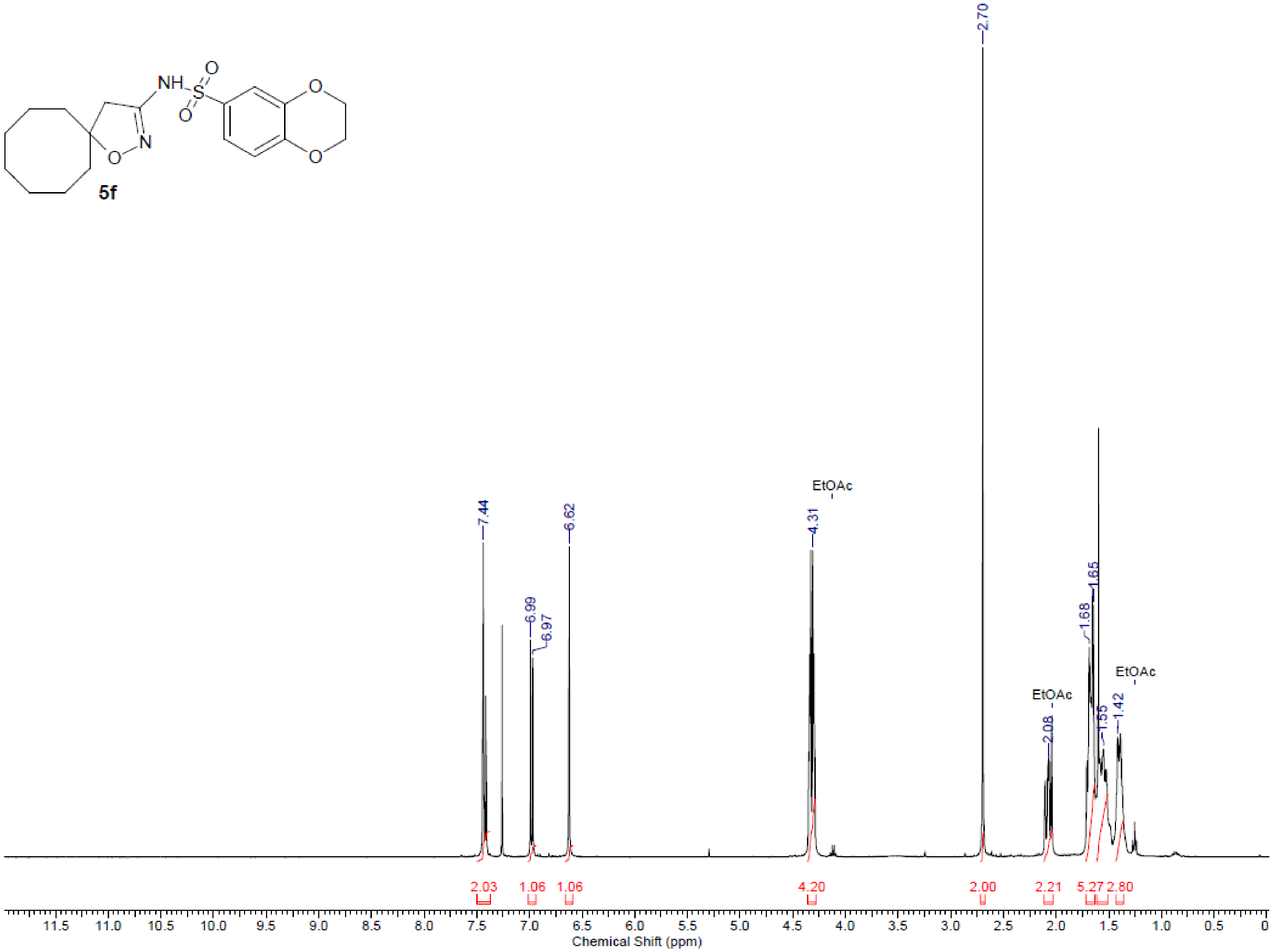

^{13}C NMR ($CDCl_3$) spectrum of compound **5f**

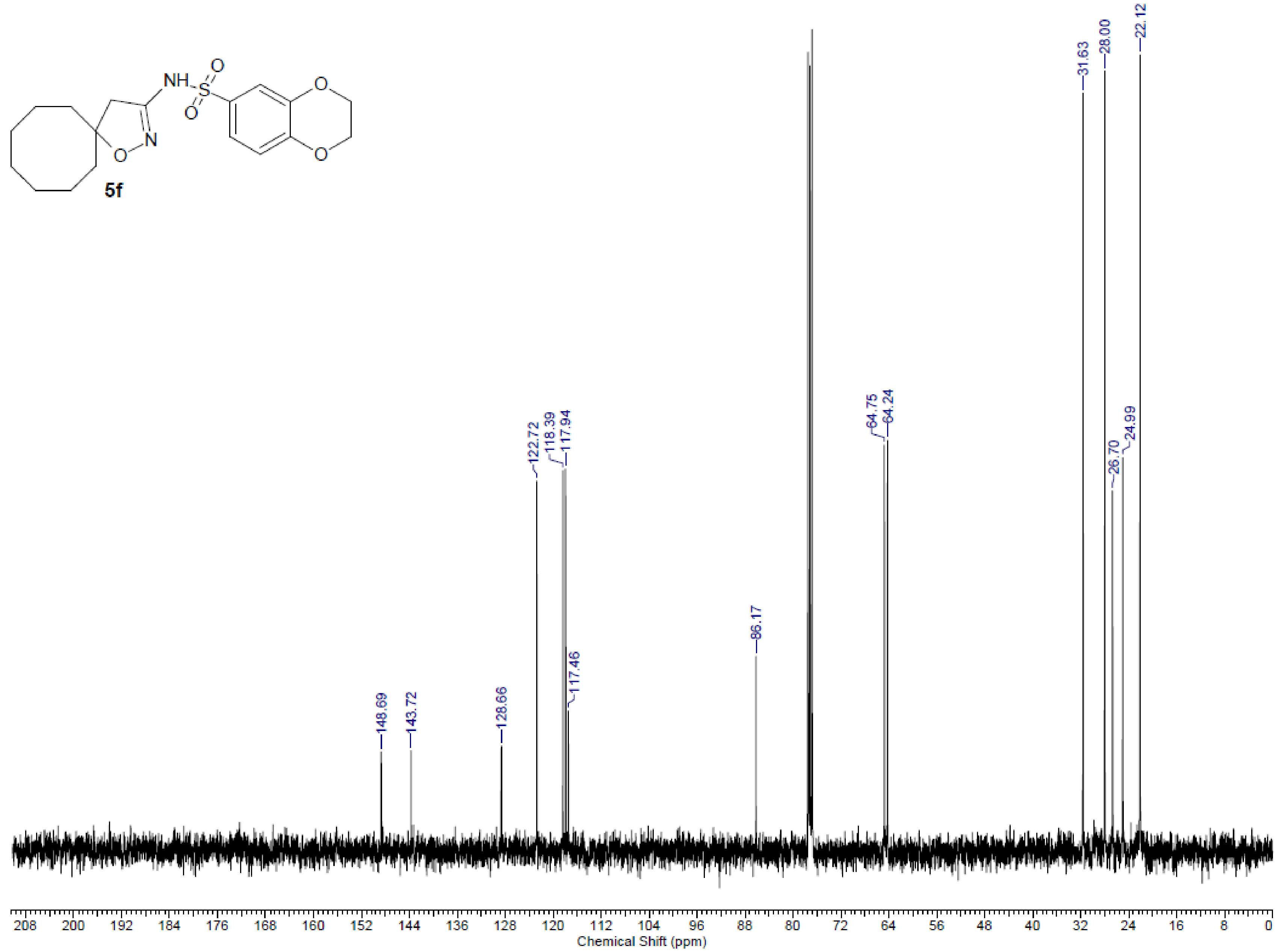

HSQC NMR ($CDCl_3$) spectrum of compound **5f**

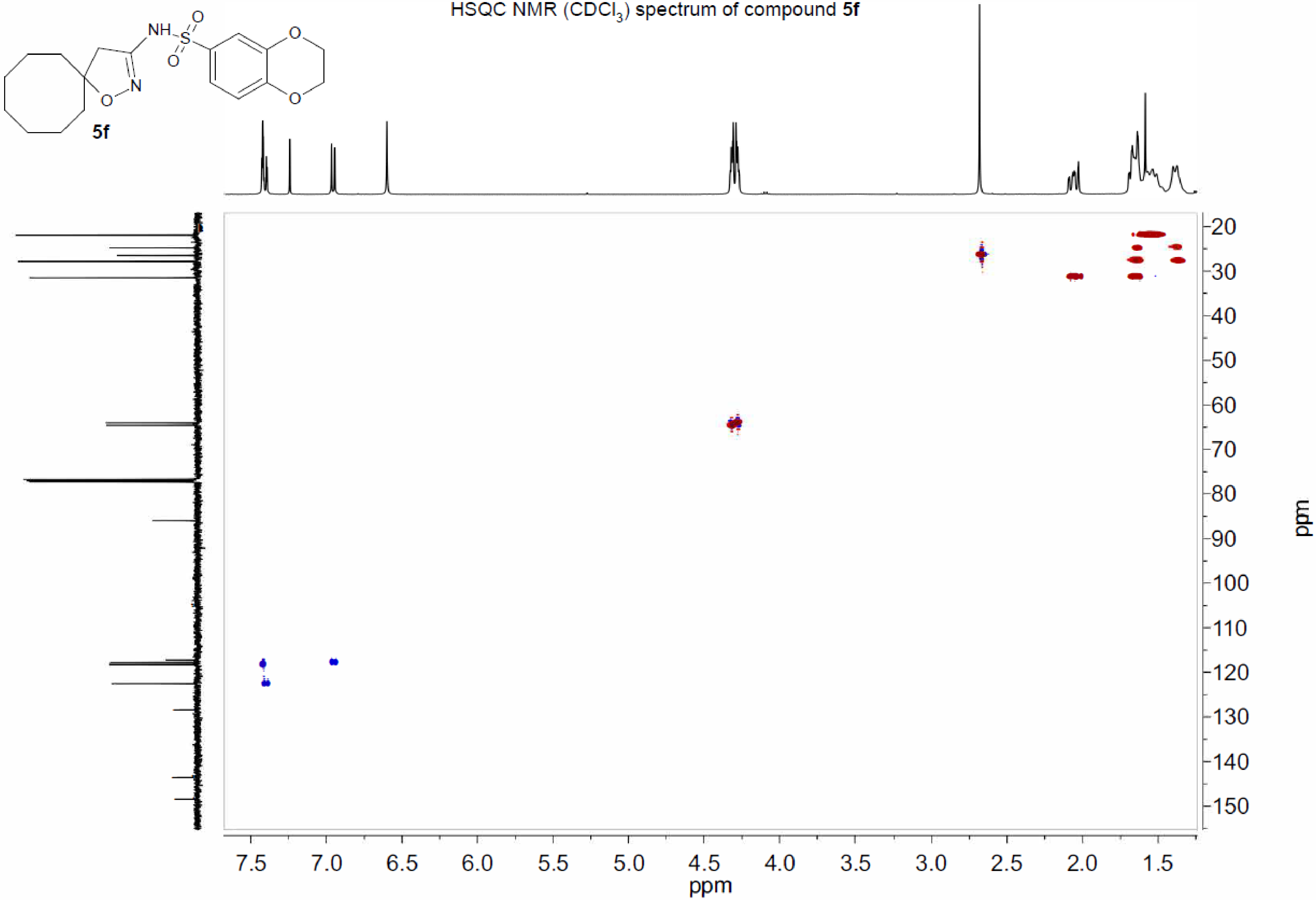

HMBC NMR ($CDCl_3$) spectrum of compound **5f**

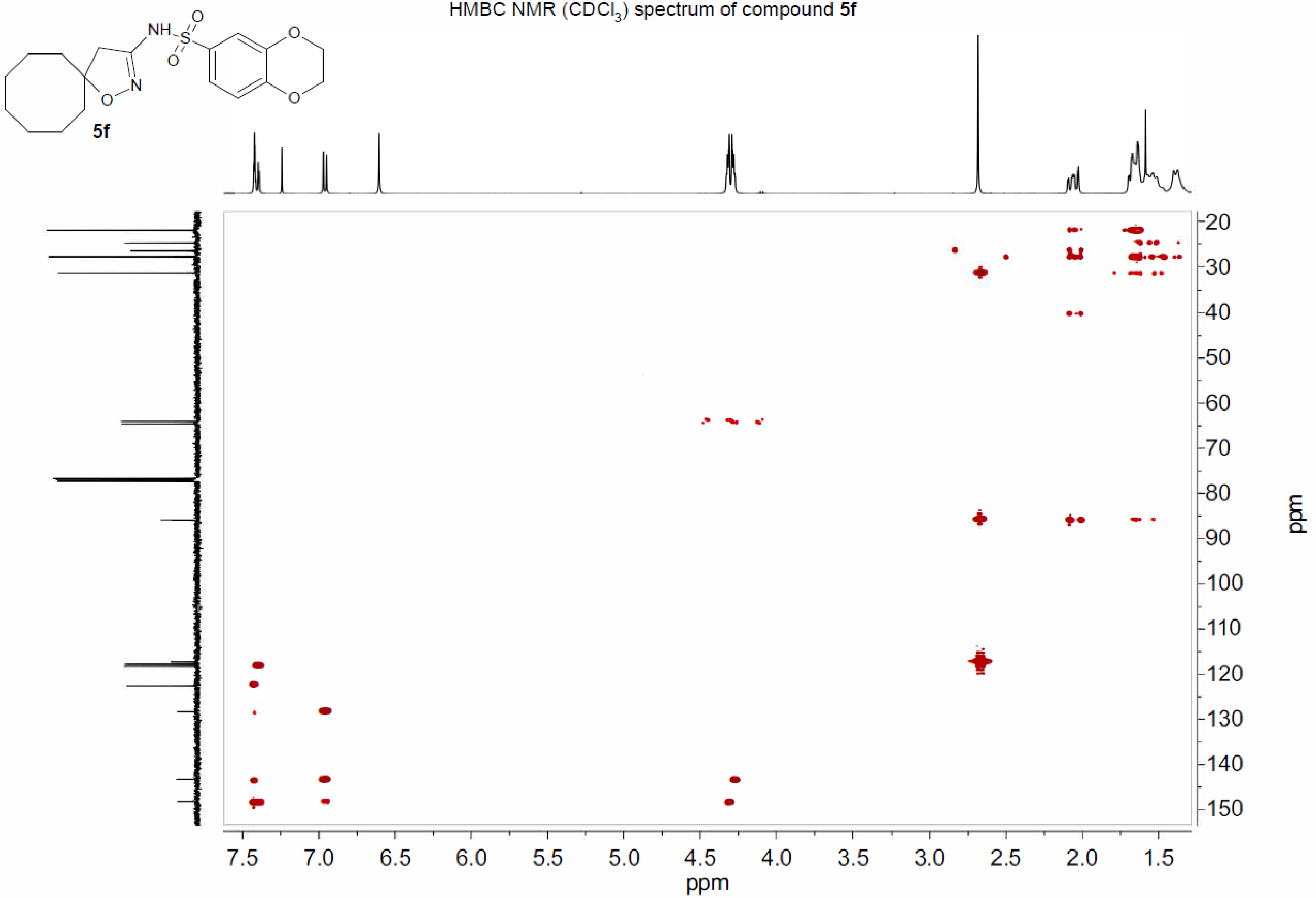

^{1}H NMR (CD_3OD) spectrum of compound **5g**

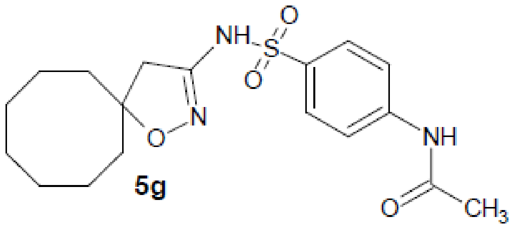

5g

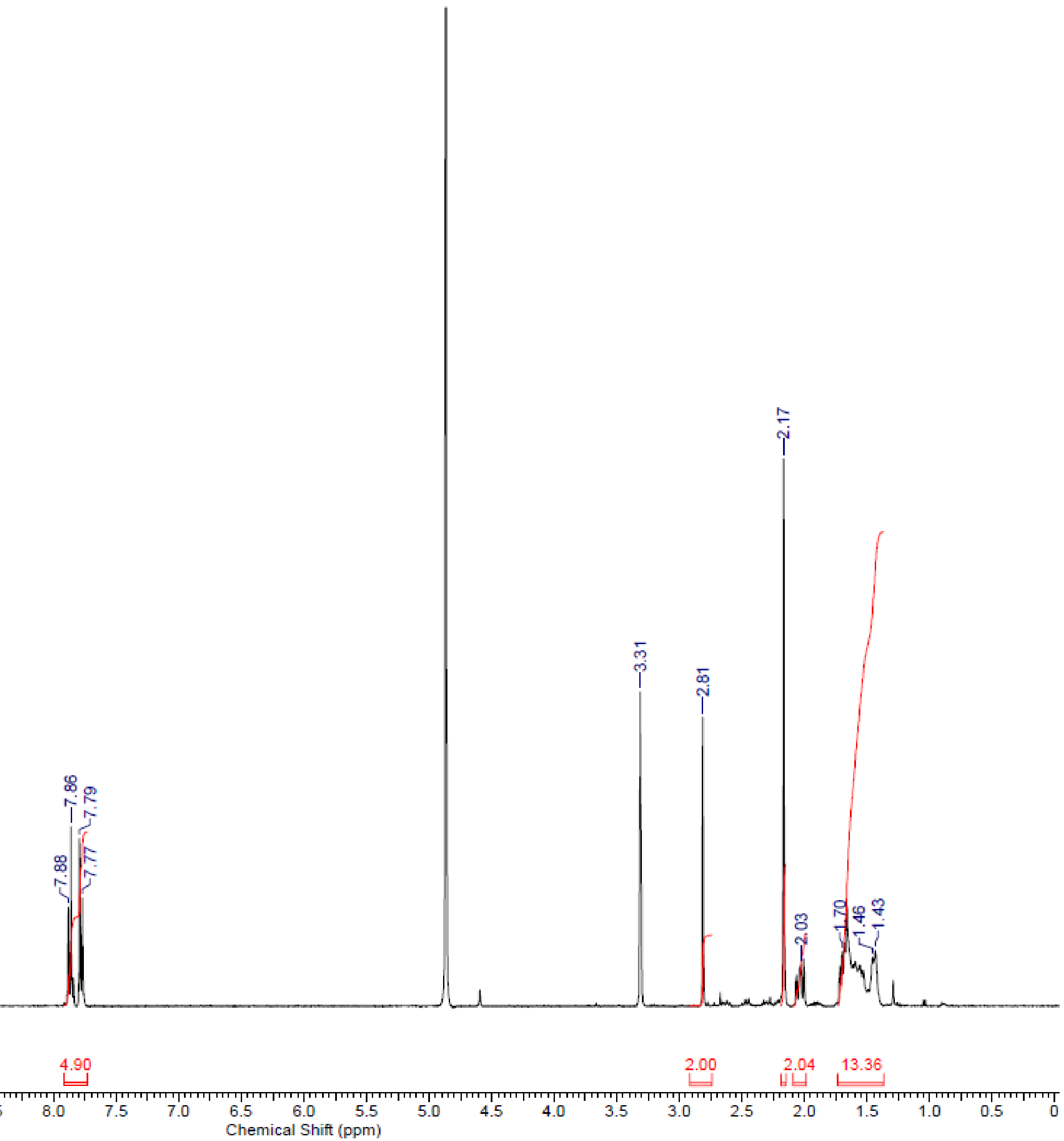

^{13}C NMR (CD_3OD) spectrum of compound **5g**

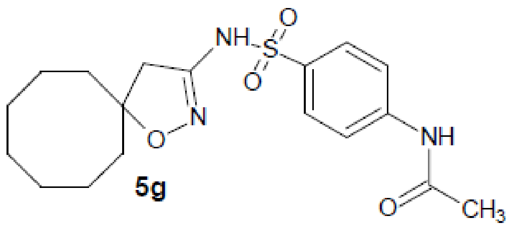

5g

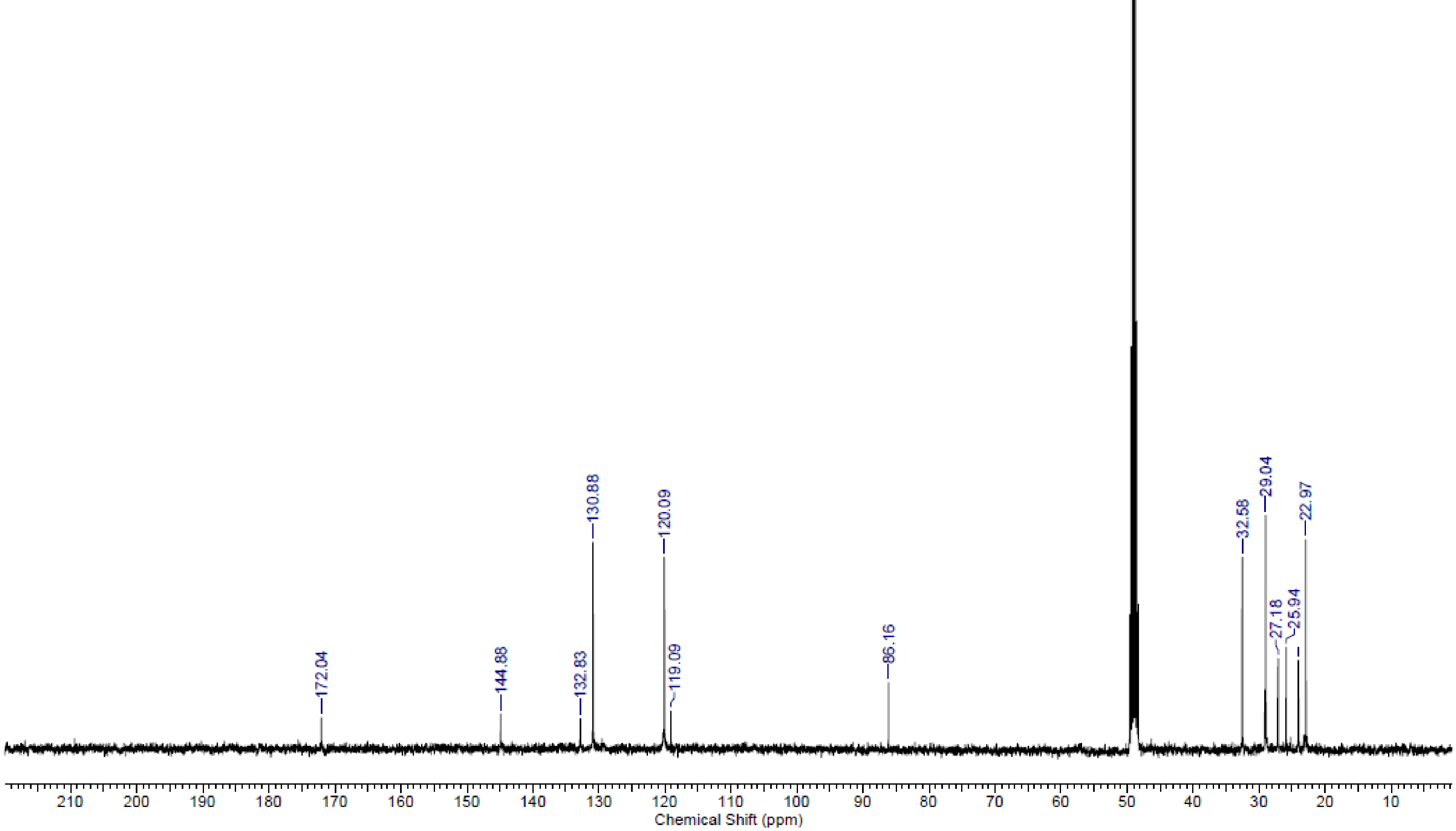

HSQC NMR (CD_3OD) spectrum of compound **5g**

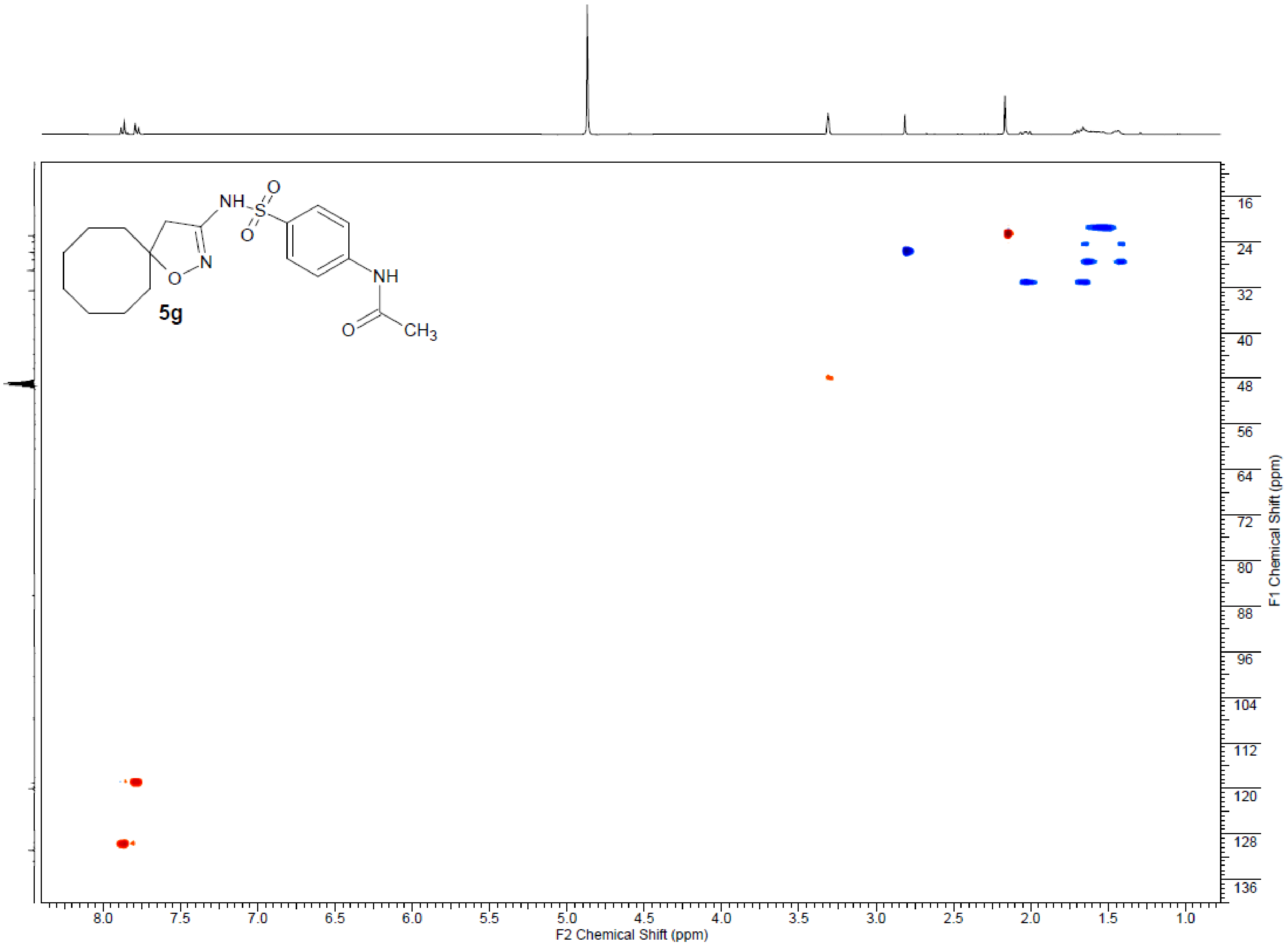

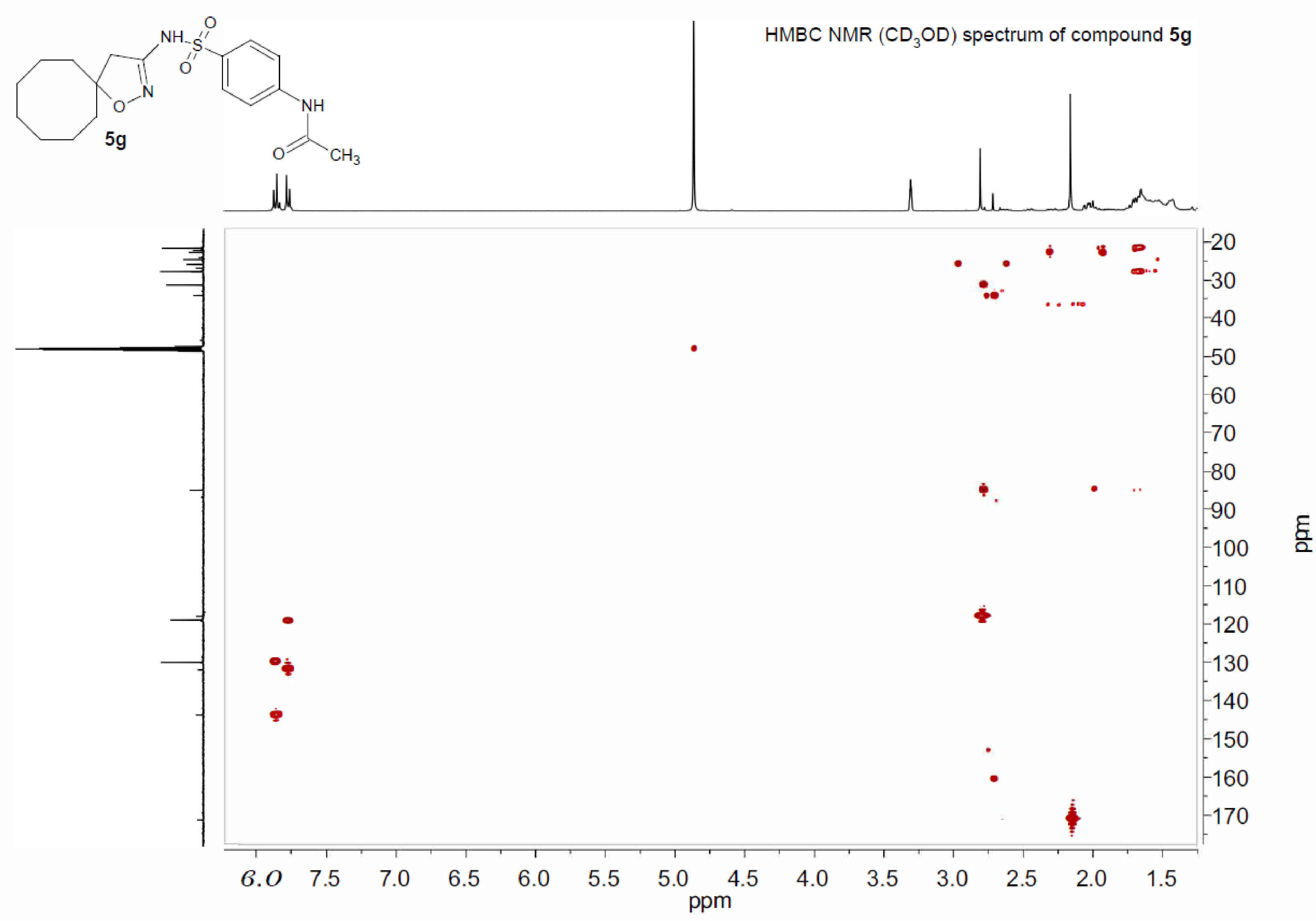
HMBC NMR (CD_3OD) spectrum of compound **5g**
5g
NH
O
S
O
N
O
NH
O
CH_3
ppm
ppm

^{1}H NMR ($CDCl_3$) spectrum of compound **5h**

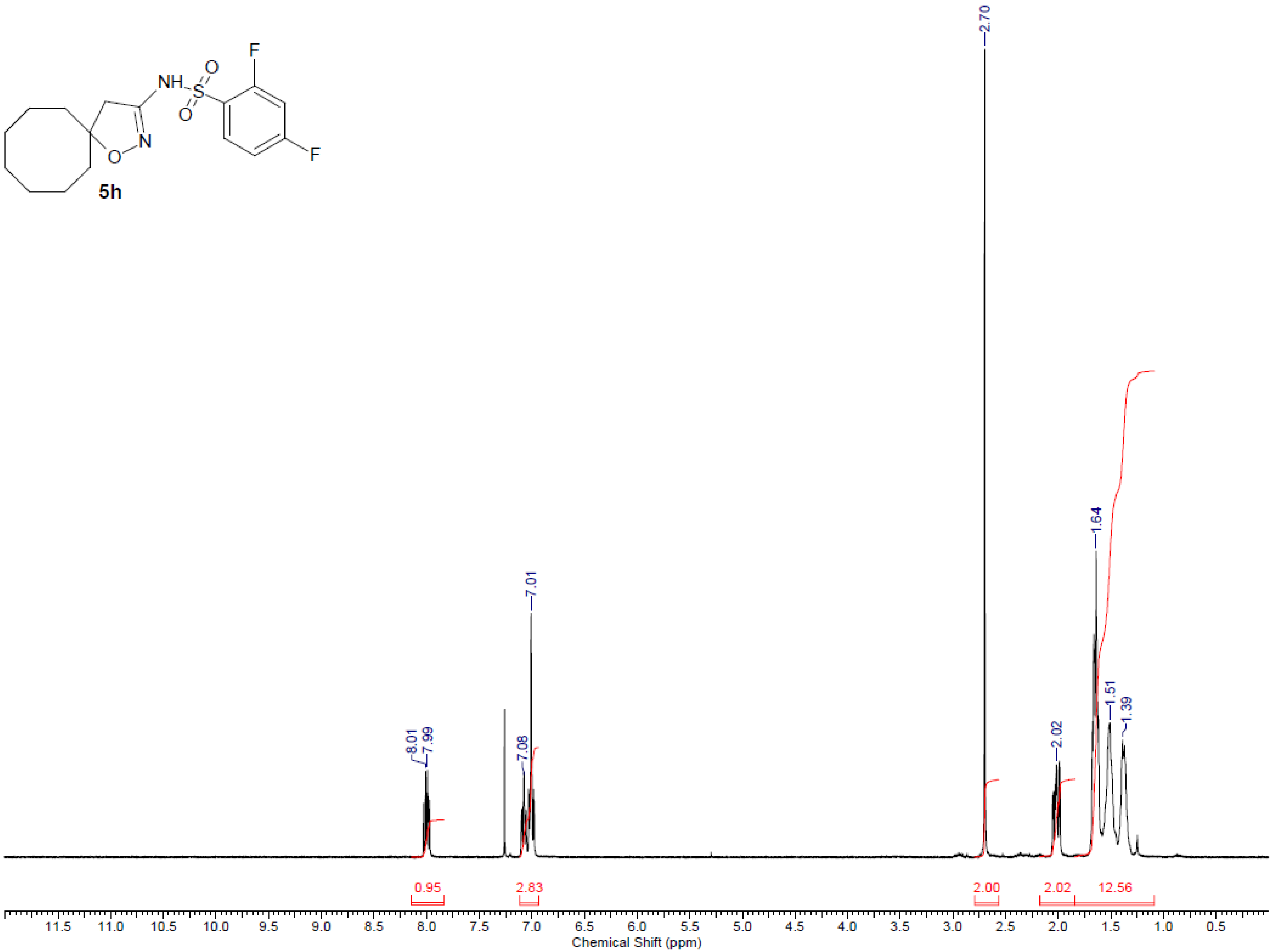

^{13}C NMR ($CDCl_3$) spectrum of compound **5h**

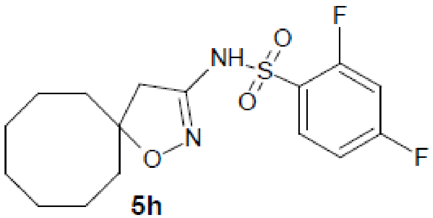

5h

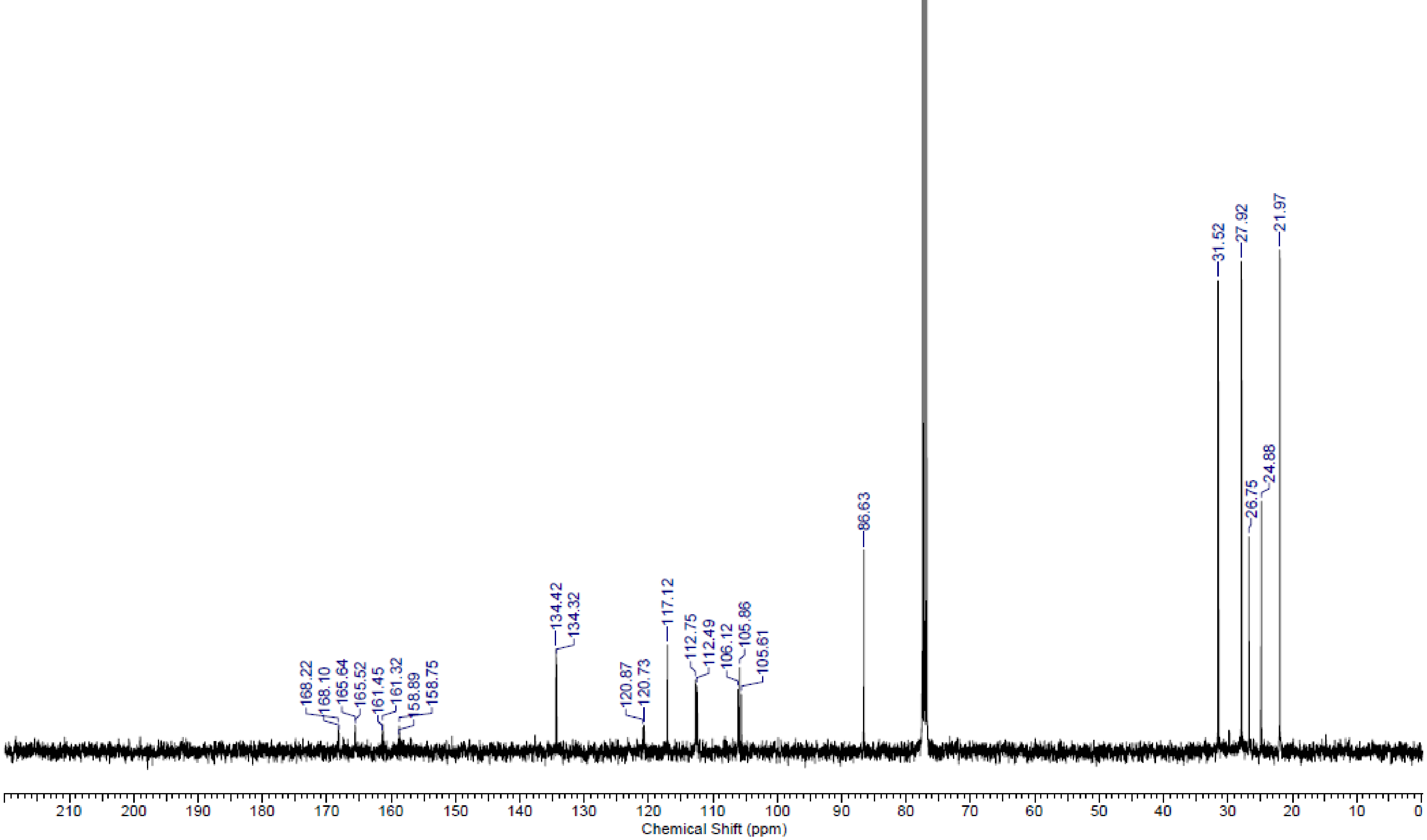

^{19}F NMR ($CDCl_3$) spectrum of compound **5h**

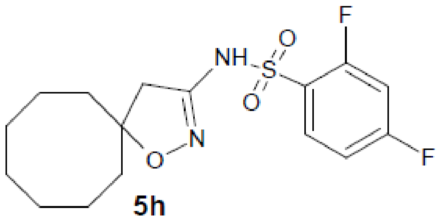

5h

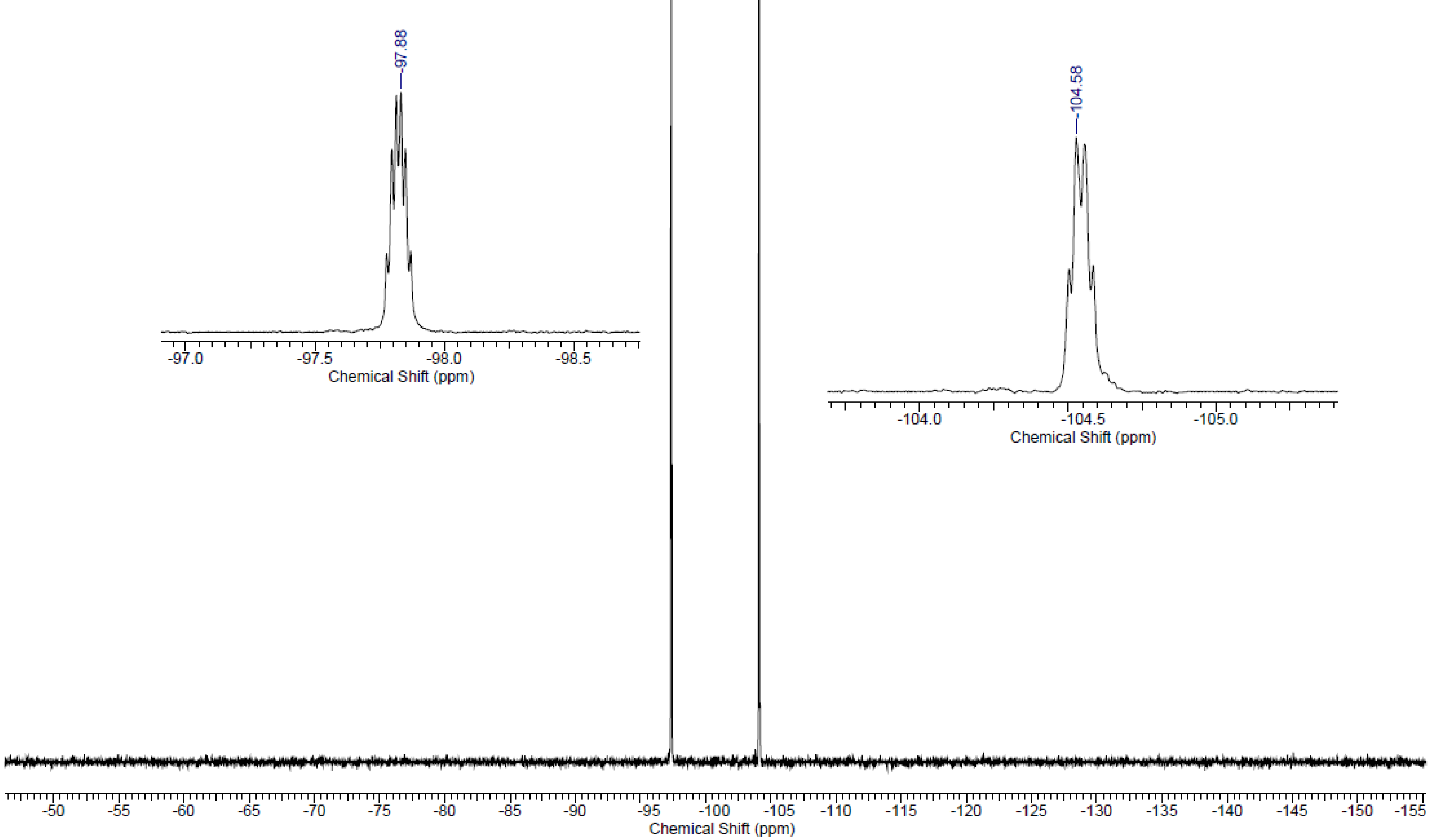

^{1}H NMR ($CDCl_3$) spectrum of compound **5i**

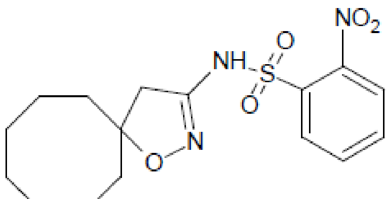

5i

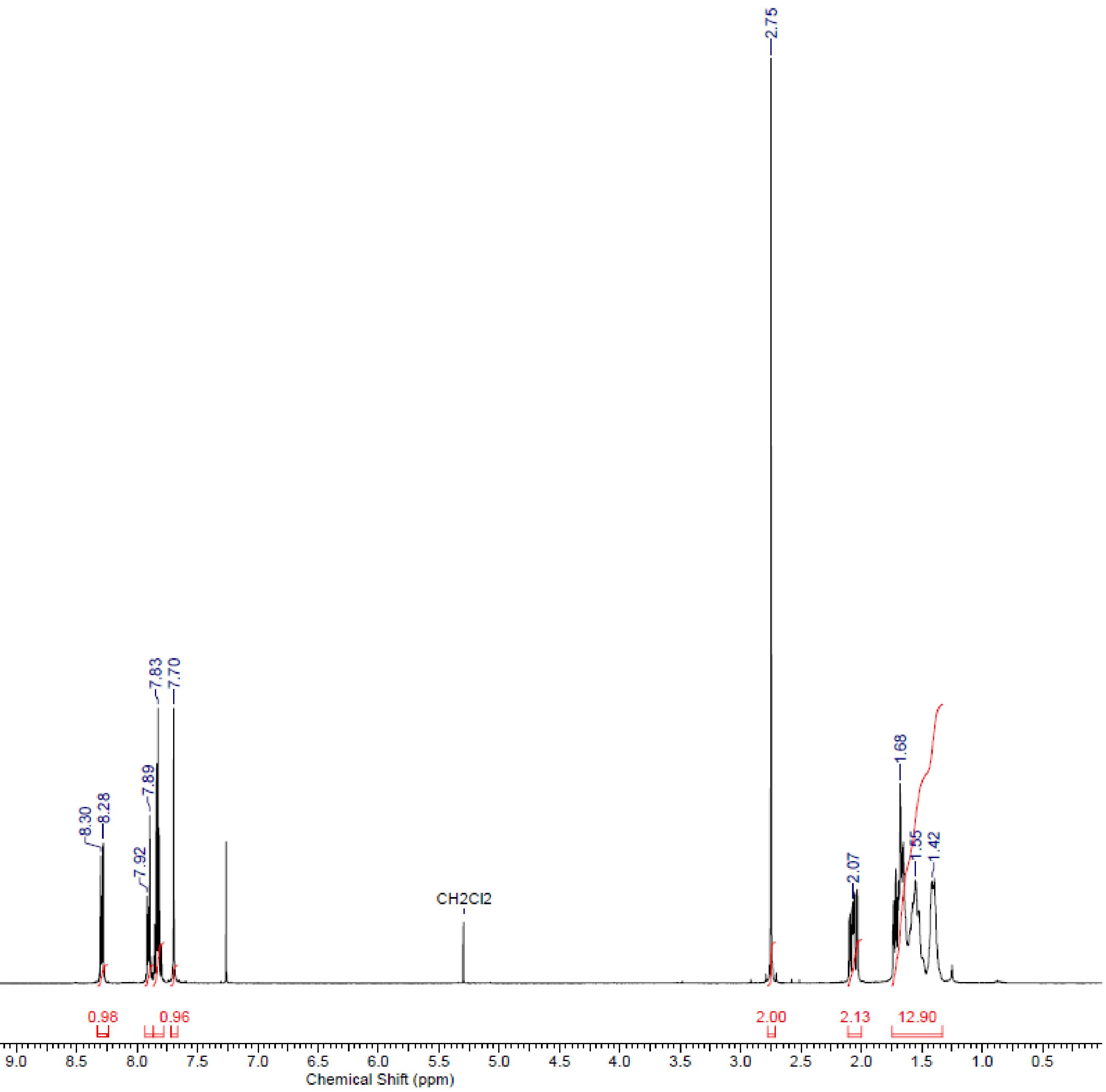

^{13}C NMR ($CDCl_3$) spectrum of compound **5i**

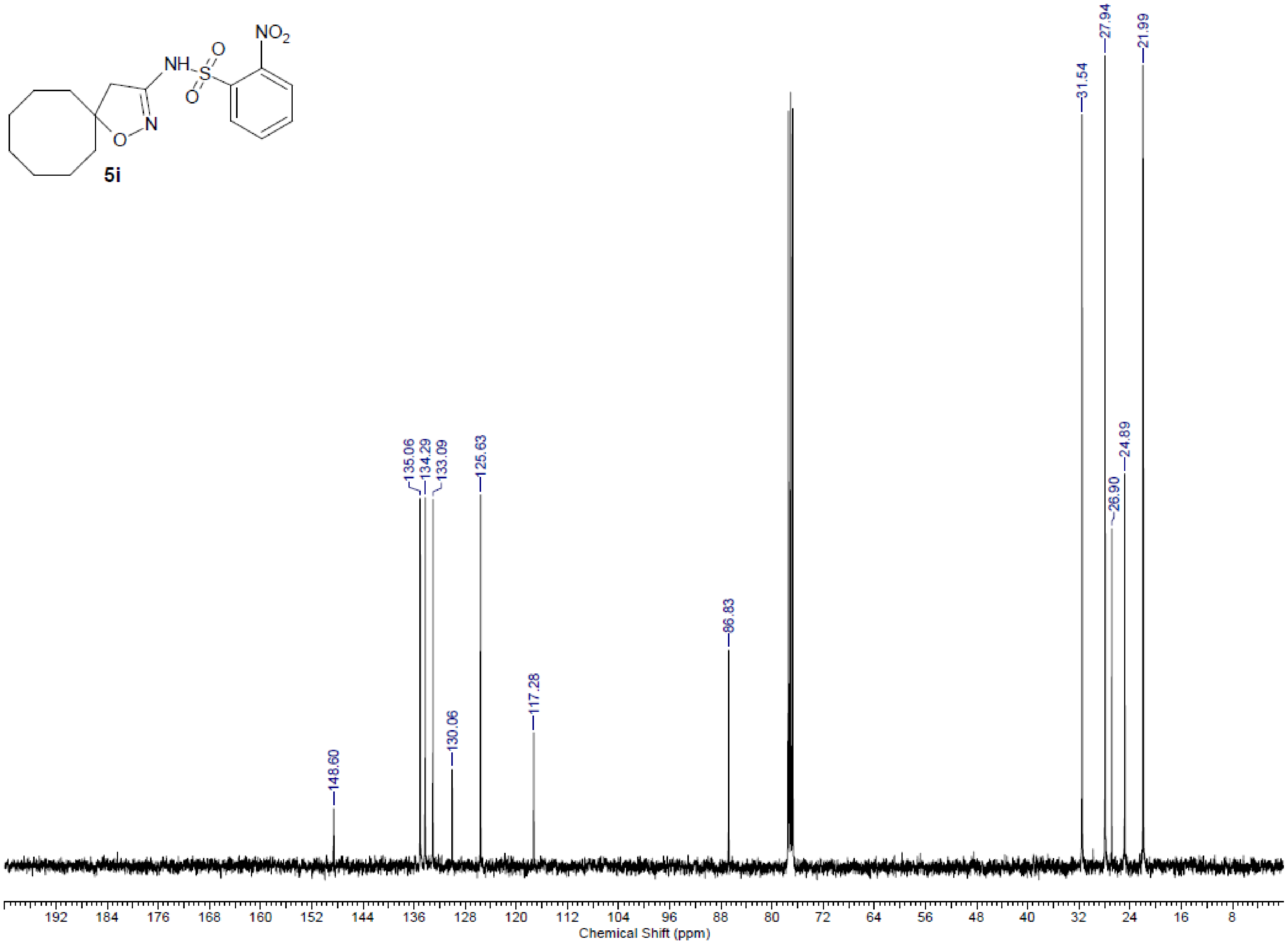

HSQC NMR ($CDCl_3$) spectrum of compound **5i**

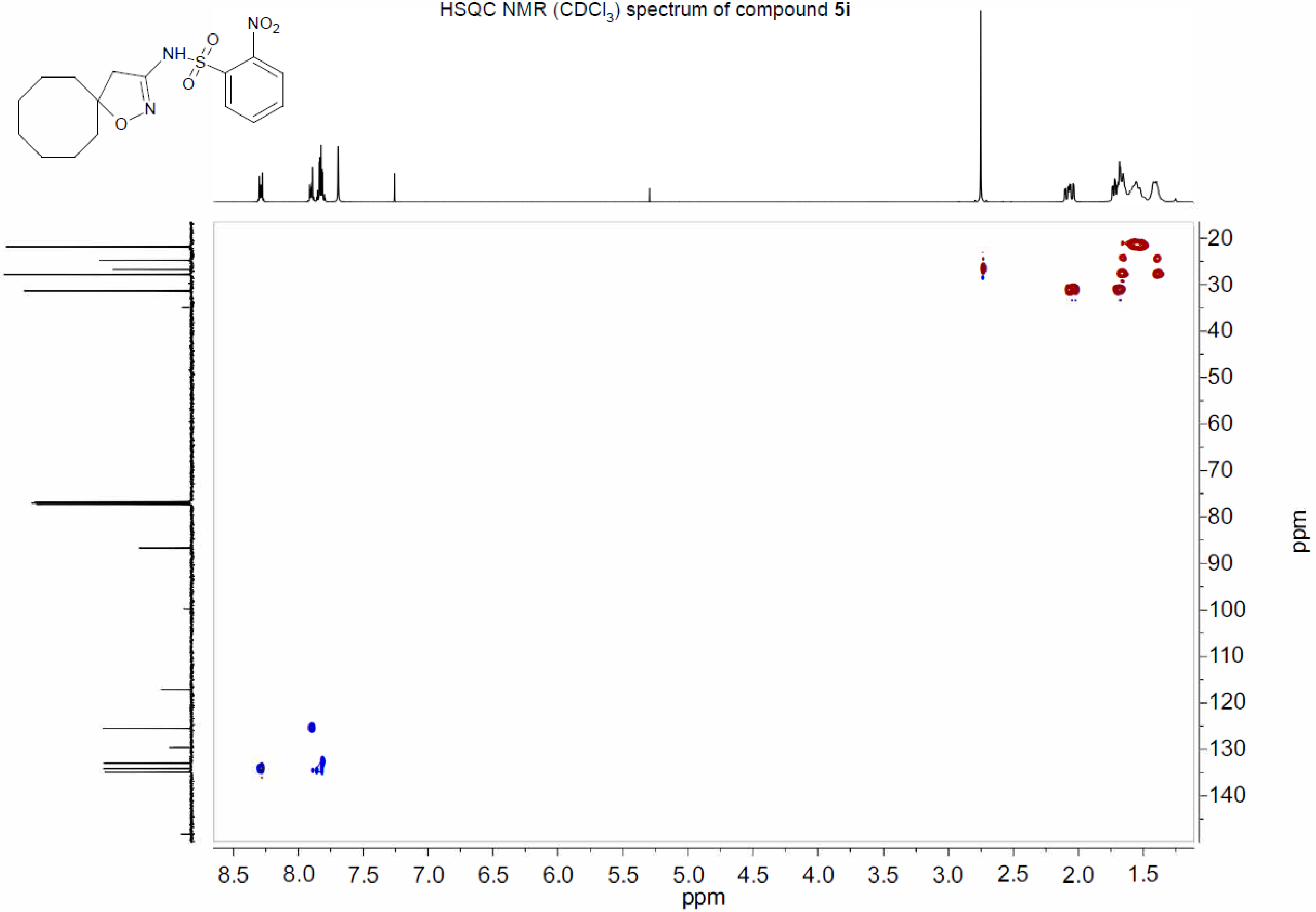

^{1}H NMR ($CDCl_3$) spectrum of compound **5j**

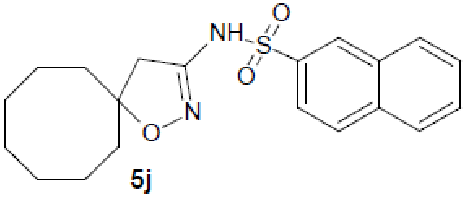

5j

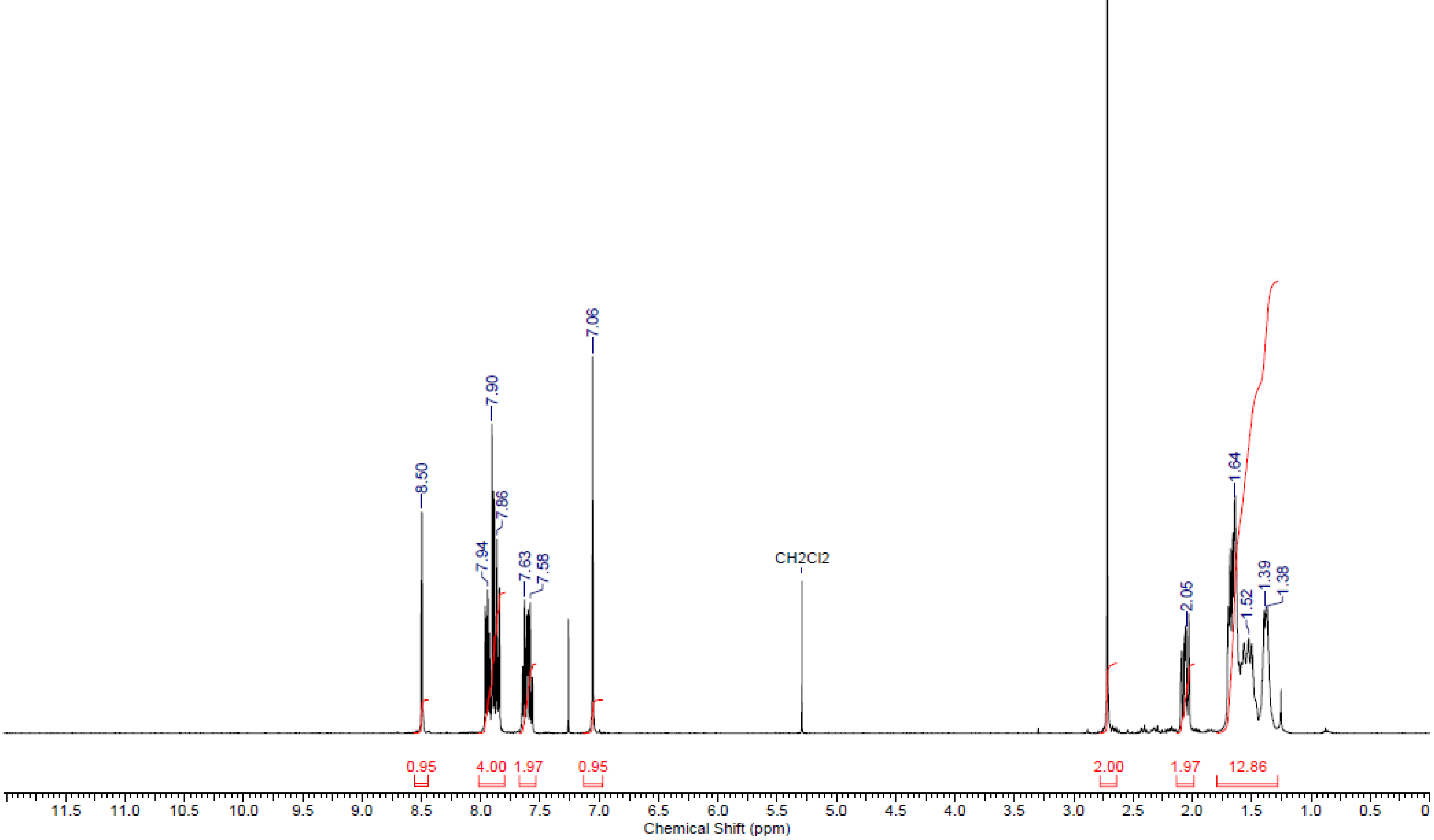

^{13}C NMR ($CDCl_3$) spectrum of compound **5j**

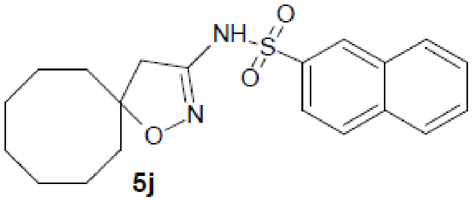

5j

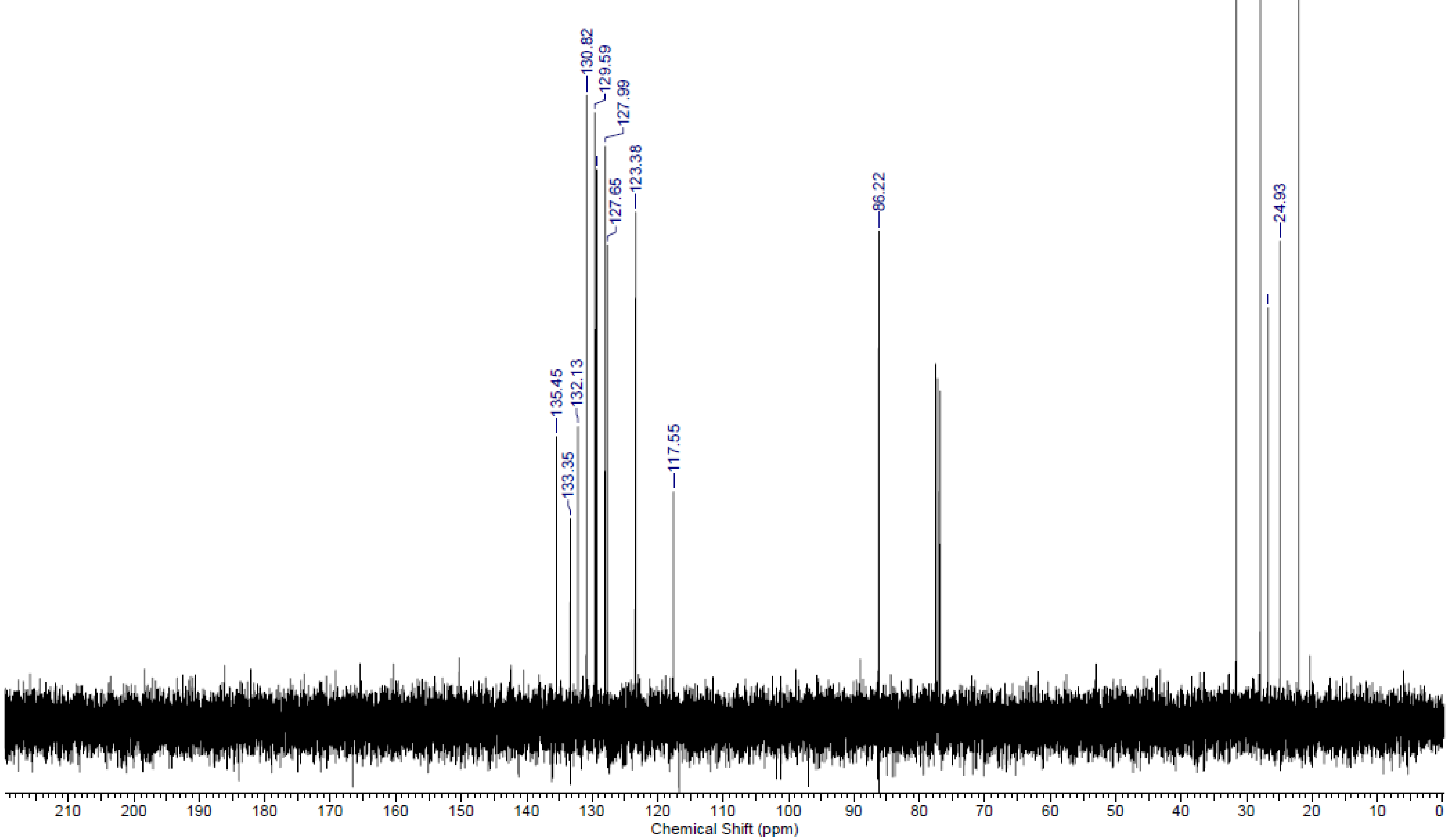

HSQC and HMBC NMR ($CDCl_3$) spectra of compound **5j**

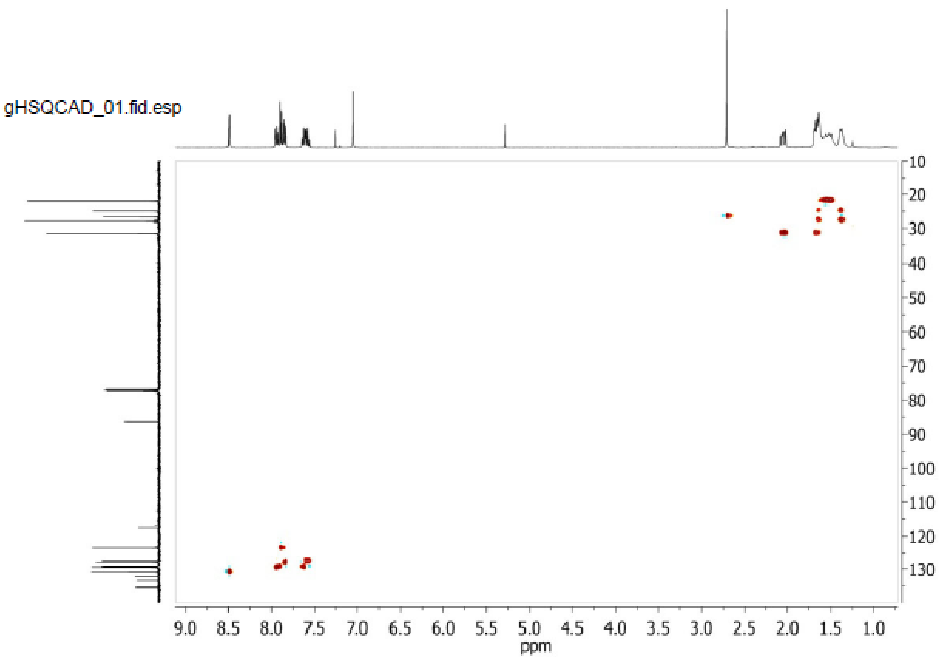

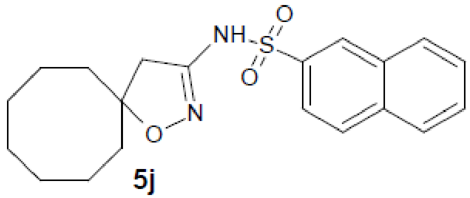

5j

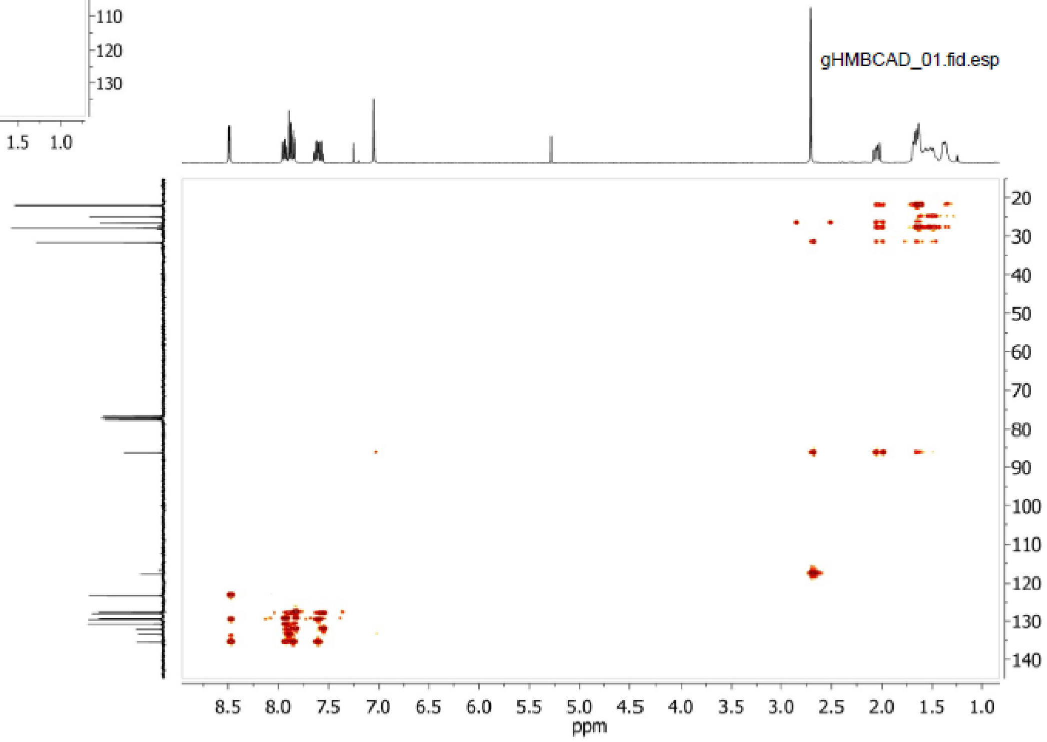

^{1}H NMR ($CDCl_3$) spectrum of compound **5k**

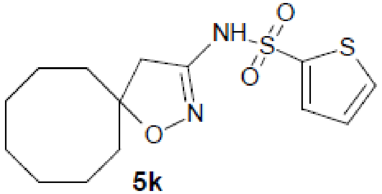

5k

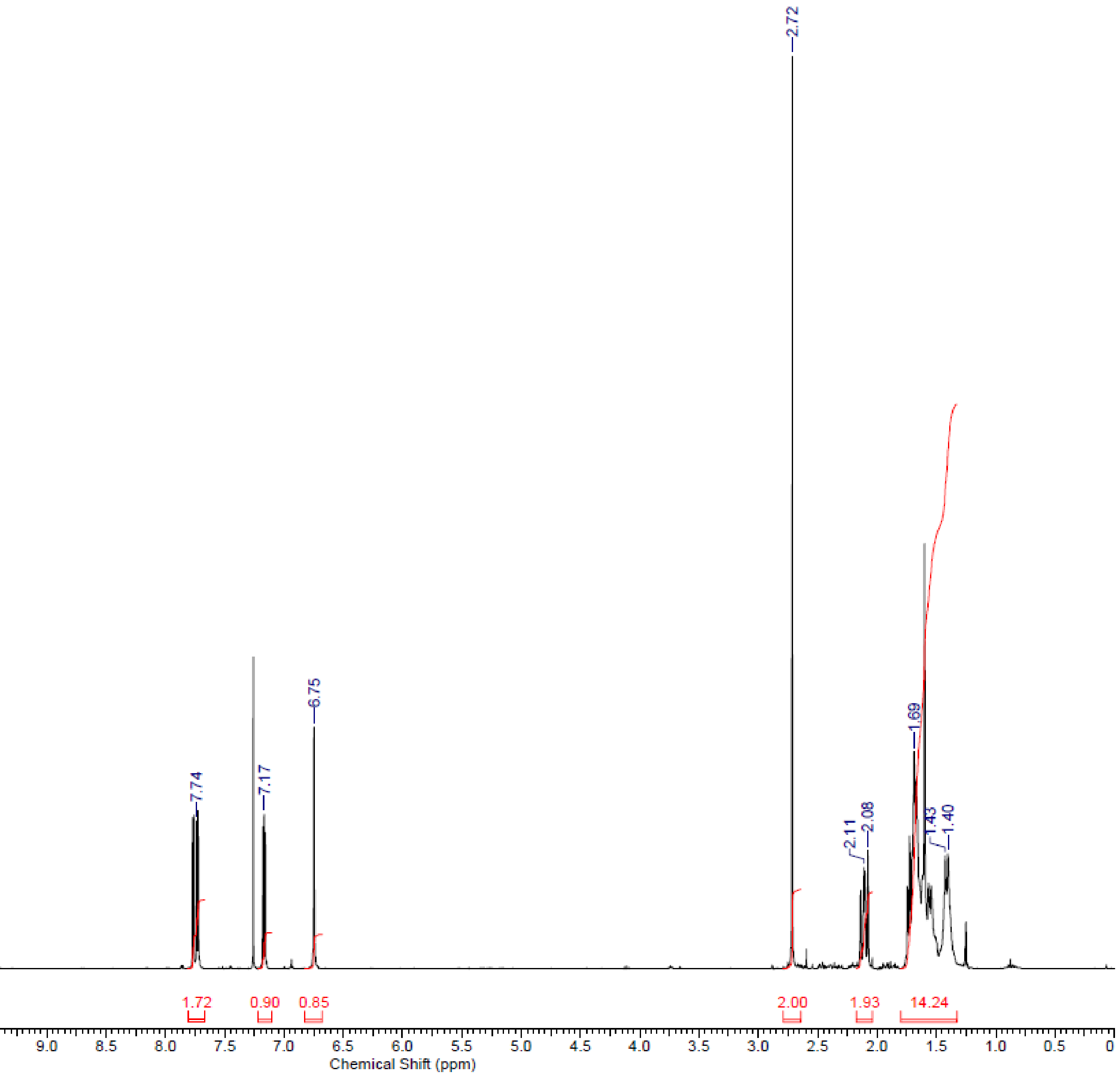

^{13}C NMR ($CDCl_3$) spectrum of compound **5k**

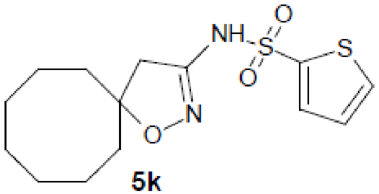

5k

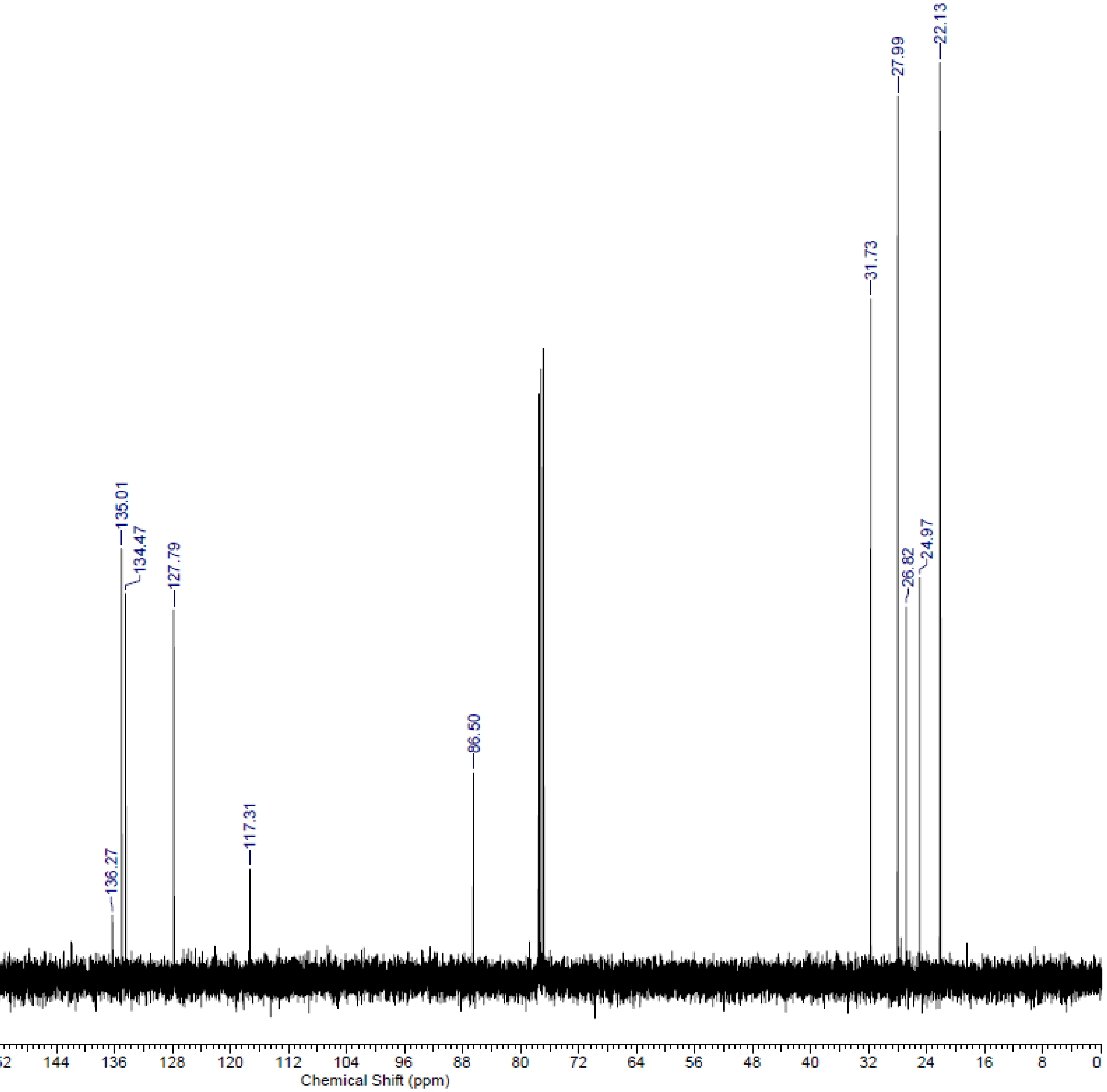

HSQC NMR ($CDCl_3$) spectrum of compound **5k**

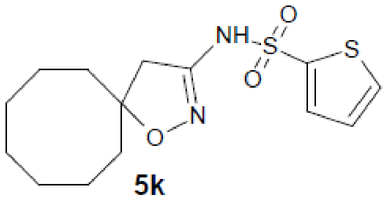

5k

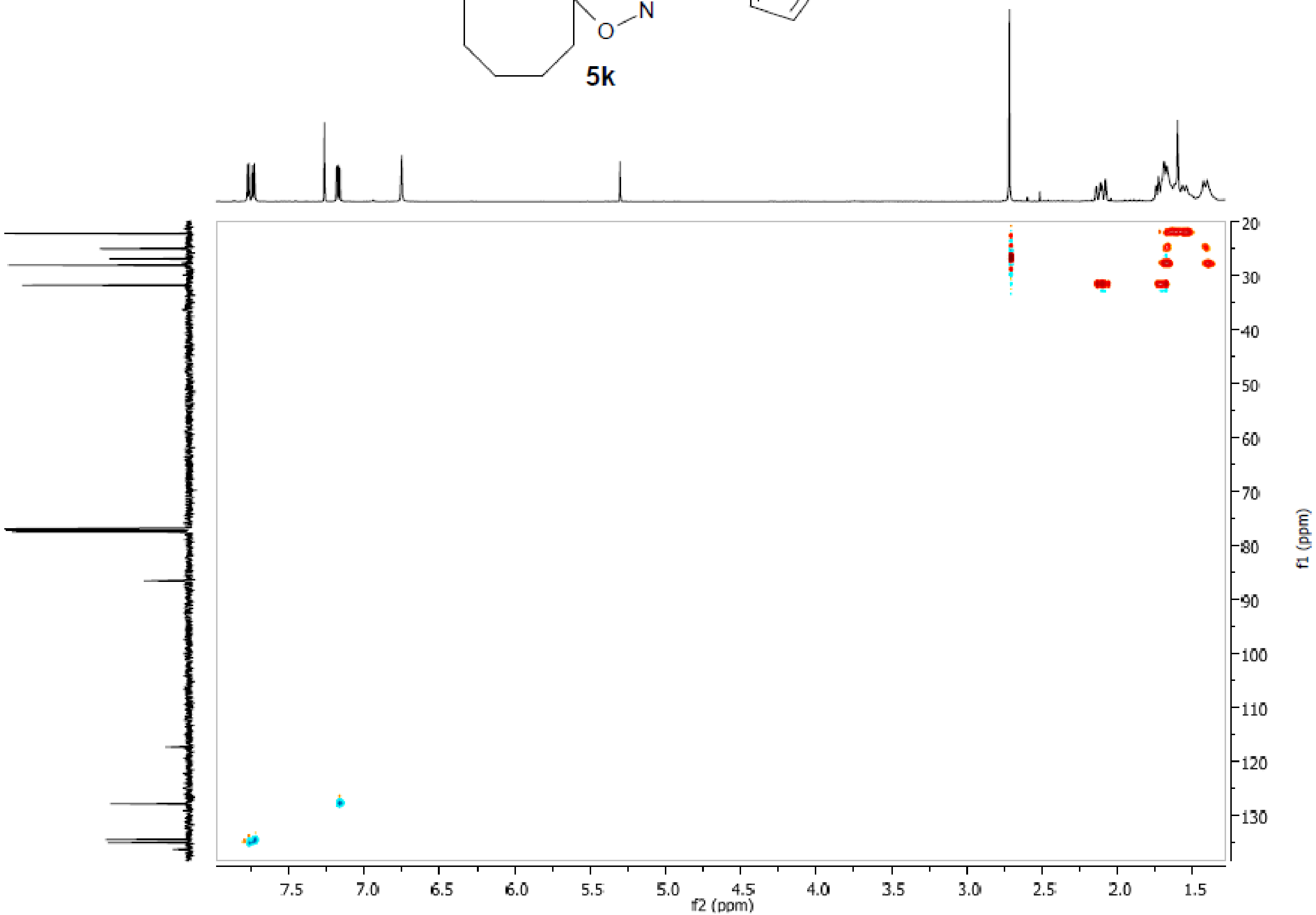

HMBC NMR ($CDCl_3$) spectrum of compound **5k**

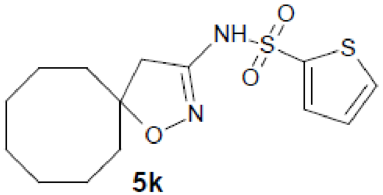

5k

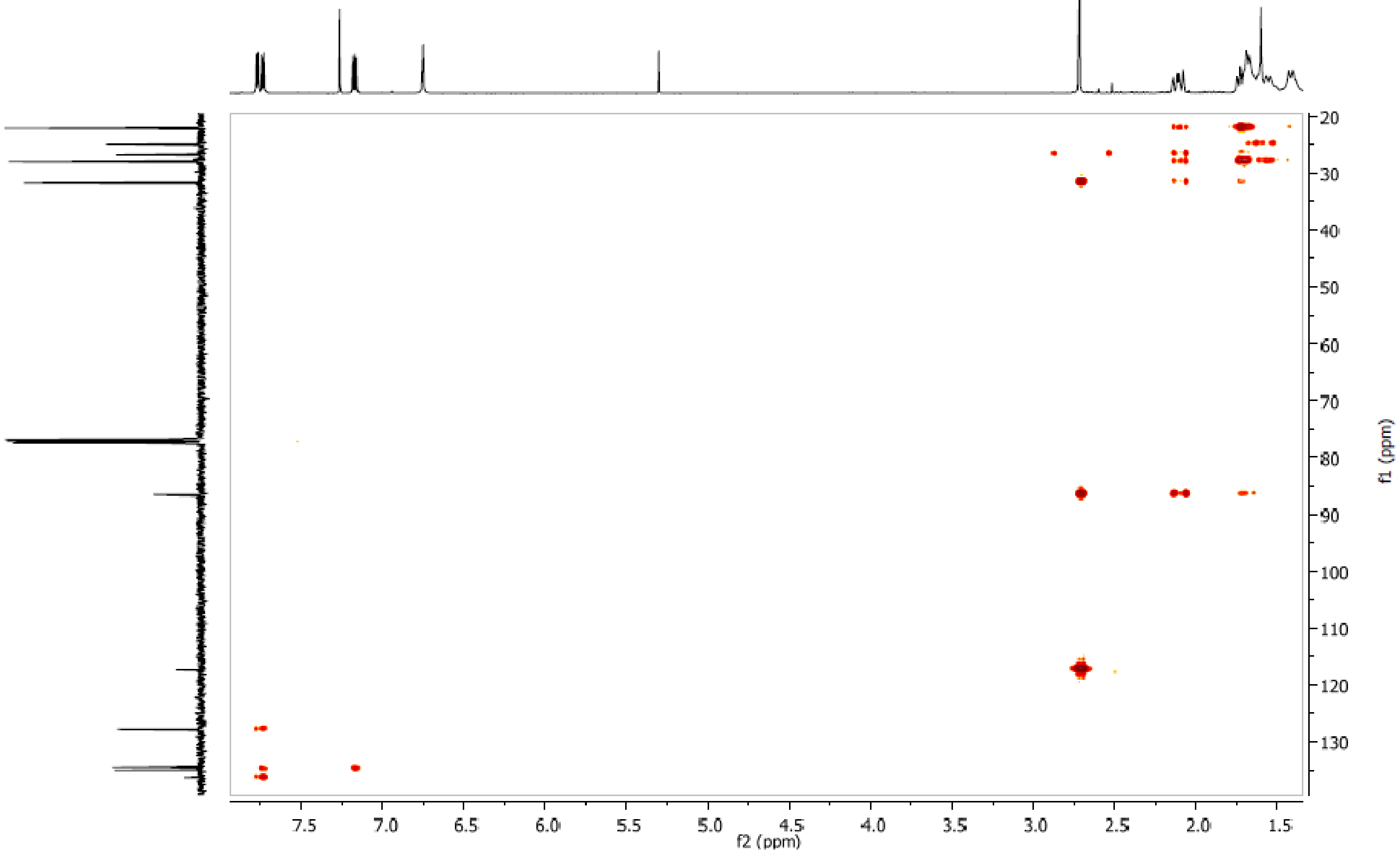

^{1}H NMR ($CDCl_3$) spectrum of compound **5l**

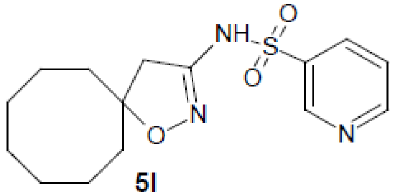

5l

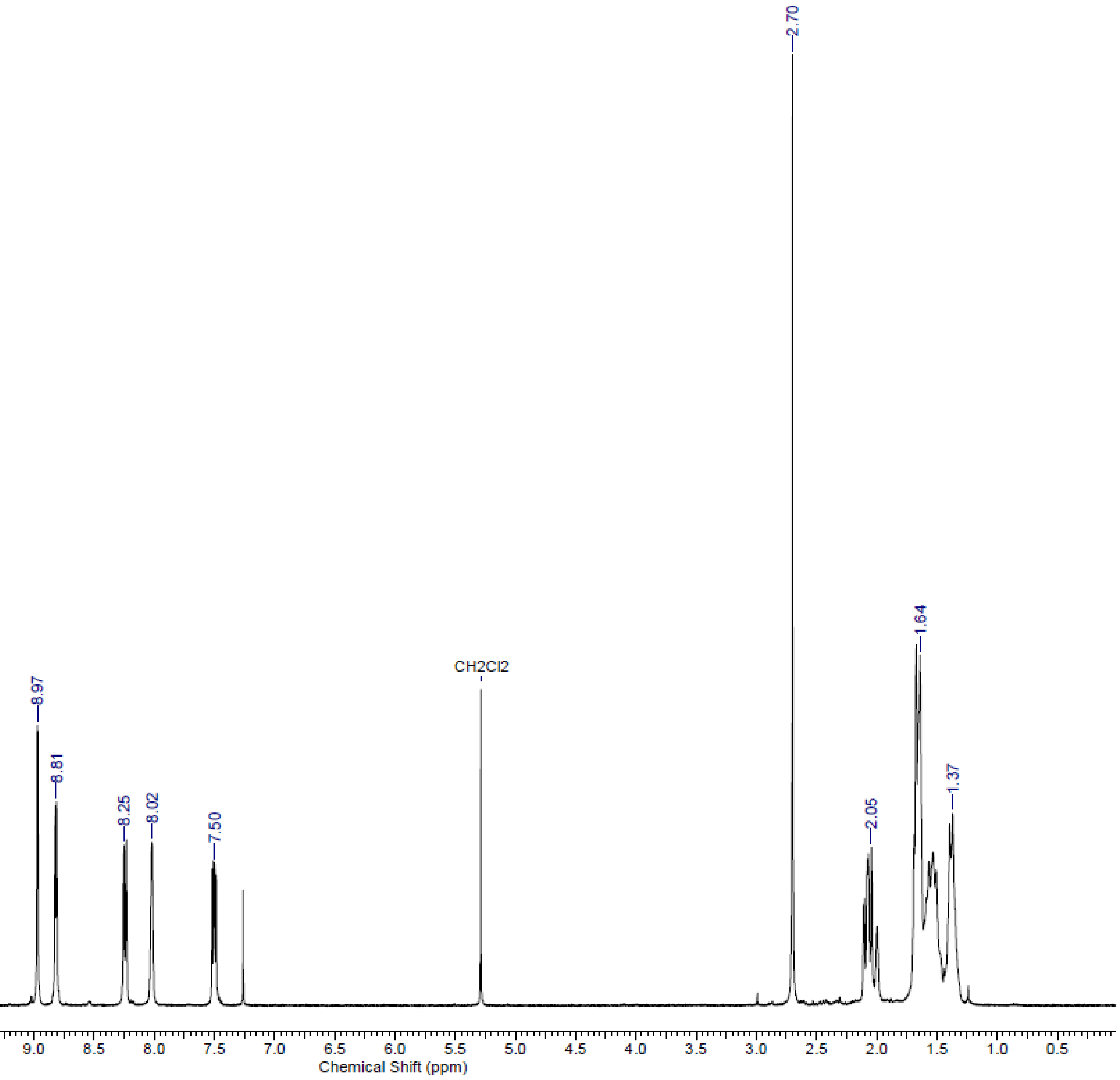

^{13}C NMR ($CDCl_3$) spectrum of compound **5l**

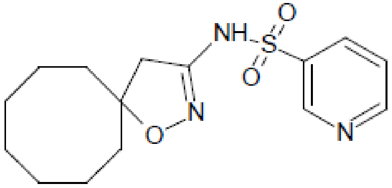

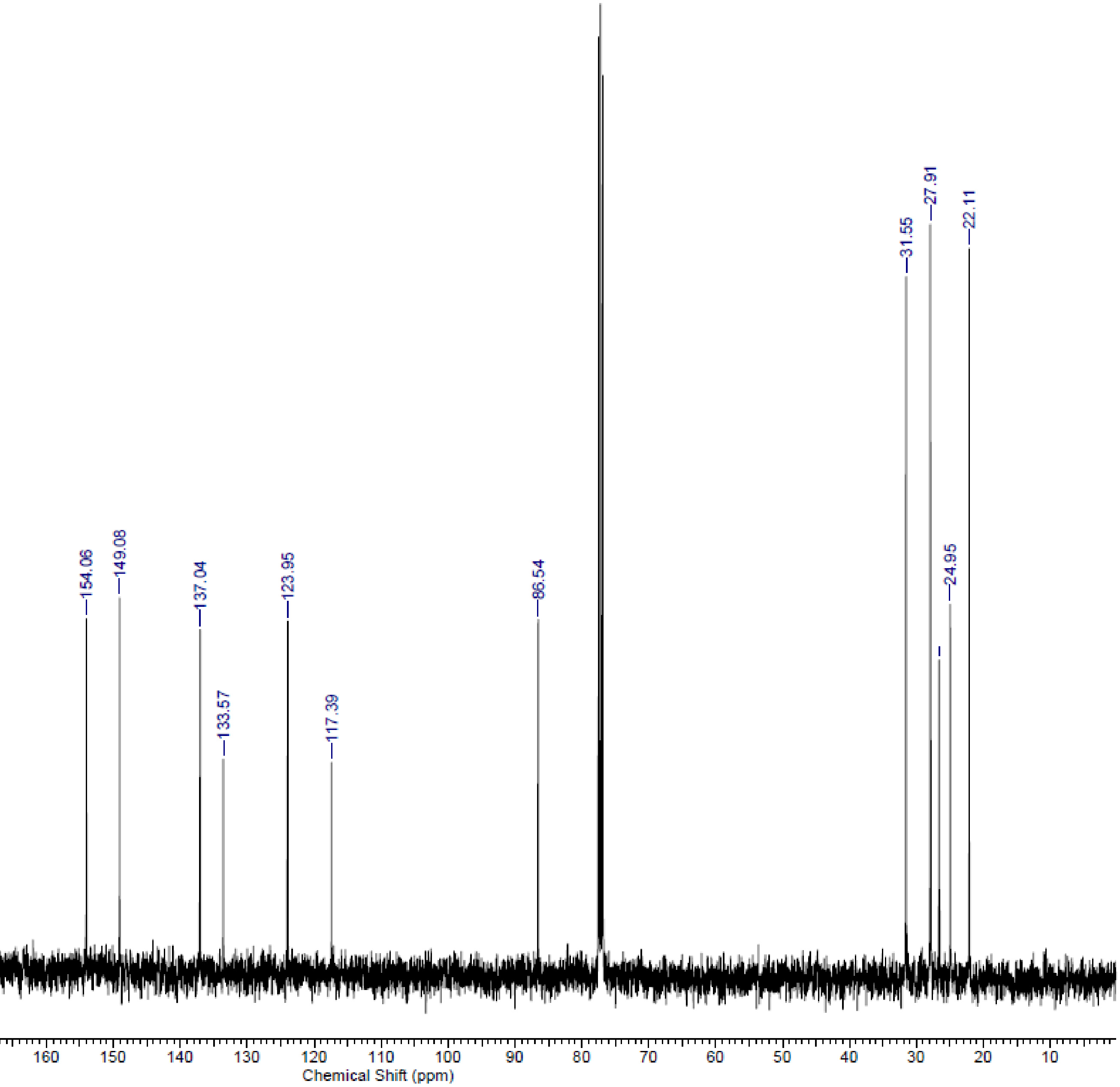

Supplement: Supplementary file 1 [file molecules-27-03546-s001.zip › molecules-1752836-supplementary.pdf]
